# Reclassifying TNM stage I/II colorectal cancer into two subgroups with different overall survival, tumor microenvironment, and response to immune checkpoint blockade treatment

**DOI:** 10.3389/fgene.2022.948920

**Published:** 2022-09-21

**Authors:** Xiangxiang Liu, Jian Qin, Junjie Nie, Huiling Sun, Yuqin Pan, Shukui Wang

**Affiliations:** ^1^ School of Medicine, Southeast University, Nanjing, Jiangsu, China; ^2^ General Clinical Research Center, Nanjing First Hospital, Nanjing Medical University, Nanjing, Jiangsu, China

**Keywords:** colorectal cancer, immune, prognosis, immune checkpoint blockade treatment, single-cell RNA sequencing, TCGA, GEO

## Abstract

**Background:** The traditional TNM staging system is often insufficient to differentiate the survival discrepancies of colorectal cancer (CRC) patients at TNM stage I/II. Our study aimed to reclassify stage I/II CRC patients into several subgroups with different prognoses and explore their suitable therapeutic methods.

**Methods:** Single-cell RNA (scRNA) sequencing data, bulk RNA sequencing data, and clinicopathological information of CRC patients were enrolled from the TCGA and GEO databases. The tumor microenvironment of CRC tissues was accessed by the ESTIMATE algorithm. The prognostic genes were identified by Cox regression analysis. GO and KEGG analyses were conducted in the DAVID database. GSEA analysis was performed for annotation of the correlated gene sets.

**Results:** We successfully reclassified stage I/II CRC patients into two subgroups and discovered that patients in cluster-2 underwent worse overall survival than those in cluster-1. GSEA analysis showed that immune-associated gene sets were positively enriched in cluster-2. Besides, the differentially expressed genes (DEGs) between cluster-1 and cluster-2 patients also participated in immune-related biological processes and signaling pathways. Moreover, we found that more immune cells infiltrated the microenvironment of cluster-2 patients compared to that of cluster-1 patients, such as Tregs and tumor-associated macrophages. ScRNA sequencing analysis uncovered that most of the enriched immune-associated signaling in cluster-2 patients was mainly attributed to these upregulated immune cells whose infiltration levels were also high in CRC tissues rather than in normal tissues. In addition, we demonstrated that the expression of immune checkpoint genes was significantly higher in cluster-2 patients compared to cluster-1 patients. ScRNA sequencing analysis revealed that the infiltrated CD8+T cells in CRC were naïve T cells and can be activated into effector T cells after immune checkpoint blockade (ICB) treatment.

**Conclusion:** TNM stage I/II CRC patients can be divided into two subgroups, which have different overall survival rates, tumor microenvironment, and response to ICB therapy.

## Introduction

Colorectal cancer (CRC) ranks the second leading cause of tumor-related mortality worldwide (Sung et al., 2021). Although the American Joint Committee on Cancer (AJCC) TNM staging system has been widely applied to predict prognosis and formulate therapeutic strategies for CRC patients, it is insufficient to differentiate the survival discrepancies of TNM stage I/II CRC patients. For instance, the prognosis of part CRC patients at stage II was worse than that at stage III (Hari et al., 2013).

Tumor cells live in a complex microenvironment that is composed of various stromal cells, immune cells, extracellular matrix molecules, and cytokines (Wu and Dai, 2017). Mounting studies have proved that the abnormal tumor microenvironment (TME) plays a critical role in the progression and treatment of cancer (Bruni et al., 2020). For example, inducible co-stimulator-activated CD4^+^ T cells are triggers of antitumor immunity in early-stage breast cancer (Zhou et al., 2021). Besides, Shi et al. (2022) systematically profiled a single-cell immune signature to assess anti-PD-1 immunotherapy efficacy of early-stage hepatocellular carcinoma. Although differential gene expression of tumor-infiltrating CD33 myeloid cells in advanced-versus early-stage CRC has been reported (Toor et al., 2021), it remains elusive whether there is a discrepant TME among CRC at TNM stage I/II.

The modality of immune checkpoint blockade (ICB) has revolutionized the treatment of advanced solid tumors over the last decade (Burtness et al., 2019; Mok et al., 2019). Recently, several ongoing clinical trials suggested that integrating ICB into the neoadjuvant treatment of early-stage triple-negative breast cancer and non-small cell lung cancer improved patients’ survival without adding substantial toxicity (Gobbini and Giaj Levra, 2018; Schmid et al., 2020). Whereas there is no literature about ICB treatment in TNM stage I/II CRC. Moreover, due to the heterogeneity of tumors, selecting TNM stage I/II CRC patients who are more suitable for ICB therapy can promote personalized therapy and avoid overtreatment.

In the present study, we discovered that TNM stage I/II CRC patients can be reclassified into two subgroups with different overall survival rates which was mainly attributed to the distinct immune microenvironment of tumors. Moreover, we revealed that this TNM stage I/II CRC patients with poor outcomes owned higher expression levels of immune checkpoint genes and were more suitable for ICB treatment.

## Materials and methods

### Bulk RNA sequencing and ScRNA sequencing analysis

The high-throughput bulk RNA sequencing data and clinicopathological characteristics of CRC patients were downloaded from the TCGA database deposited in the University of California, Santa Cruz (UCSC) Xena browser (https://xenabrowser.net/datapages/) and GEO database (GSE17536 and GSE39582) (Marisa et al., 2013; Chen et al., 2019). The transcription values of genes in the enrolled datasets had been transformed into a normalized count. The single-cell RNA (scRNA) sequencing data of CRC tissues were enrolled from two GEO datasets (GSE146771 and GSE122969) (Kurtulus et al., 2019; Zhang et al., 2020) and analyzed in the public Tumor Immune Single-cell Hub (TISCH) database (http://tisch.comp-genomics.org/home/).

### Functional enrichment analysis

Gene Ontology (GO) analysis and Kyoto Encyclopedia of Genes and Genomes (KEGG) pathway analysis of genes was conducted in the DAVID database (https://david.ncifcrf.gov). The results of the functional enrichment analysis were visualized through an online tool, OmicShare (http://www.omicshare.com/tools). The GSEA analysis was implemented based on the MSigDB database (http://www.gsea-msigdb.org/gsea/msigdb/index.jsp). The enriched gene sets with a *p*-value < 0.05 and FDR value <0.25 were identified to be significant ones.

### Evaluating the infiltration levels of immune and stromal cells

The Estimation of Stromal and Immune cells in Malignant Tumor tissues using Expression data (ESTIMATE) algorithm was used to assess the portion of immune and stromal cells (Yoshihara et al., 2013). The infiltration levels of specific immune cells were estimated by the CIBERSORT, CIBERSORT-ABS, EPIC, and XCELL algorithms. The immune infiltration analysis was performed with the online tool TIMER2 (http://timer.cistrome.org).

### Statistical analysis

Statistical analysis was performed by using GraphPad Prism 8.0 (GraphPad Software, United States) and R software (R 4.1). The Kaplan-Meier (KM) curve with a log rank test was used to compare the significant difference in prognosis between the two groups. The prognostic genes were identified by the univariate Cox regression analysis. The statistical difference between the two groups was analyzed through the Wilcoxon test. *p*-value < 0.05 was considered statistically significant.

## Results

### Reclassifying TNM stage I/II CRC patients into two subgroups with different prognosis

**FIGURE 1:**
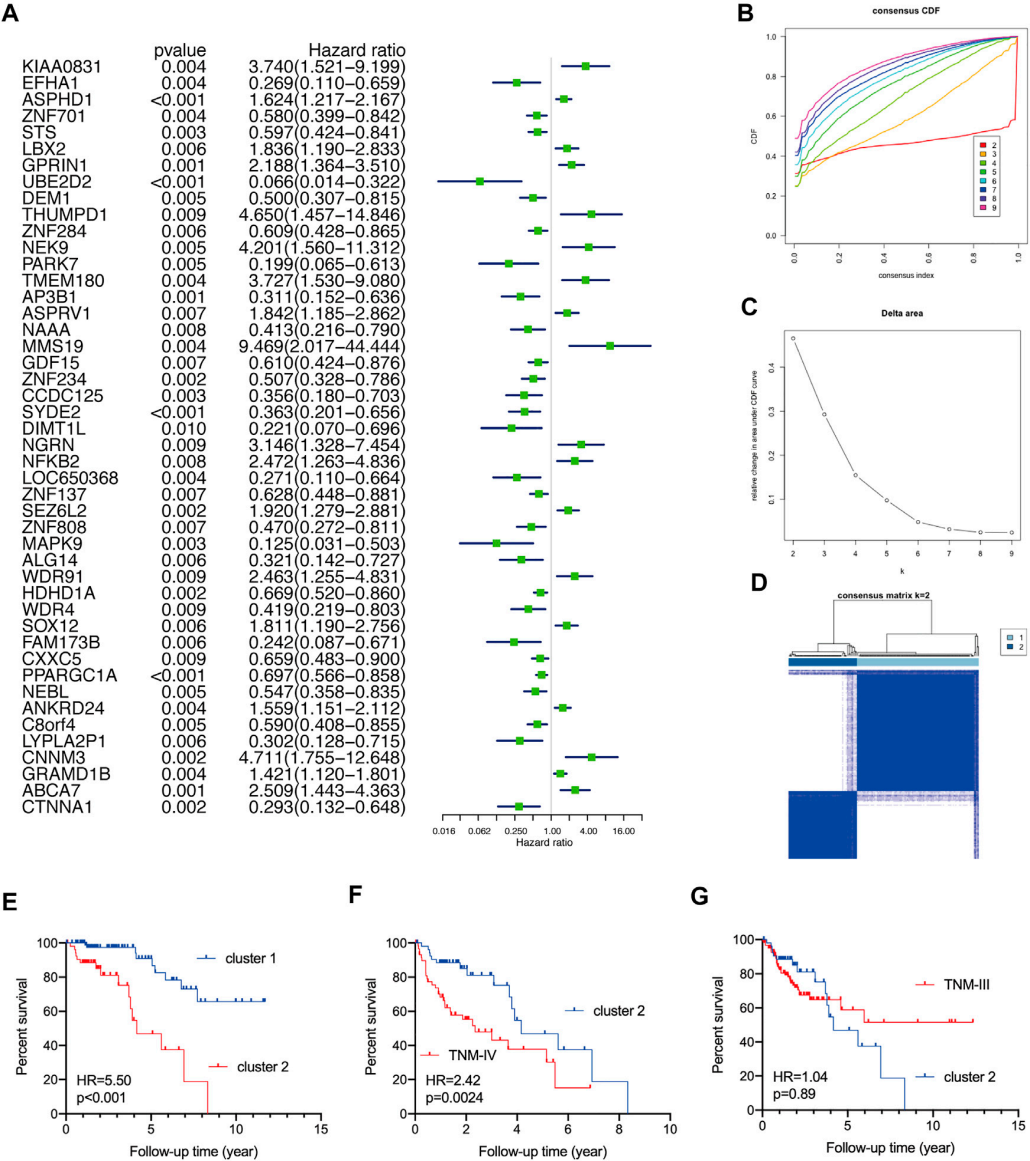
Reclassify TNM stage I/II CRC patients into two novel clusters with different prognoses based on the TCGA CRC cohort. **(A)** The forest plots of prognostic genes with p< 0.01 identified by univariate Cox regression analysi. **(B)** The optimal number of clusters according to the consensus index. **(C)** The optimal clustering stability (k) determined by the proportion of ambiguous clustering measurements. **(D)** Consensus clustering analysis divided TNM stage I/II CRC patients into two subgroups. **(E)** The KM plot curves of TNM stage I/II CRC patients in cluster-1 and cluster-2. **(F)** The KM plot curves of CRC patients in cluster-2 and patients at the TNM-IV stage. **(G)** The KM plot curves of CRC patients in cluster-2 and patients at the TNM-III stage.

We first conducted a univariate Cox regression analysis to screen the prognostic genes among TNM stage I/II CRC patients based on the TCGA CRC cohort and selected 46 prognostic genes with p< 0.01 (Figure 1A). Subsequently, the R package of “Consensus ClusterPlus” was used to test whether these prognostic genes could reclassify TNM stage I/II CRC patients into novel subclusters, and the result indicated that the optimal clustering was two (Figures 1B,C). Based on the unsupervised clustering, this TNM stage I/II CRC patients (N = 153) were well divided into two distinct clusters (Figure 1D). The KM curve analysis showed that stage I/II CRC patients in cluster-2 underwent worse OS than that in cluster-1 (HR = 5.50%, 95%CI: 2.21–13.70, *p* < 0.001) (Figure 1E). Intriguingly, although CRC patients in cluster-2 underwent better OS than patients at the TNM-IV stage (N = 64) (HR = 2.42%, 95%CI: 1.35–4.33, *p* = 0.0024) (Figure 1F), there was no significant difference in the OS between CRC patients in cluster-2 and patients at the TNM-III stage (N = 126) (HR = 1.04%, 95%CI: 0.58–1.87, *p* = 0.89) (Figure 1G).

### Validating the survival discrepancies of TNM stage I/II CRC patients

**FIGURE 2:**
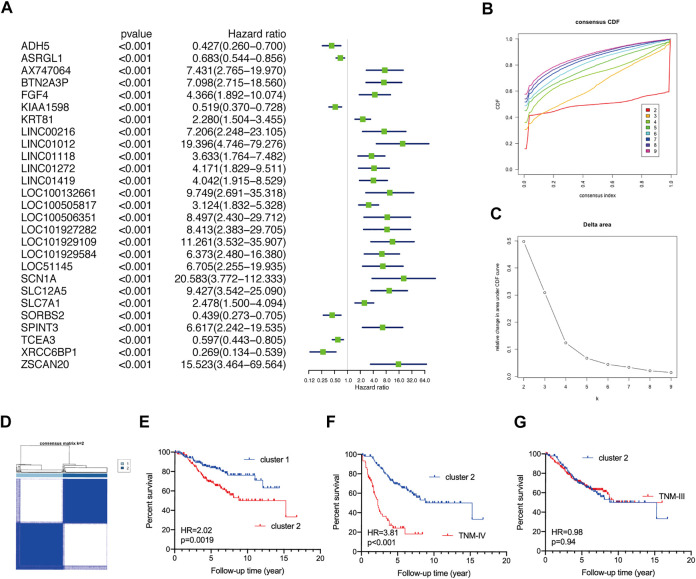
Validate the discrepant prognosis of TNM stage I/II CRC patients based on the GSE39582 dataset. **(A)** The forest plots of prognostic genes with p< 0.001 identified by univariate Cox regression analysis. **(B)** The optimal number of clusters according to the consensus index. **(C)** The optimal clustering stability (k) determined by the proportion of ambiguous clustering measurements. **(D)** Consensus clustering analysis divided TNM stage I/II CRC patients into two subgroups. **(E)** The KM plot curves of CRC patients in cluster-1 and cluster-2. **(F)** The KM plot curves of CRC patients in cluster-2 and patients at the TNM-IV stage. **(G)** The KM plot curves of CRC patients in cluster-2 and patients at the TNM-III stage.

Two GEO datasets (GSE39582 and GSE17536) were implemented to validate the survival discrepancies of TNM stage I/II CRC patients. Firstly, based on univariate Cox regression analysis, 27 prognostic genes (p< 0.001) and 103 prognostic genes (p< 0.01) were identified in GSE39582 and GSE17536, respectively (Figure 2A, Supplementary Figure S1A). The proportion of ambiguous clustering analysis suggested that the lowest clusters were two (Figures 2B,C, Supplementary Figure S1B,C). Based on the unsupervised clustering, TNM stage I/II CRC patients in both GSE39582 (N = 302) and GSE17536 (N = 81) were reclassified into two subgroups (Figure 2D, Supplementary Figure S1D). The KM curve analysis showed that no matter in GSE39582 or GSE17536, TNM stage I/II CRC patients in cluster-2 underwent worse OS than that in cluster-1 (Figure 2E, Supplementary Figure S1E). As expected, analysis of the GSE39582 dataset exhibited that although CRC patients in cluster-2 underwent better OS than patients at the TNM-IV stage (N = 59) (HR = 3.81%, 95%CI: 2.19–6.45, *p* < 0.001) (Figure 2F), there was no significant difference in the OS between CRC patients in cluster-2 and patients at the TNM-III stage (N = 208) (HR = 0.98%, 95%CI: 0.69–1.41, *p* = 0.94) (Figure 2G). Consistently, based on the GSE17536 dataset, CRC patients in cluster-2 underwent better OS than patients at the TNM-IV stage (N = 39) (HR = 3.30%, 95%CI: 1.83–5.94, *p <* 0.001) (Supplementary Figure S1F), but there was no significant difference in the OS between CRC patients in cluster-2 and patients at the TNM-III stage (N = 57) (HR = 0.87%, 95%CI: 0.45–1.70, *p* = 0.67) (Supplementary Figure S1G). Taken together, we demonstrated that TNM stage I/II CRC patients can be reclassified into two novel subgroups with distinct overall survival rates.

### The different immune regulation systems between TNM stage I/II CRC patients in cluster-1 and cluster-2

**FIGURE 3:**
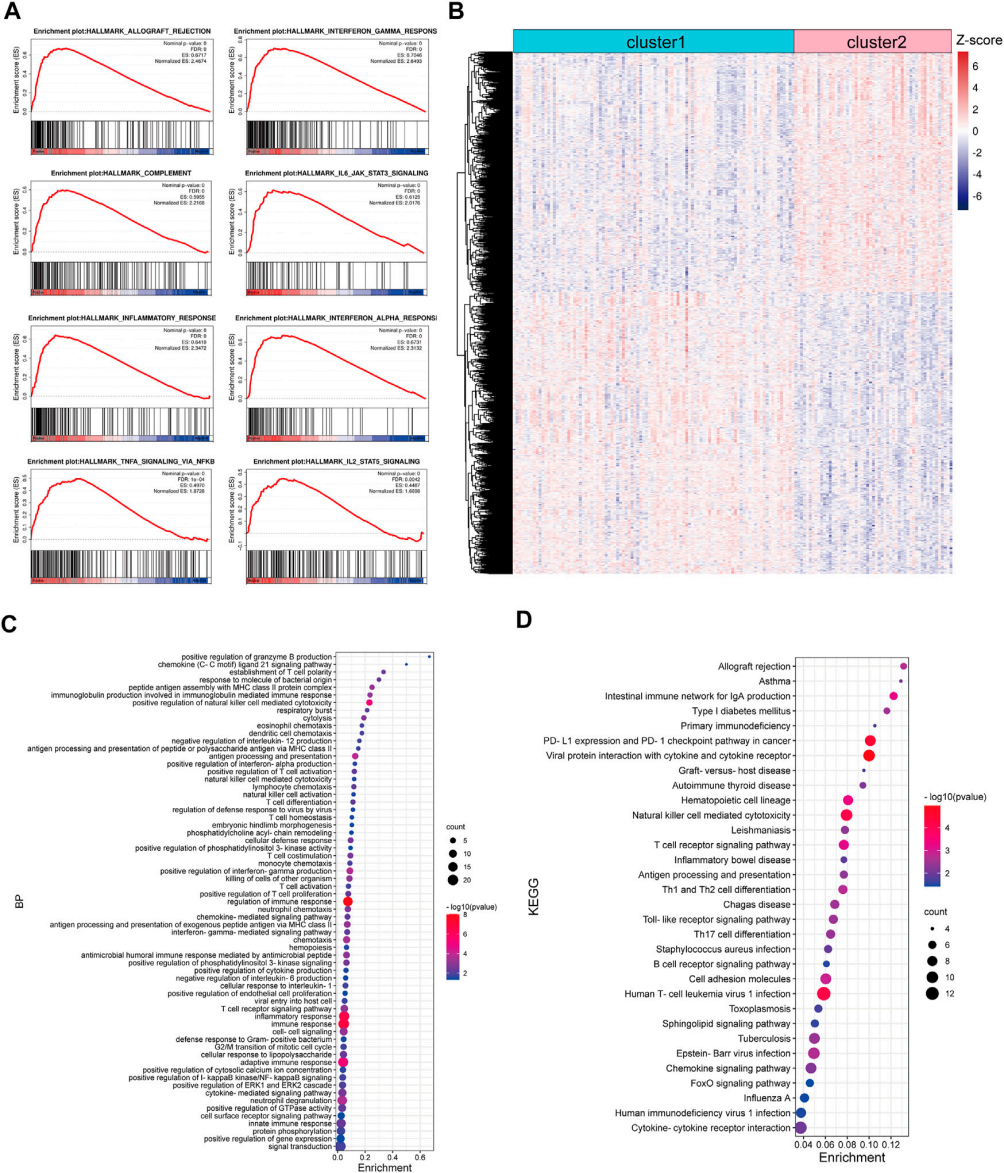
Functional enrichment analysis of DEGs between CRC patients in cluster-1 and cluster-2 based on the TCGA CRC cohort. **(A)** The enriched immune-associated gene sets in CRC patients in cluster-2 revealed by GSEA analysis. **(B)** The heatmap of DEGs between CRC patients in cluster-1 and cluster-2. **(C)** The biological process analysis of DEGs. **(D)** KEGG pathway analysis of DEGs.

Based on the TCGA CRC cohort, we carried out a GSEA analysis to explore the difference between TNM stage I/II CRC patients in cluster-1 and cluster-2, and the results showed that gene sets of HALLMARK_INFLAMMATORY_RESPONSE, HALLMARK_COMPLEMENT, HALLMARK_INTERFERON_GAMMA_RESPONSE, HALLMARK_INTERFERON_ALPHA_RESPONSE, HALLMARK_ALLOGRAFT_REJECTION, HALLMARK_IL6_JAK_STAT3_SIGNALING, HALLMARK_IL2_STAT5_SIGNALING, and HALLMARK_INFLAMMATORY_RESPONSE were positively enriched in CRC patients in cluster-2 (Figure 3A). Subsequently, 2,374 differentially expressed genes (DEGs) with p< 0.001 were identified between CRC patients in cluster-1 and cluster-2 (Figure 3B). Biological process analysis showed that these DEGs were mainly involved in immune regulation, such as innate immune response, T cell co-stimulation, T cell activation, immune effector process, lymphocyte migration, and antigen processing and presentation (Figure 3C). In addition, KEGG pathway analysis exhibited that most DEGs participated in immune-related signaling pathways, such as T cell receptor signaling pathway, natural killer cell-mediated cytotoxicity, PD-L1 expression and PD-1 checkpoint pathway in cancer, and chemokine signaling pathway (Figure 3D).

### Validating the distinct immune regulation systems between TNM stage I/II CRC patients in cluster-1 and cluster-2

**FIGURE 4:**
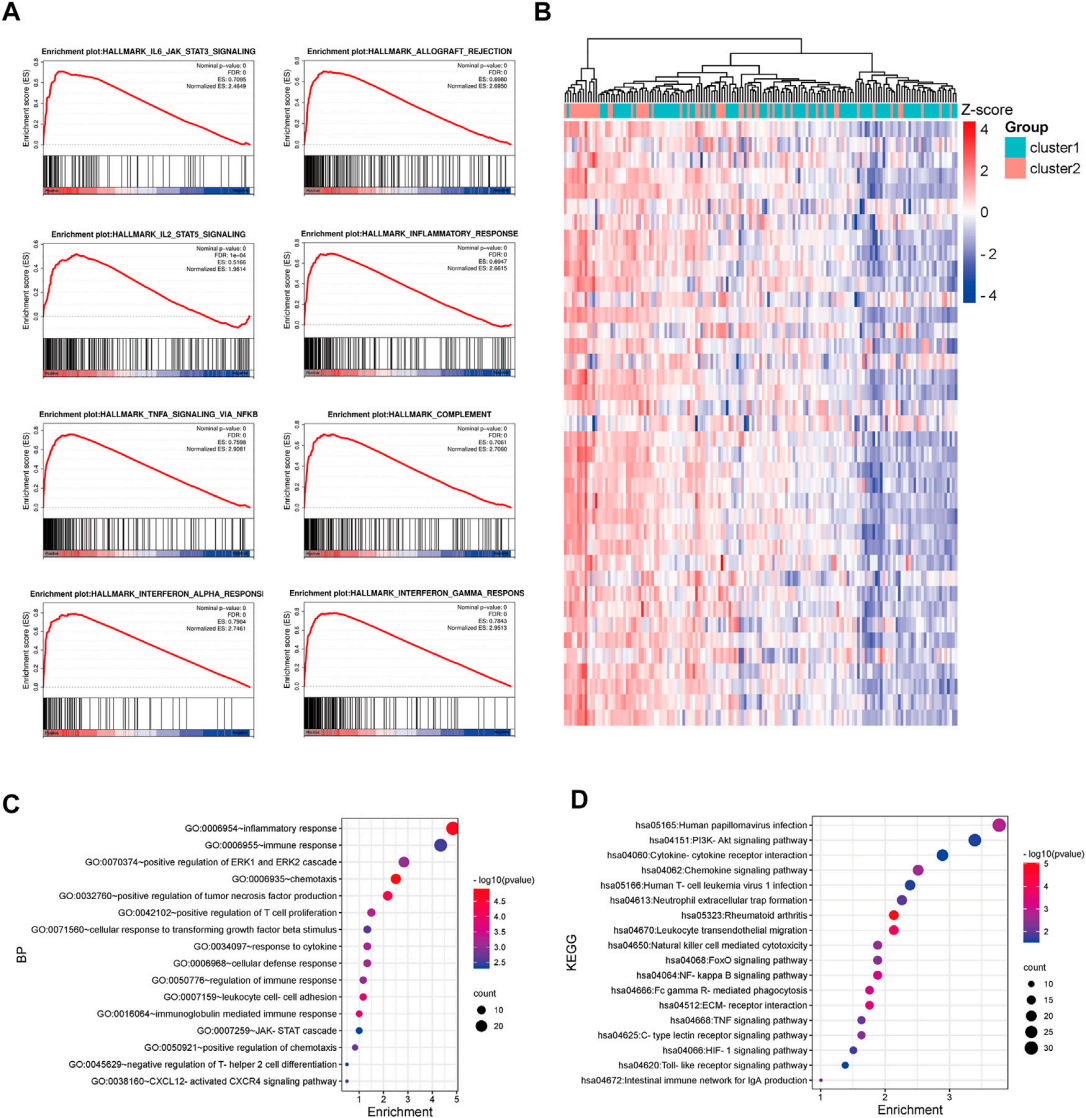
Functional enrichment analysis of DEGs between CRC patients in cluster-1 and cluster-2 based on the GSE17536 dataset. **(A)** The enriched immune-associated gene sets in CRC patients in cluster-2 revealed by GSEA analysis. **(B)** The heatmap of DEGs between CRC patients in cluster-1 and cluster-2. **(C)** The biological process analysis of DEGs. **(D)** KEGG pathway analysis of DEGs.

The GSE17536 and GSE39582 datasets were used to validate the distinct immune regulation between TNM stage I/II CRC patients in cluster-1 and cluster-2. GSEA analysis of GSE17536 showed that the immune-associated gene sets were positively enriched in CRC patients in cluster-2 (Figure 4A). Consistently, GSEA analysis of GSE39582 exhibited that the immune-associated gene sets were negatively correlated with CRC patients in cluster-1 (Supplementary Figure S2A). Subsequently, 1,466 and 3,419 DEGs with p< 0.001 between CRC patients in cluster-1 and cluster-2 were identified in GSE17536 and GSE39582, respectively (Figure 4B, Supplementary Figure S2B). Biological process analysis based on GSE17536 and GSE39582 both showed that part DEGs were also associated with immune regulation, such as positive regulation of T cell proliferation and positive regulation of IL-8 production (Figure 4C, Supplementary Figure S2C). Moreover, KEGG pathway analysis demonstrated that part DEGs in GSE17536 and GSE39582 both participated in immune-related signaling pathways, such as TNF signaling pathway, natural killer cell-mediated cytotoxicity, PD-L1 expression and PD-1 checkpoint pathway in cancer, and chemokine receptor interaction (Figure 4D, Supplementary Figure S2D). Therefore, the different immune regulations were partly responsible for the discrepant prognosis of TNM stage I/II CRC patients.

### The discrepant tumor microenvironments between TNM Stage I/II CRC patients in cluster-1 and cluster-2

**FIGURE 5:**
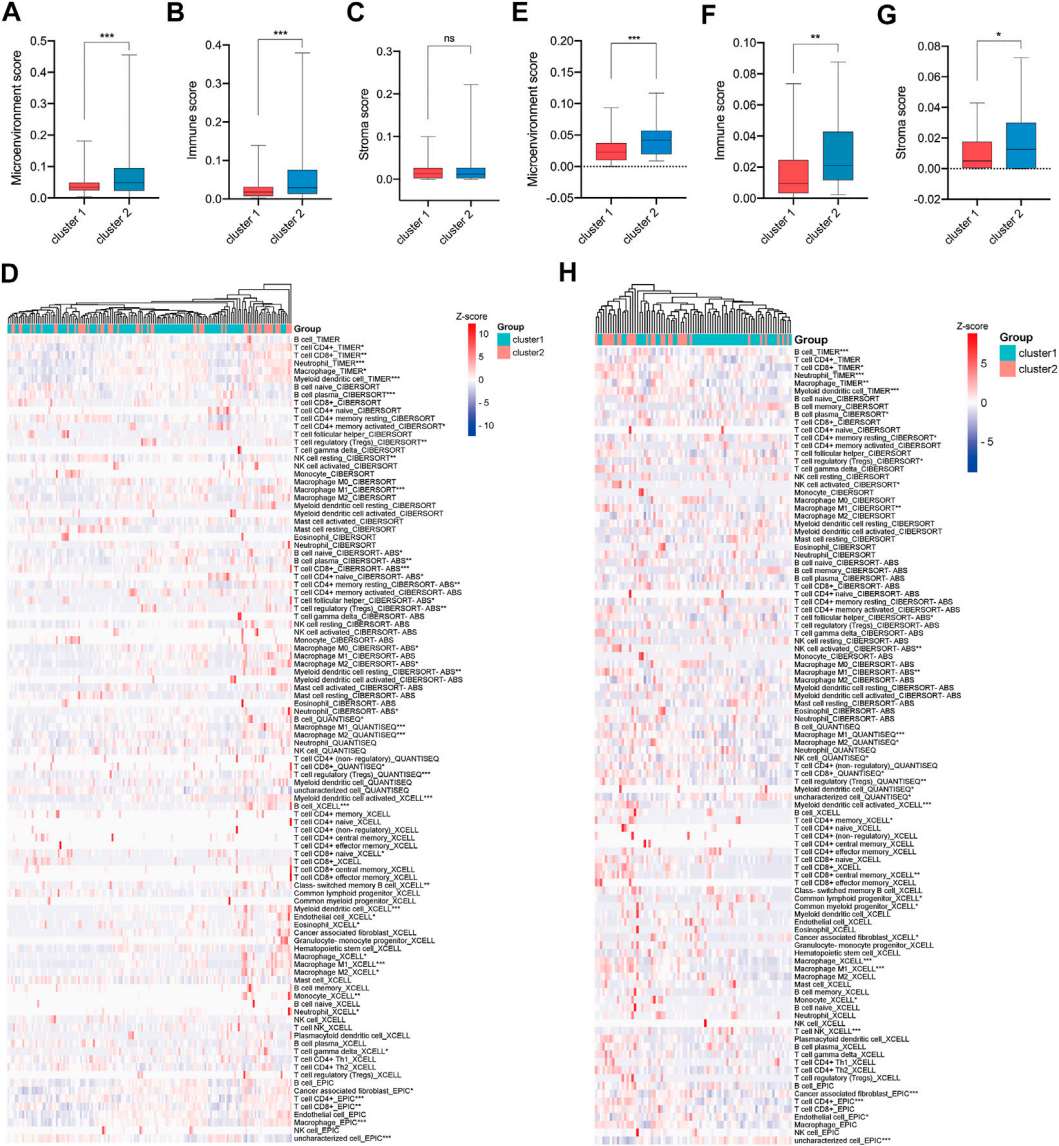
More immune cells infiltrated in tumor tissues of e TNM stage I/II CRC patients in cluster-2 compared with that in cluster-1. **(A)** The microenvironment scores, **(B)** immune scores, **(C)** stromal scores, and **(D)** infiltrated levels of immune cells in the tumors of TNM stage I/II CRC patients in cluster-1 and cluster-2 based on the TCGA CRC cohort. **(E)** The microenvironment scores, **(F)** immune scores, **(G)** stromal scores, and **(H)** infiltrated levels of immune cells in the tumors of CRC patients in cluster-1 and cluster-2 based on the GSE17536 dataset. *, *p* < 0.05; **, *p* < 0.01; ***, *p* < 0.001.

To investigate the tumor microenvironments between CRC patients in cluster-1 and cluster-2, we first calculated their microenvironment scores based on the TCGA CRC cohort. The results showed that the microenvironment scores were significantly upregulated in TNM stage I/II CRC patients in cluster-2 compared with that in cluster-1 (Figure 5A). Subsequently, we analyzed the immune scores and stromal scores. Intriguingly, we found the immune scores, rather than stromal scores, were significantly elevated in CRC patients in cluster-2 (Figures 5B,C). Next, we compared the infiltrated levels of immune cells between the two groups based on four algorithms. As shown in Figure 5D, most immune cells were significantly enriched in CRC patients in cluster-2, such as CD8^+^ T cells, Tregs, resting NK cells, tumor-associated macrophages (TAMs), and resting mast cells.

To validate our findings, the GSE17536 dataset was analyzed. As shown in Figures 5E–G, the microenvironment scores, immune scores, and stromal scores were significantly upregulated in TNM stage I/II CRC patients in cluster-2 compared with that in cluster-1. In line with the above results, the infiltrated levels of most immune cells were significantly higher in CRC patients in cluster-2 than those in cluster-1 (Figure 5H). Overall, our results indicated that the different prognoses of TNM stage I/II CRC patients in two subgroups were partly attributed to their distinct tumor microenvironment.

### Exploring the dysregulated immune signaling and differentially infiltrated immune cells in CRC through ScRNA sequencing

**FIGURE 6:**
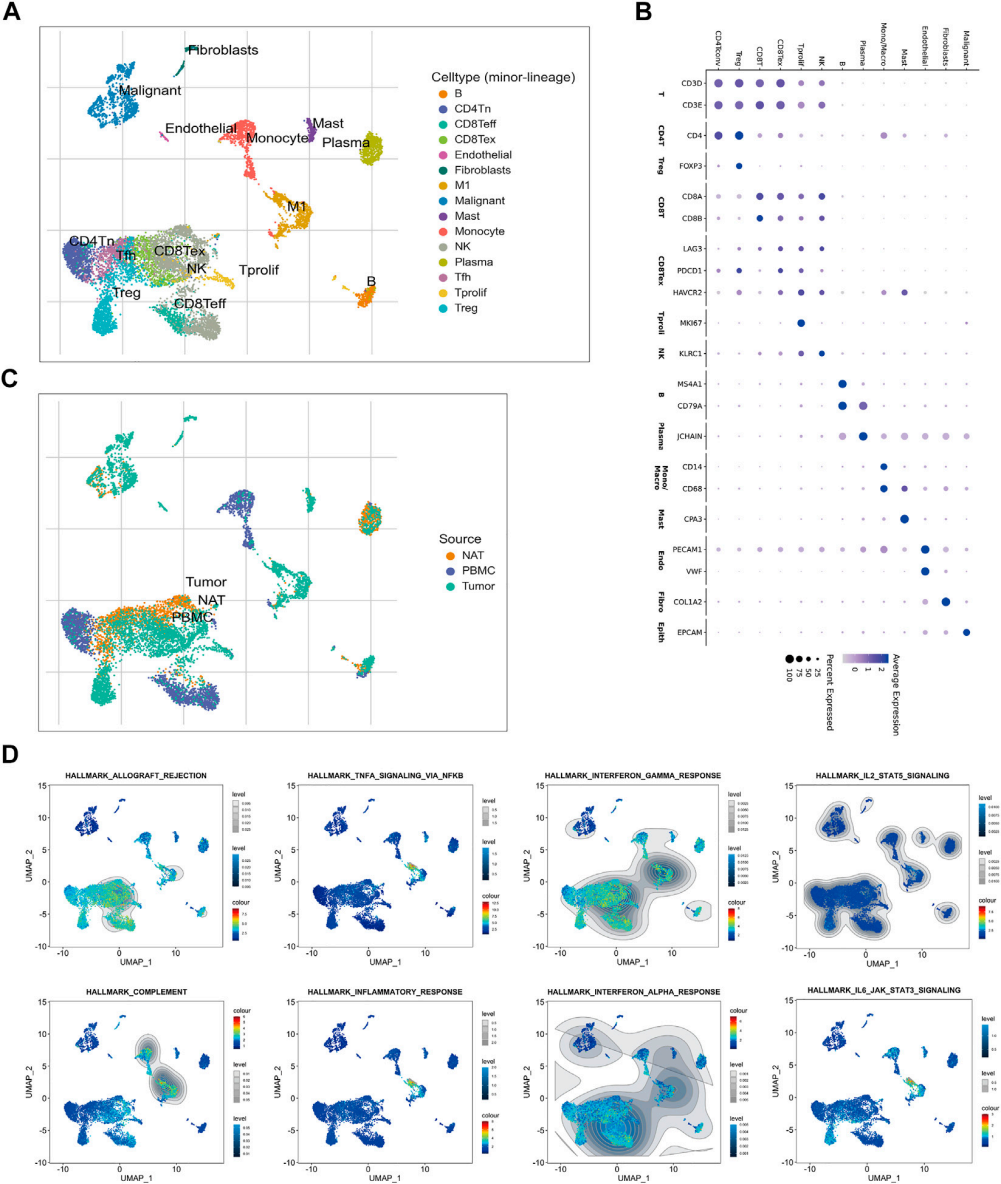
Sc-RNA sequencing of the dysregulated immune signaling and differentially infiltrated immune cells in CRC tissues. **(A)** The identified 15 subtypes of cells collected from normal adjacent tissue (NAT), CRC tissue, and peripheral blood mononuclear cell (PBMC). **(B)** The hallmark genes of each cell subtype. **(C)** The enrichment of each cell subtype in NAT, CRC tissue, and PBMC. **(D)** The distribution of immune-associated gene sets in each cell subtype identified by single-cell GSEA analysis.

A single-cell RNA (scRNA) sequencing data enrolled from GSE146771 were analyzed to further explore the dysregulated immune signaling and differentially infiltrated immune cells in CRC. Firstly, 10,468 cells collected from normal adjacent tissue (NATs), CRC tissues, and peripheral blood mononuclear cells (PBMC), were well divided into 15 cell subtypes and the corresponding marker genes were exhibited (Figures 6A,B). Intriguingly, these immune cells, such as Tregs, CD8+T cells, B cells, TAMs, and mast cells, whose infiltration levels were significantly elevated in TNM stage I/II CRC patients of cluster-2 were also enriched in CRC tissues compared to NATs (Figure 6C). Our previous GSEA analysis based on bulk RNA sequencing data exhibited that several immune-associated gene sets were positively enriched in cluster-2. Interestingly, scRNA sequencing analysis revealed that these gene sets were enriched in these immune cells whose infiltration levels were significantly elevated in CRC tissues and in CRC patients of cluster-2, including HALLMARK_INFLAMMATORY_RESPONSE, HALLMARK_COMPLEMENT, HALLMARK_INTERFERON_GAMMA_RESPONSE, HALLMARK_INTERFERON_ALPHA_RESPONSE, HALLMARK_ALLOGRAFT_REJECTION, HALLMARK_IL6_JAK_STAT3_SIGNALING, HALLMARK_IL2_STAT5_SIGNALING, and HALLMARK_INFLAMMATORY_RESPONSE (Figure 6D). Our scRNA sequencing analysis demonstrated that the dysregulated immune signaling was mainly enriched in differentially infiltrated immune cells in CRC patients of cluster-2.

### TNM stage I/II CRC patients in cluster-2 were more suitable for ICB treatment

**FIGURE 7:**
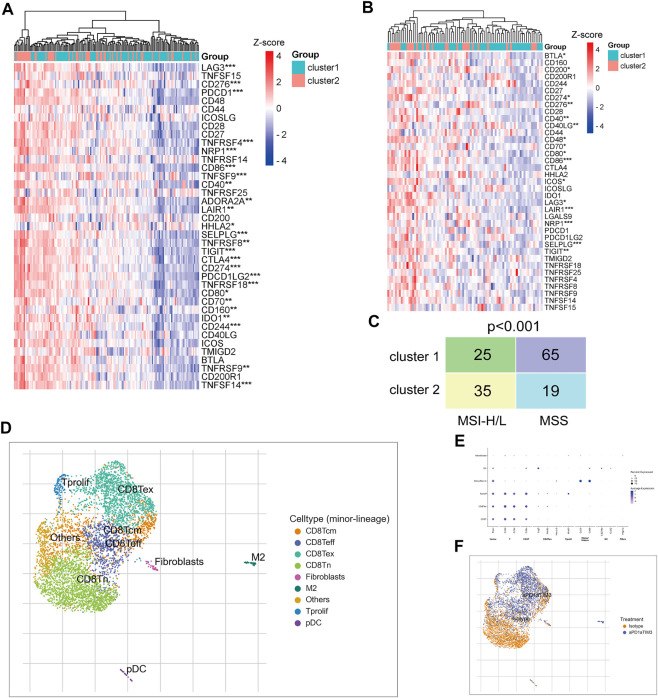
TNM stage I/II CRC patients in cluster-2 were more suitable for ICB treatment. **(A)** The heatmap of differentially expressed immune checkpoint genes between CRC patients in cluster-1 and cluster-2 based on the TCGA CRC cohort. **(B)** The heatmap of differentially expressed immune checkpoint genes between CRC patients in cluster-1 and cluster-2 based on the GSE122969 dataset. **(C)** The Chi-square test of MSI status between TNM stage I/II CRC patients in cluster-1 and cluster-2 based on the TCGA CRC cohort. **(D)** The identified nine subtypes of immune cells collected from xenograft before and after ICB treatment. **(E)** The hallmark genes of each subtype of immune cells. **(F)** The change of cell subtypes in xenograft before and after ICB treatment. *, *p* < 0.05; **, *p* < 0.01; ***, *p* < 0.001.

Subsequently, we examined the expression of immune checkpoint genes. Analysis of the TCGA CRC cohort and GSE17536 dataset revealed that most immune checkpoint genes were significantly upregulated in TNM stage I/II CRC patients of cluster-2 compared with that of cluster-1 (Figures 7A,B). In addition, the Chi-square test revealed that CRC patients in cluster-2 own more high or low microsatellite instability (MSI-H/L) status and less microsatellite stability (MSS) status compared to those in cluster-1 (*p* < 0.001) (Figure 7C). Given the high infiltrated levels of CD8+T cells in cluster-2 patients, based on scRNA sequencing data of the GSE122969 dataset, we simulated the changes of CD8+T cells in tumors before and after immune checkpoint blockade (ICB) treatment. Firstly, 5,457 immune cells achieved from the tumor-bearing (MC38 cells) mice before and after anti-PD-1/TIM3 treatment were well divided into nine cell subtypes (Figure 7D). The corresponding marker genes of cells were exhibited in Figure 7E. As shown in Figure 7F, the infiltrated CD8+T cells in CRC tissues were naïve CD8^+^ T cells (CD8Tn) and after ICB treatment, more central memory CD8^+^ T cells (CD8Tcm), effective CD8^+^ T cells (CD8Teff), and exhausted CD8^+^ T cells (CD8Tex) were enriched in tumor tissues. Taken together, our results uncovered that TNM stage I/II CRC patients in cluster-2 were more suitable for ICB treatment.

## Discussion

For decades, the AJCC TNM classification system provides a guideline for surgical resection, adjuvant chemotherapy, as well as patient outcomes for a variety of cancers (Locker et al., 2006). However, unusual clinical outcomes are often observed in patients at TNM stage I/II (Nagtegaal et al., 2011; Cadiz et al., 2018). For instance, the prognosis of part CRC patients at stage II was worse than that at stage III (Hari et al., 2013). In this study, we verified that TNM stage I/II CRC patients can be well divided into two novel subgroups with distinct overall survival rates. Besides, there was even no difference in prognosis between CRC patients in cluster-2 and advanced CRC patients. Therefore, the therapeutic strategy for TNM stage I/II CRC patients in cluster-2 should be different from that in cluster-1.

Emerging evidence uncovered that the tumor microenvironment plays a critical role in tumor progression and that the pre-existing antitumor adaptive immune reaction is vital for patient survival (Galon et al., 2006). For example, tumor cells can enhance macrophage-mediated immunosuppression and subsequently suppress CD8^+^ T cytotoxic function to accelerate metastasis (Zhuang et al., 2020). Similarly, our results revealed that the DEGs between TNM stage I/II CRC patients in cluster-1 and cluster-2 mainly participated in immune-related biological processes and signaling pathways. Subsequently, we discovered that more immune cells infiltrated the tumor tissues of CRC patients in cluster-2 compared with that in cluster-1, such as Treg cells, mast cells, TAMs, CD8^+^ T cells, and B cells. Treg cells suppress abnormal/excessive immune responses to maintain immune homeostasis (Kumar et al., 2020). Treg cells are often involved in tumor development and progression by inhibiting antitumor immunity (Ohue and Nishikawa, 2019). TAMs are also critical regulators of tumors and are significantly associated with metastasis and drug resistance of cancer cells (Guan et al., 2021; Ma et al., 2021). Recently, the advances in macrophage-based cancer immunotherapy have attracted more and more attention (Baradaran et al., 2022). For example, Wang et al. have constructed an engineering endogenous TAM-targeted biomimetic system to reprogram tumor immunosuppressive microenvironment and enhance chemo-immunotherapy (Wang et al., 2021).

As we know, active CD8^+^ T cells bind and kill tumor cells by secreting granzymes, perforin, and cathepsin C (Basu et al., 2016). Interestingly, the infiltration levels of CD8^+^ T cells were also markedly upregulated in TNM stage I/II CRC patients of cluster-2 whose prognosis was poor. ScRNA sequencing technology provided a possibility to deeply analyze the subtypes of various cells which often changed the traditional opinions. For example, oncogenic and tumor-suppressing fibroblasts and macrophages were uncovered in the same tumor tissues (Sebastian et al., 2020; Liang et al., 2021). Based on scRNA sequencing, our study revealed that most infiltrated CD8^+^ T cells in tumor tissues were exhausted CD8^+^ T cells that have lost their cytotoxicity. Tumors can induce CD8^+^ T cell exhaustion and inhibit its activation via expressing immune escape factors, such as PD-L1 (Zhang et al., 2018). Recently, the antibody-based ICB treatment has been applied to improve CD8^+^ T cells’ priming ability and to establish a durable and efficient antitumor immunity (Borst et al., 2018). ICB does not act on the tumor cell itself but directs membrane ligands or receptors to enhance T cell response (van de Ven and Borst, 2015). We discovered that most immune checkpoint genes were upregulated in TNM stage I/II CRC patients in cluster-2, suggesting that these patients may be more suitable for ICB treatment. To validate our hypothesis, we then simulated the changes of CD8^+^ T cells in CRC tissues before and after ICB treatment based on scRNA-sequencing. We found that after ICB treatment, more activated CD8 + T cells (CD8Tcm and CD8Teff) infiltrated tumor tissues. Although our study provided theoretical support, whether ICB treatment could improve the prognosis of TNM stage I/II CRC patients in cluster-2 should be further investigated in clinical trials.

Indeed, there were several limitations in our study. First, although multiple independent datasets were enrolled to confirm the correctness of the data, it was better to personally detect these parameters. Second, it is necessary to consider the expense and the testing period about the classification when our findings were applied to clinical practice. Third, whether TNM stage I/II CRC patients in cluster-2 were more suitable for ICB treatment should be further validated in clinical.

In conclusion, based on bulk RNA sequencing and scRNA sequencing, we first reclassified CRC patients at TNM stage I/II into two novel subgroups with different overall survival rates, tumor microenvironment, and response to ICB treatment.

## Data Availability

The datasets presented in this study can be found in online repositories. The names of the repository/repositories and accession number(s) can be found in the article/Supplementary Material.
