# Supplementary figures and images for: Reclassifying TNM stage I/II colorectal cancer into two subgroups with different overall survival, tumor microenvironment, and response to immune checkpoint blockade treatment (part 1 of 2)
Source: Front Genet. 2022 Sep 21;13:948920. doi: 10.3389/fgene.2022.948920 (PMC9532767; doi:10.3389/fgene.2022.948920)

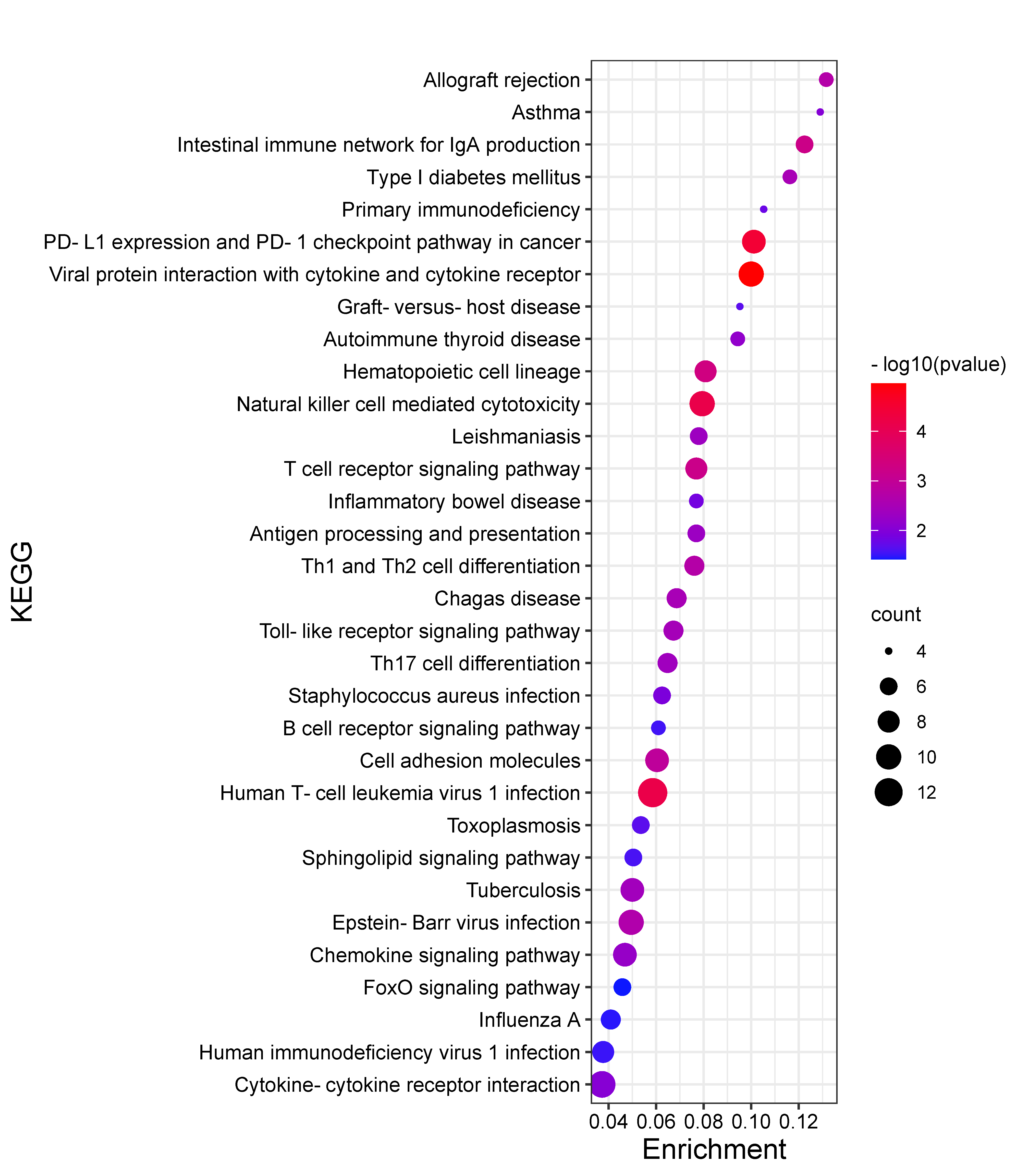

Supplement: Supplementary file 1 [file DataSheet3.ZIP › datasheet of Figure 3/TCGAgokegg/1650423256.21.tiff]

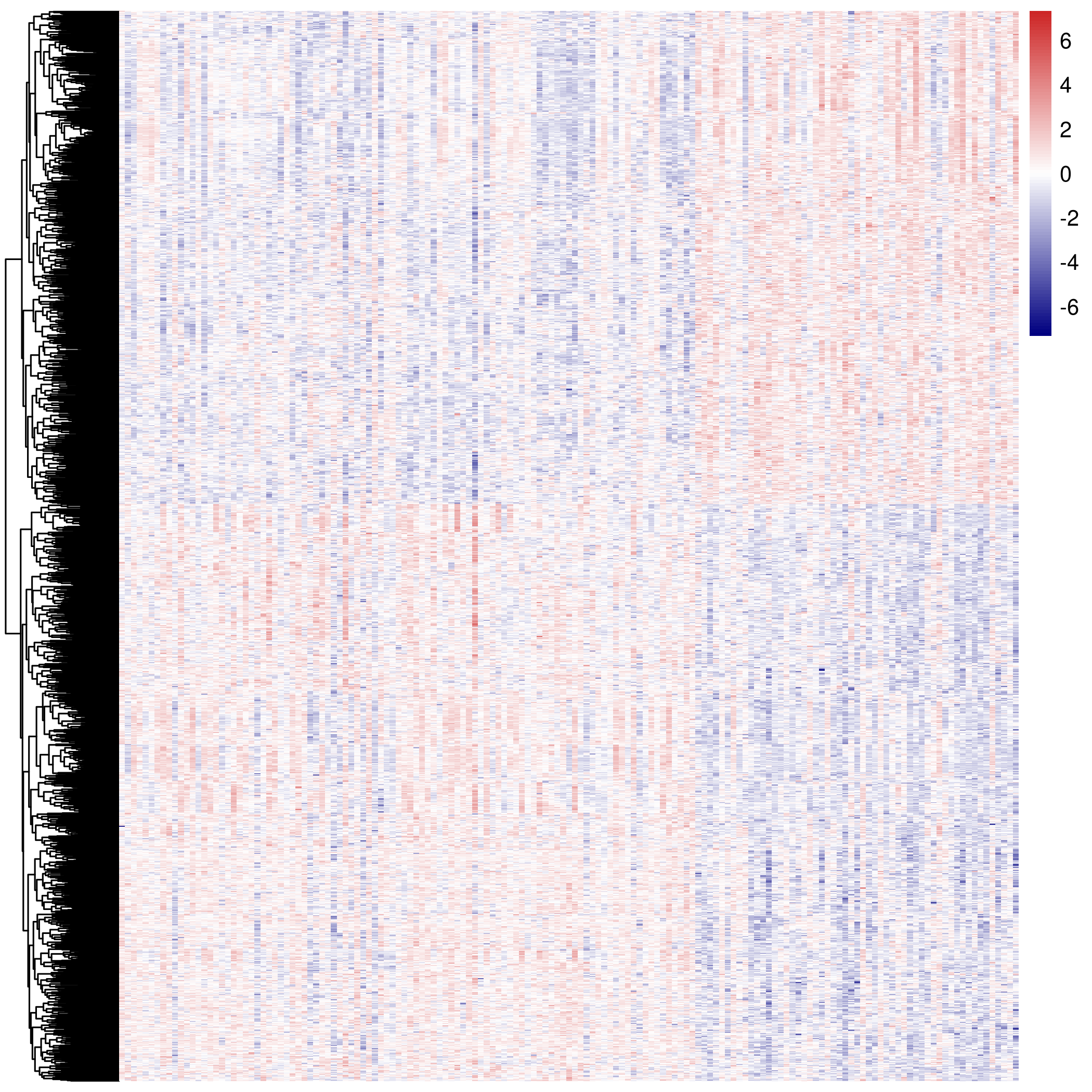

Supplement: Supplementary file 1 [file DataSheet3.ZIP › datasheet of Figure 3/TCGAgokegg/pheatmap.png]

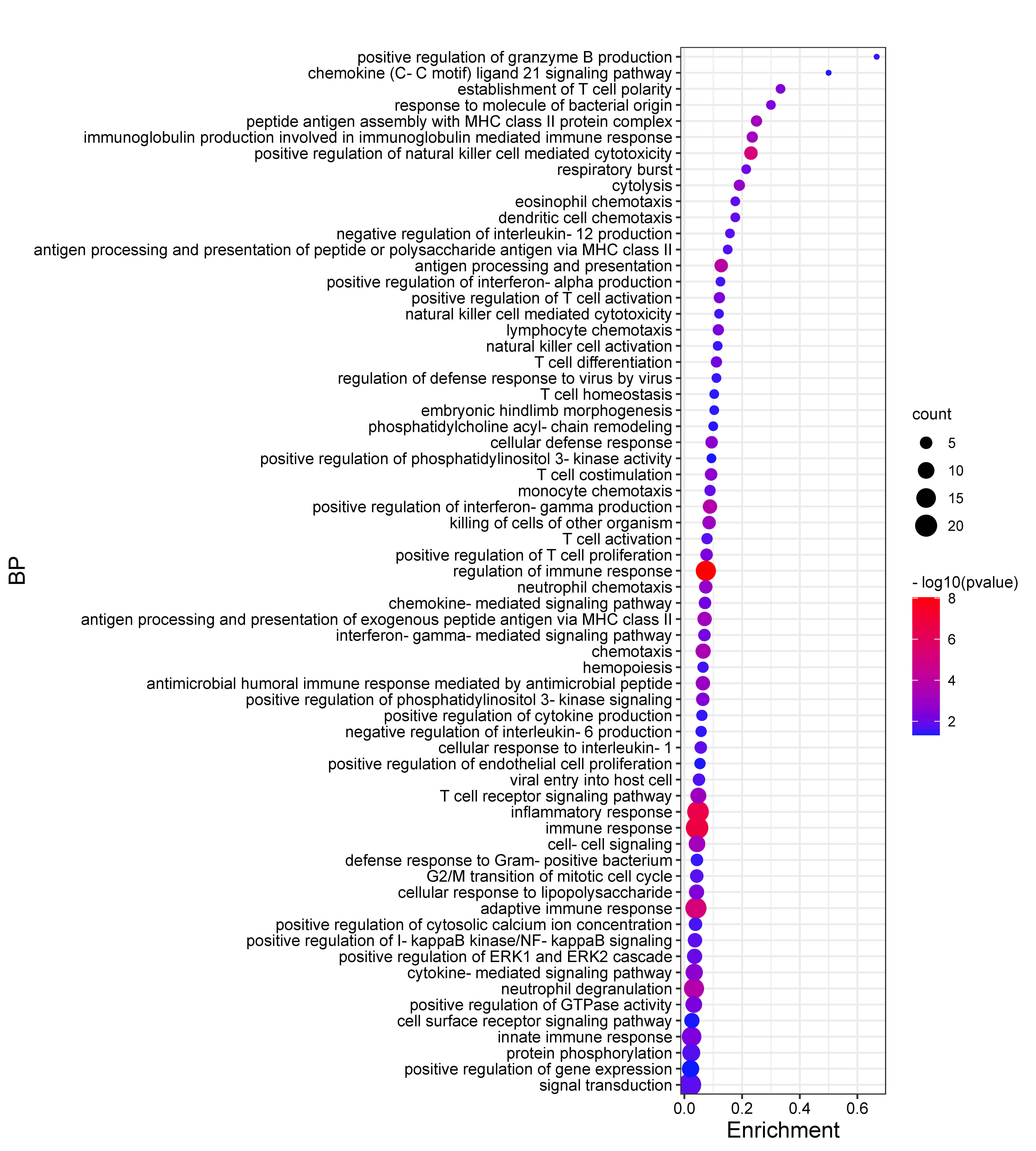

Supplement: Supplementary file 1 [file DataSheet3.ZIP › datasheet of Figure 3/TCGAgokegg/1650422528.31.tiff]

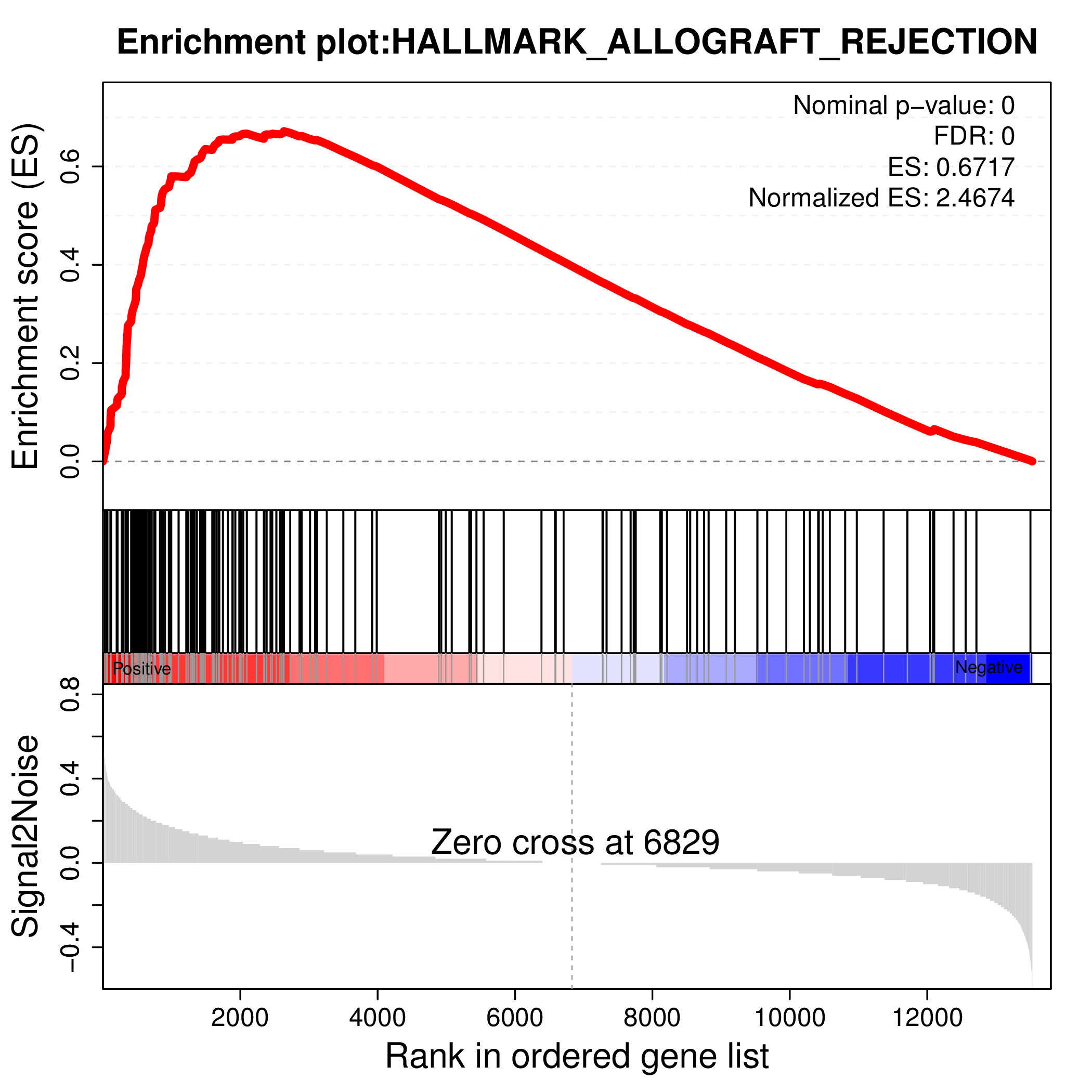

Supplement: Supplementary file 1 [file DataSheet3.ZIP › datasheet of Figure 3/TCGAgsea/HALLMARK_ALLOGRAFT_REJECTION.enplot.png]

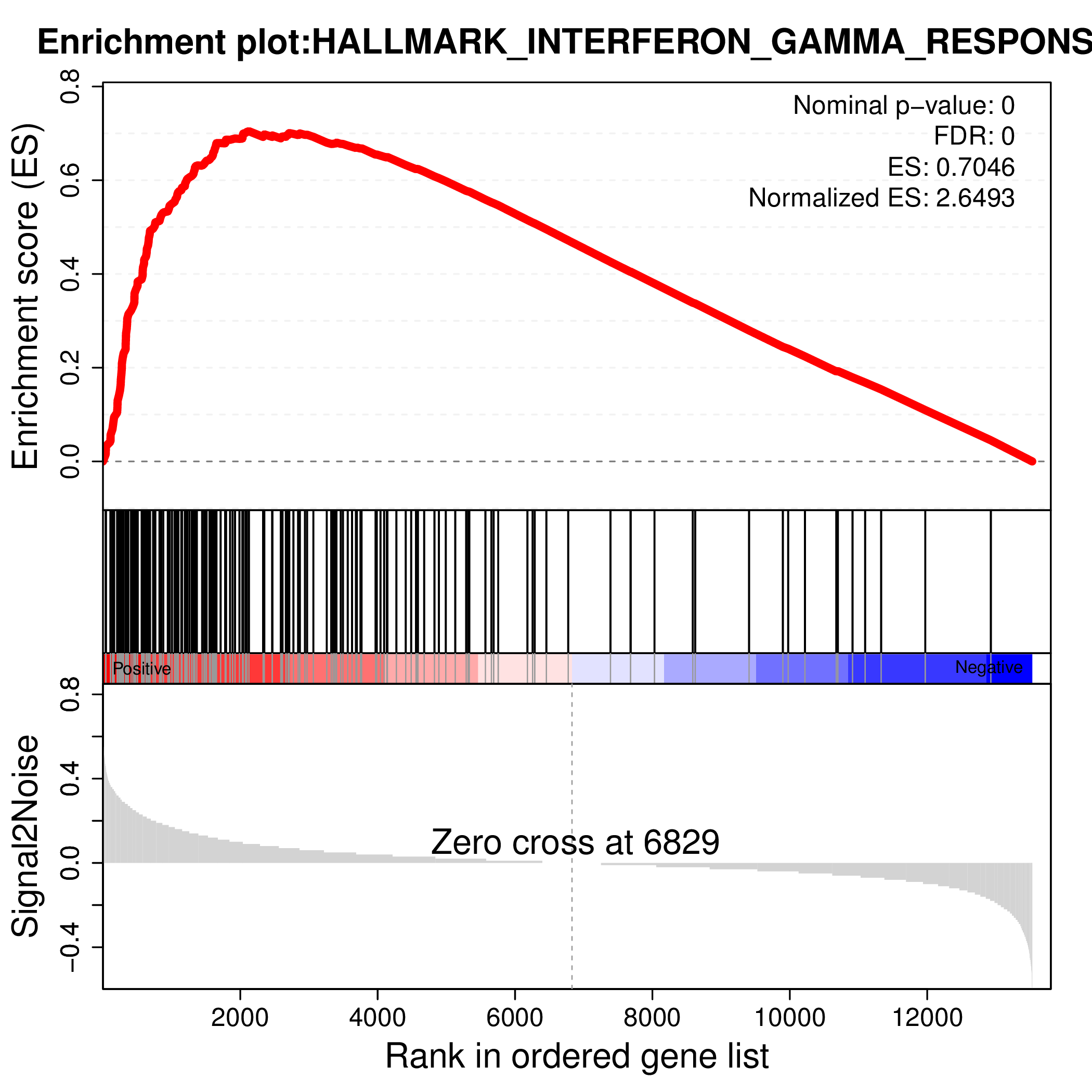

Supplement: Supplementary file 1 [file DataSheet3.ZIP › datasheet of Figure 3/TCGAgsea/HALLMARK_INTERFERON_GAMMA_RESPONSE.enplot.png]

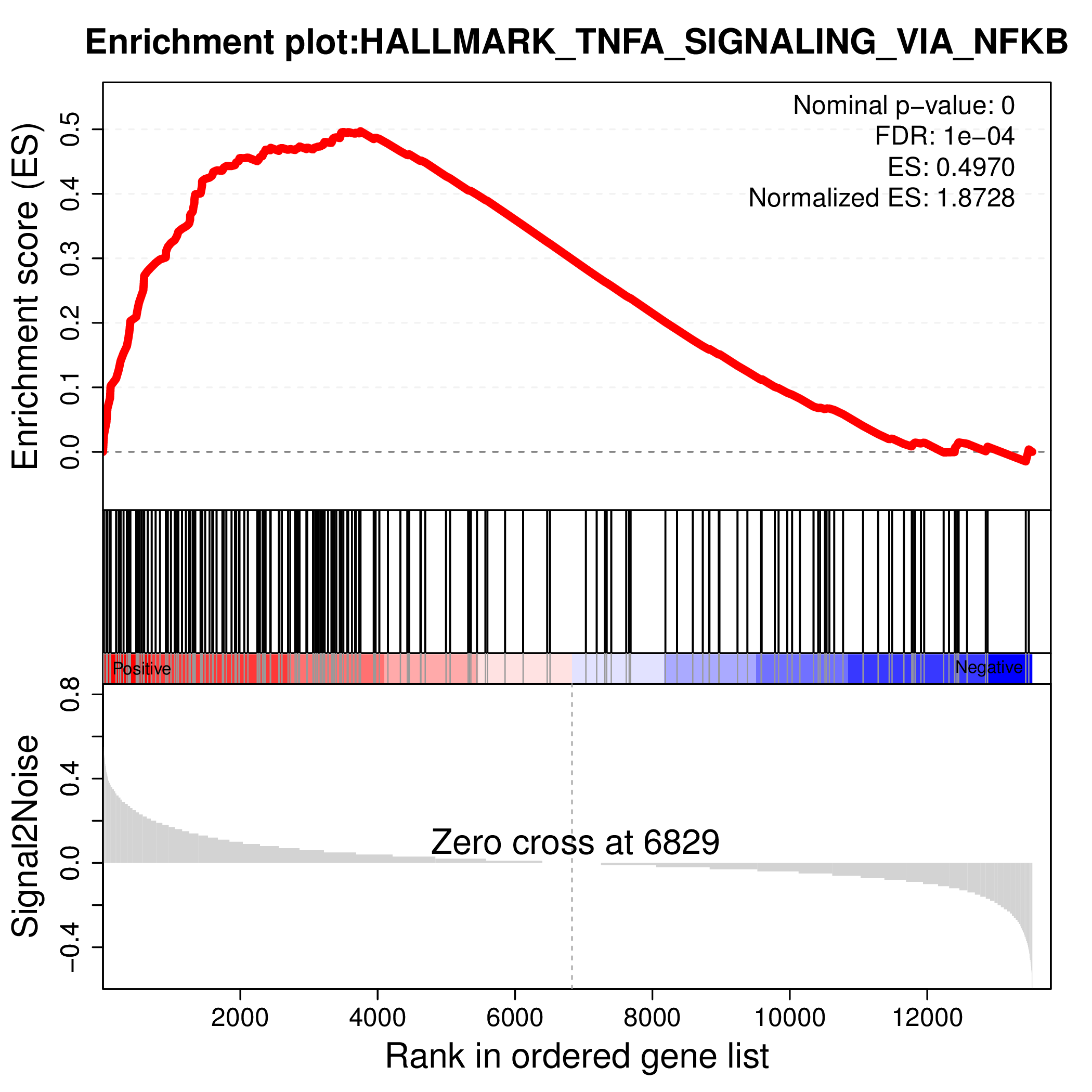

Supplement: Supplementary file 1 [file DataSheet3.ZIP › datasheet of Figure 3/TCGAgsea/HALLMARK_TNFA_SIGNALING_VIA_NFKB.enplot.png]

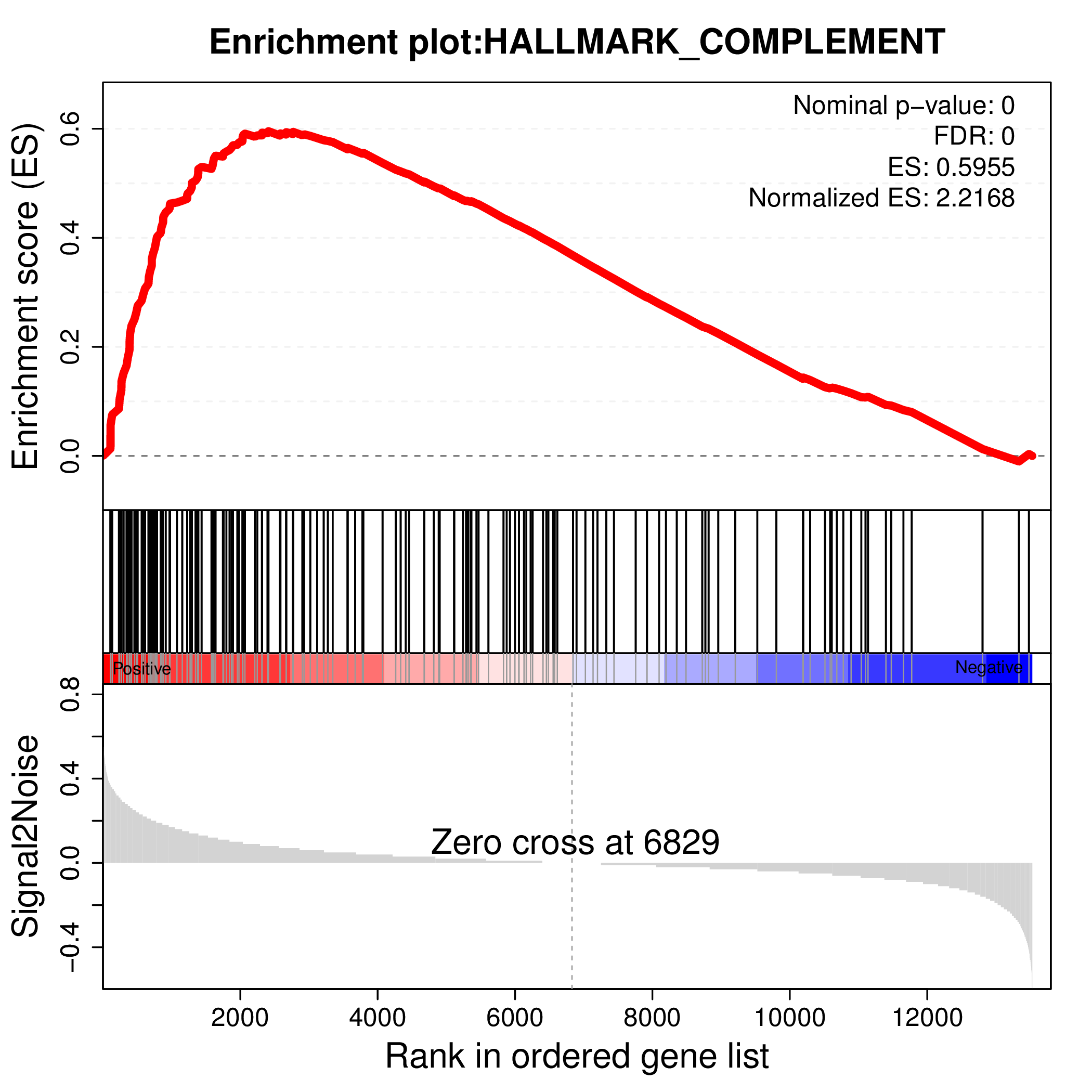

Supplement: Supplementary file 1 [file DataSheet3.ZIP › datasheet of Figure 3/TCGAgsea/HALLMARK_COMPLEMENT.enplot.png]

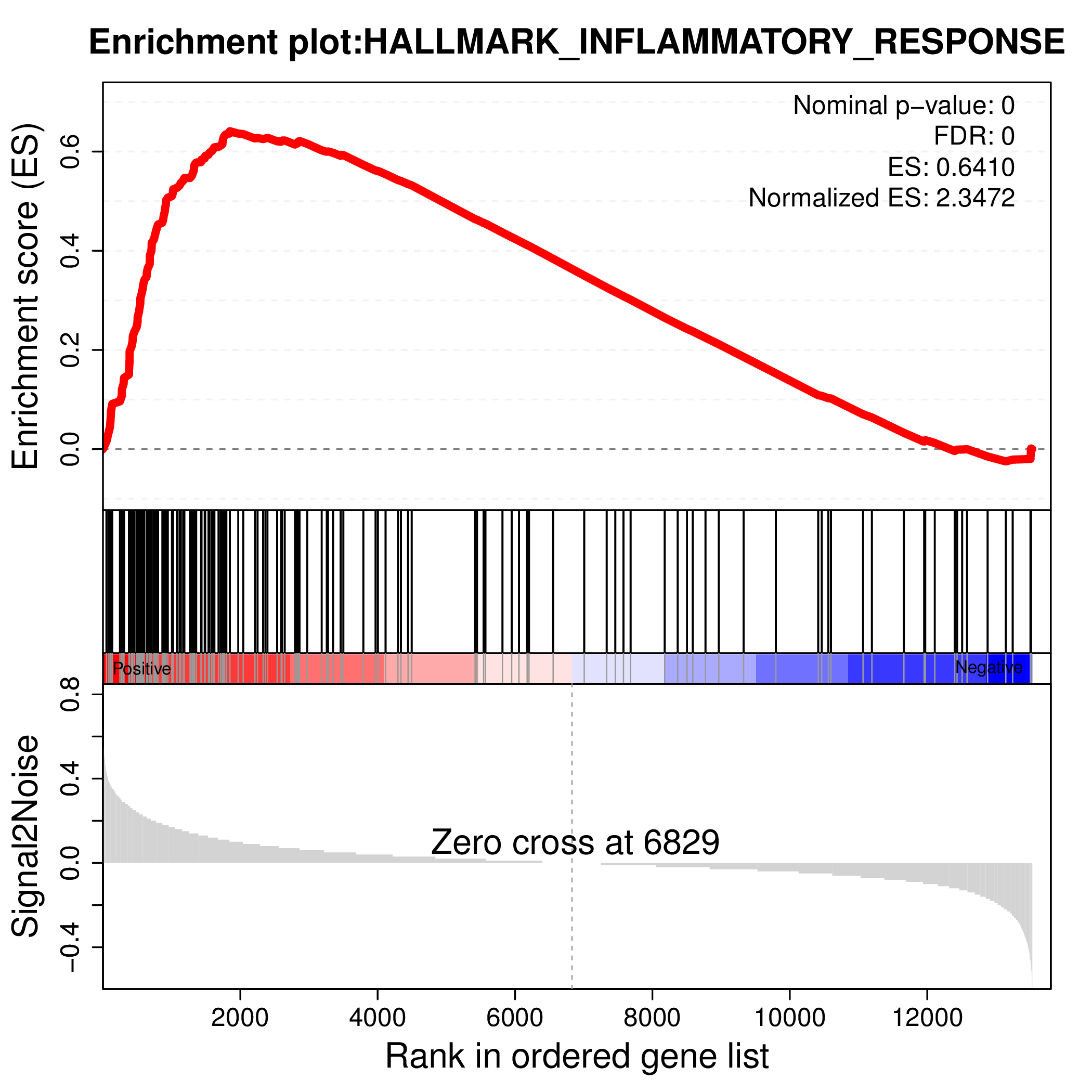

Supplement: Supplementary file 1 [file DataSheet3.ZIP › datasheet of Figure 3/TCGAgsea/HALLMARK_INFLAMMATORY_RESPONSE.enplot.png]

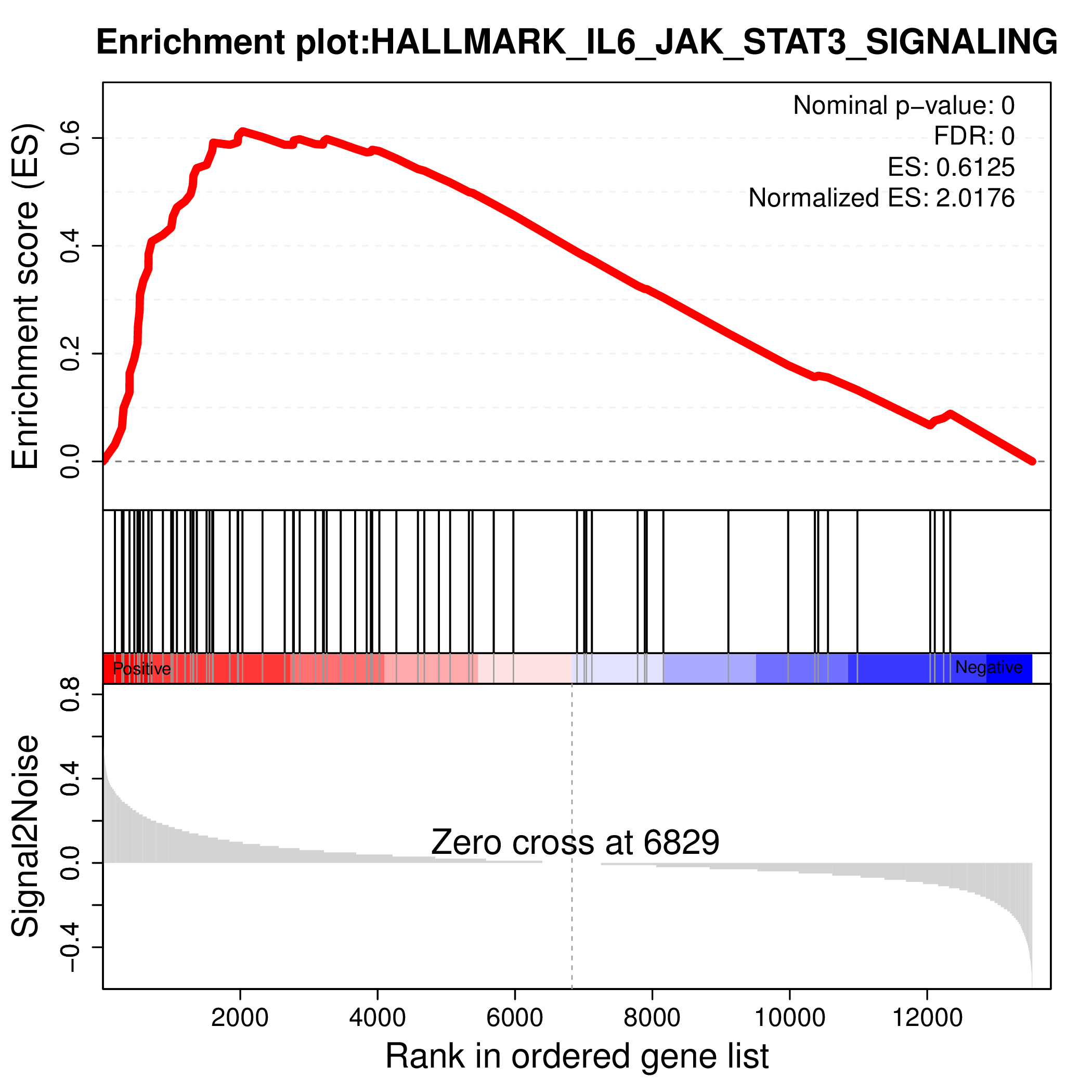

Supplement: Supplementary file 1 [file DataSheet3.ZIP › datasheet of Figure 3/TCGAgsea/HALLMARK_IL6_JAK_STAT3_SIGNALING.enplot.png]

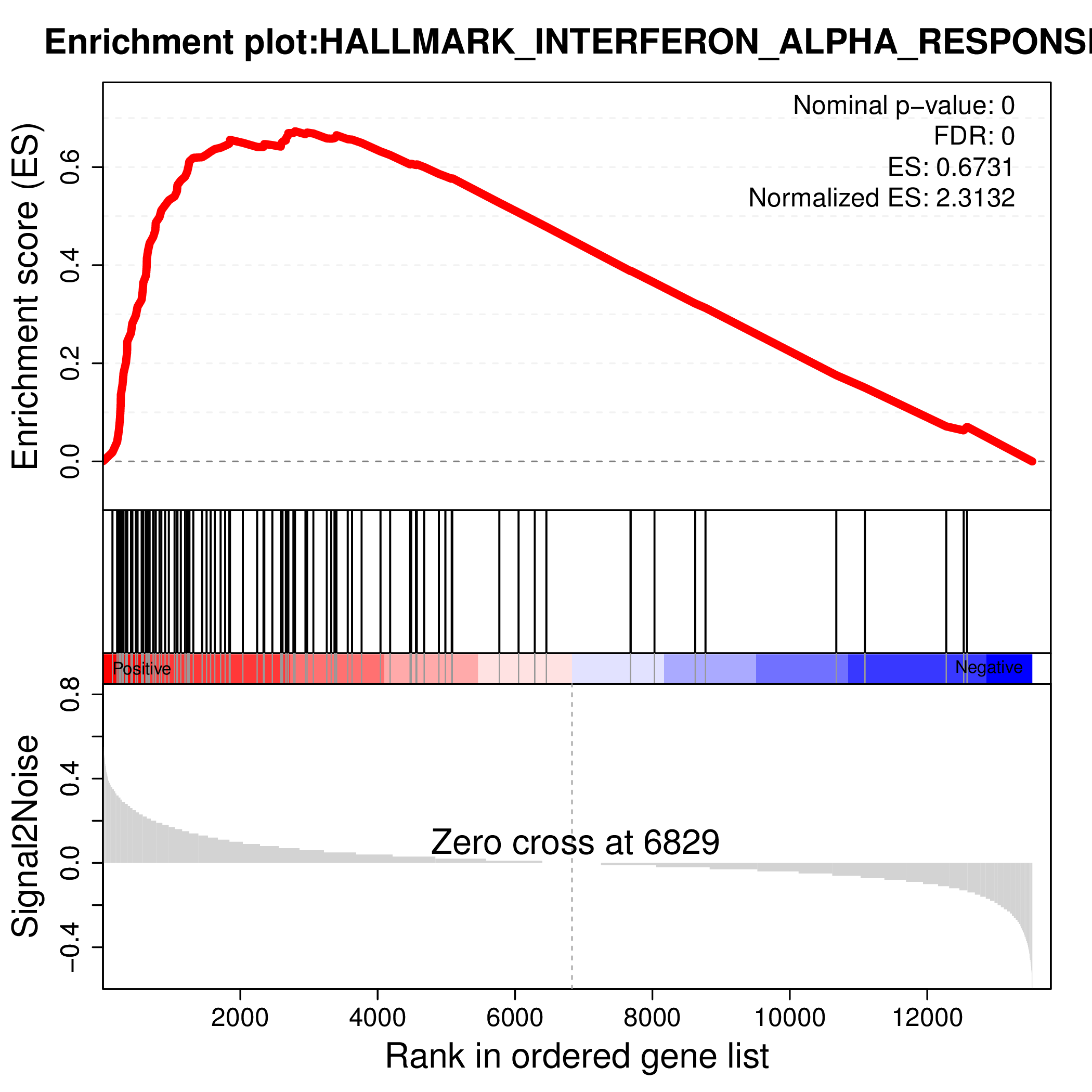

Supplement: Supplementary file 1 [file DataSheet3.ZIP › datasheet of Figure 3/TCGAgsea/HALLMARK_INTERFERON_ALPHA_RESPONSE.enplot.png]

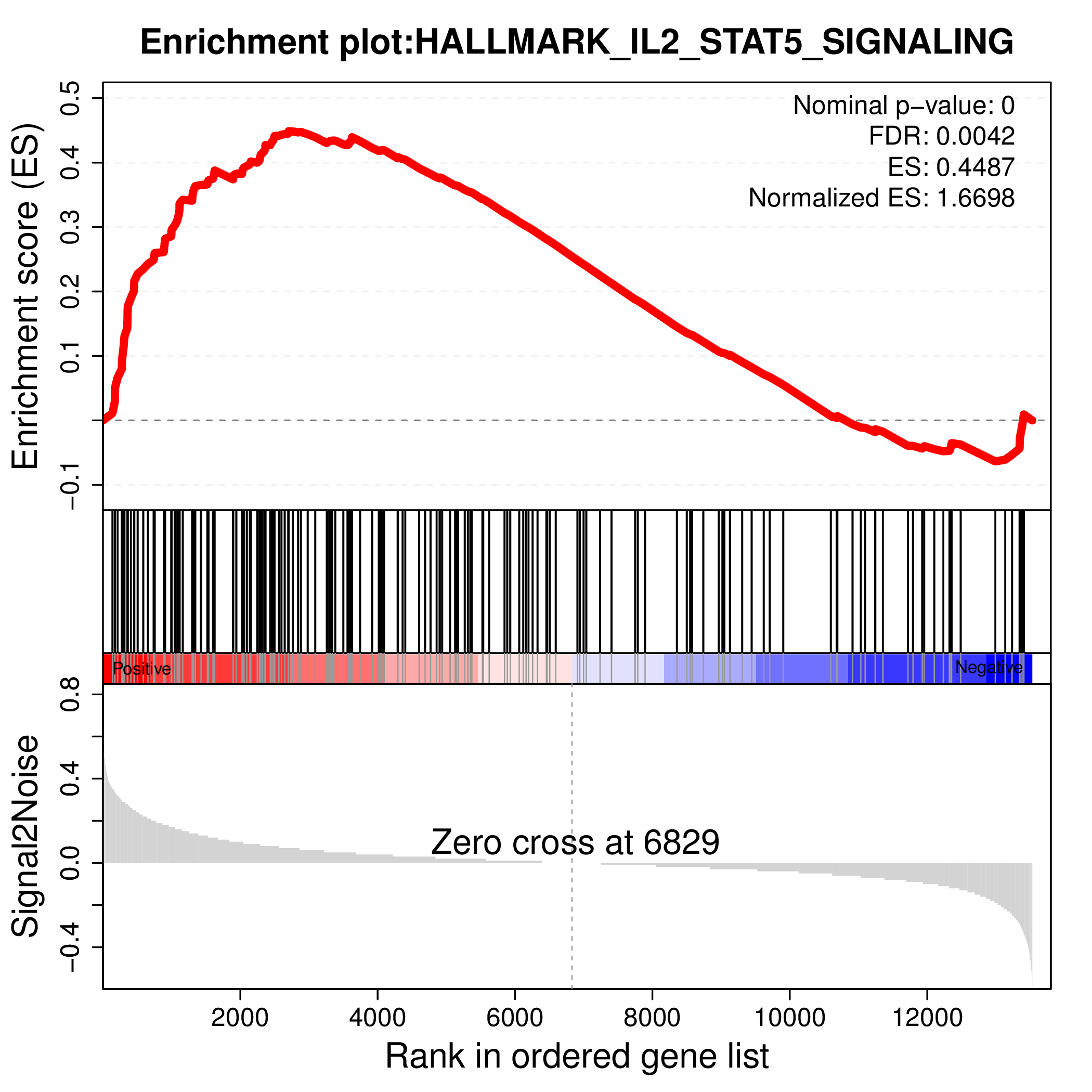

Supplement: Supplementary file 1 [file DataSheet3.ZIP › datasheet of Figure 3/TCGAgsea/HALLMARK_IL2_STAT5_SIGNALING.enplot.png]

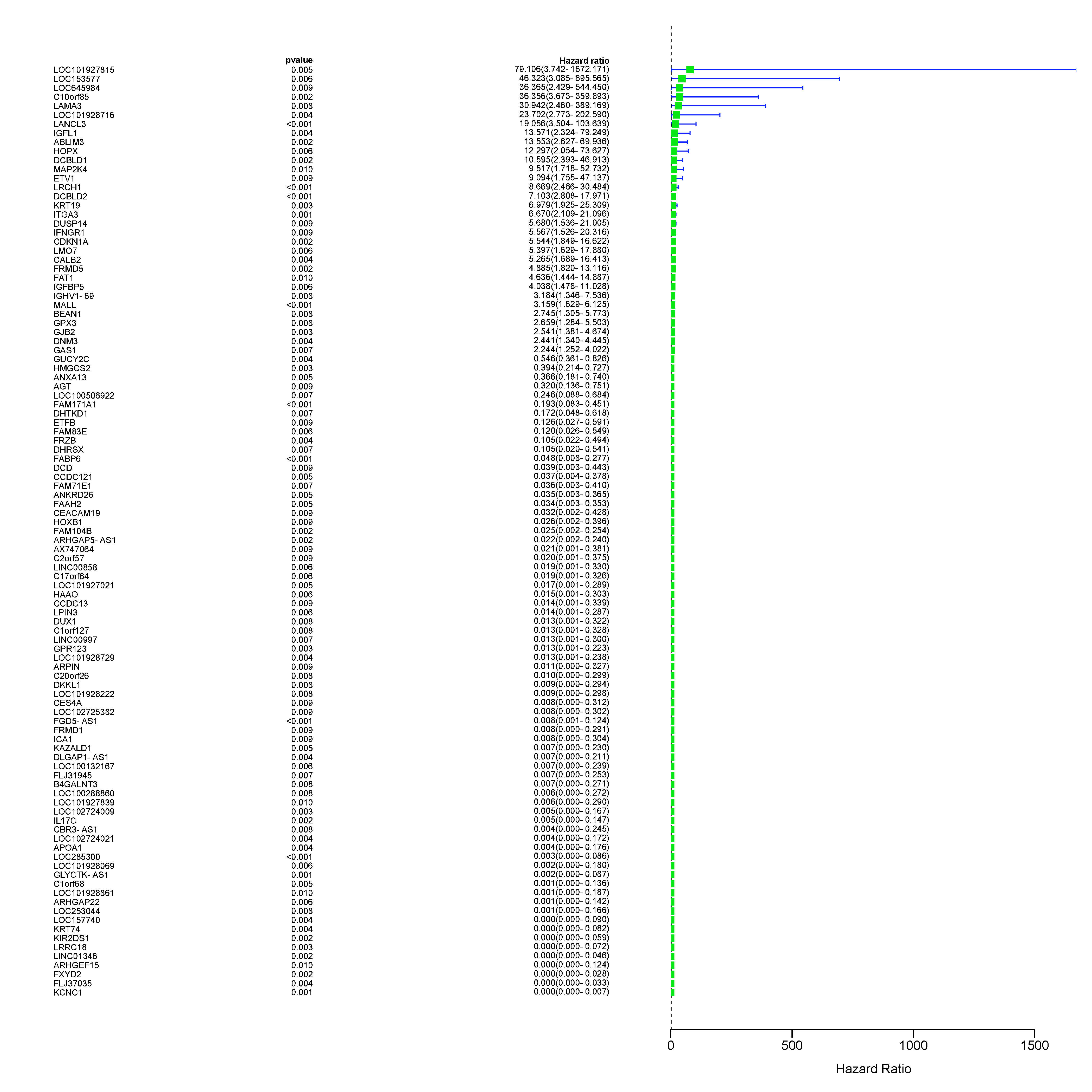

Supplement: Supplementary file 2 [file DataSheet8.ZIP › datasheet of Supplementary Figure 1/1654046865.31.png]

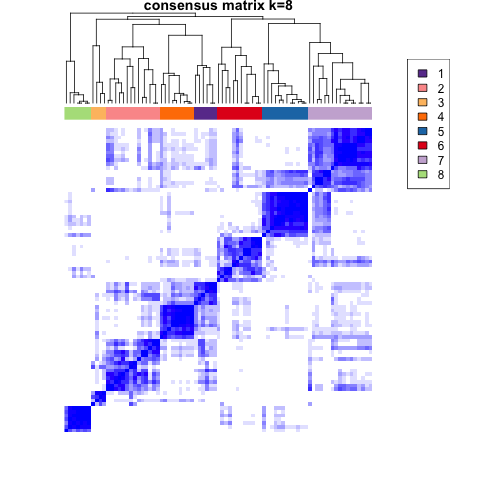

Supplement: Supplementary file 2 [file DataSheet8.ZIP › datasheet of Supplementary Figure 1/consensus008.png]

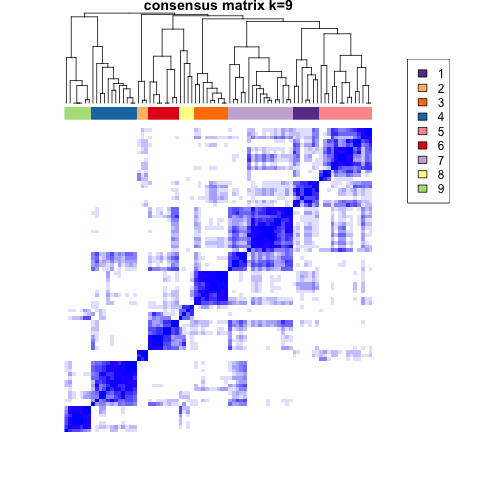

Supplement: Supplementary file 2 [file DataSheet8.ZIP › datasheet of Supplementary Figure 1/consensus009.png]

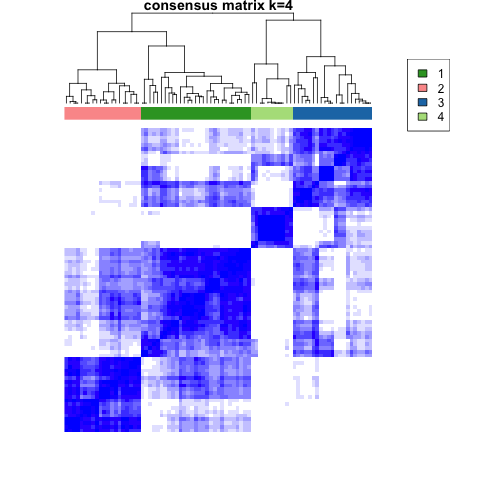

Supplement: Supplementary file 2 [file DataSheet8.ZIP › datasheet of Supplementary Figure 1/consensus004.png]

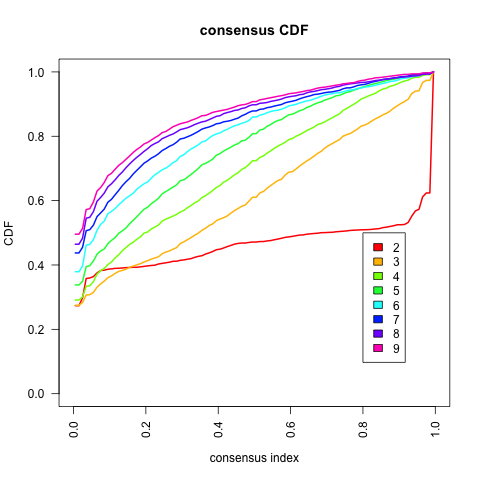

Supplement: Supplementary file 2 [file DataSheet8.ZIP › datasheet of Supplementary Figure 1/consensus010.png]

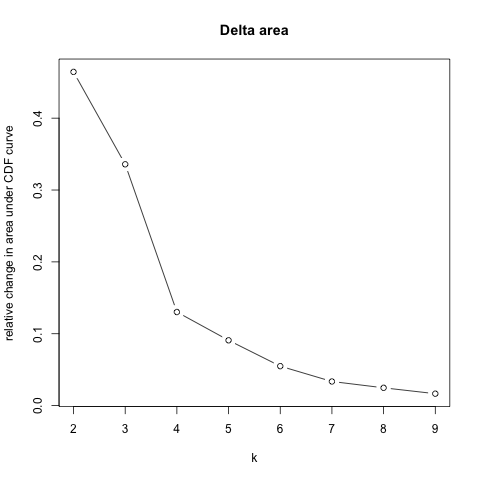

Supplement: Supplementary file 2 [file DataSheet8.ZIP › datasheet of Supplementary Figure 1/consensus011.png]

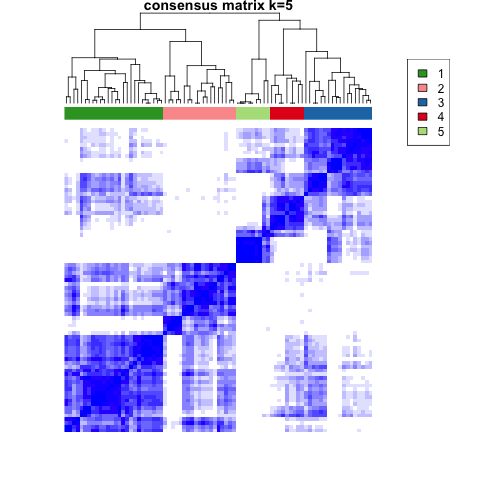

Supplement: Supplementary file 2 [file DataSheet8.ZIP › datasheet of Supplementary Figure 1/consensus005.png]

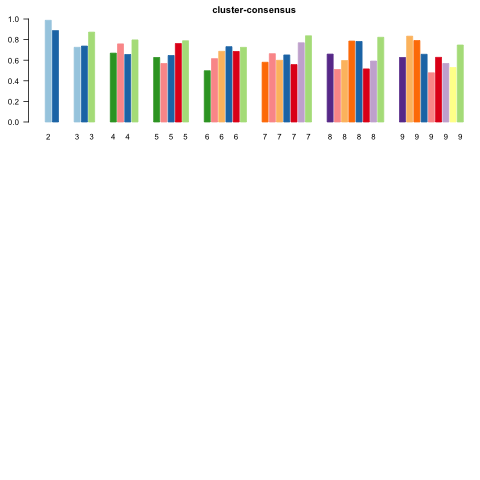

Supplement: Supplementary file 2 [file DataSheet8.ZIP › datasheet of Supplementary Figure 1/consensusScore/icl004.png]

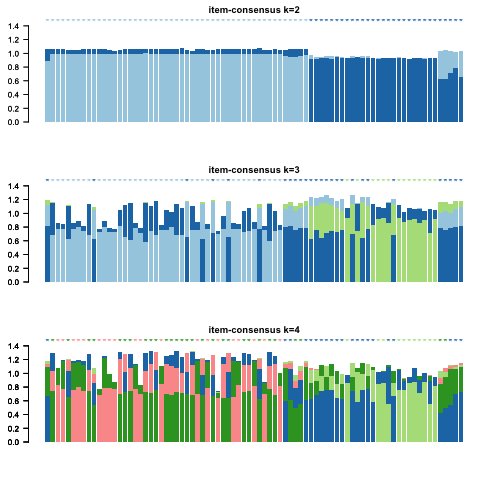

Supplement: Supplementary file 2 [file DataSheet8.ZIP › datasheet of Supplementary Figure 1/consensusScore/icl001.png]

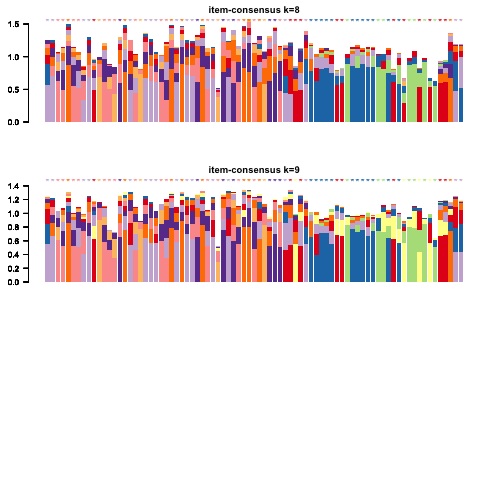

Supplement: Supplementary file 2 [file DataSheet8.ZIP › datasheet of Supplementary Figure 1/consensusScore/icl003.png]

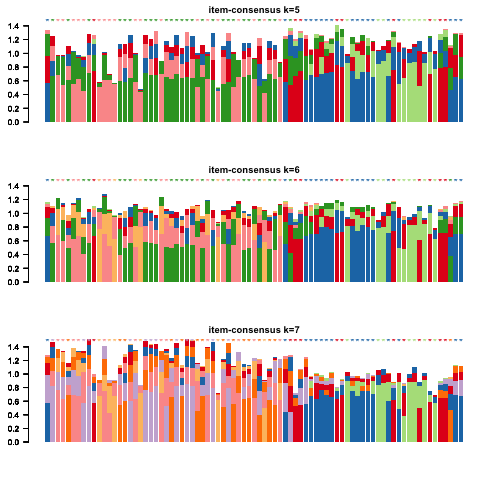

Supplement: Supplementary file 2 [file DataSheet8.ZIP › datasheet of Supplementary Figure 1/consensusScore/icl002.png]

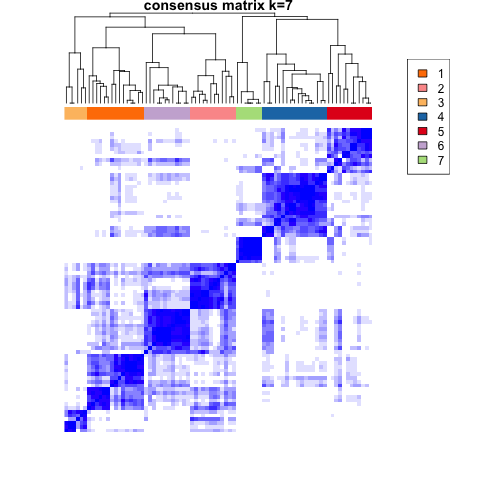

Supplement: Supplementary file 2 [file DataSheet8.ZIP › datasheet of Supplementary Figure 1/consensus007.png]

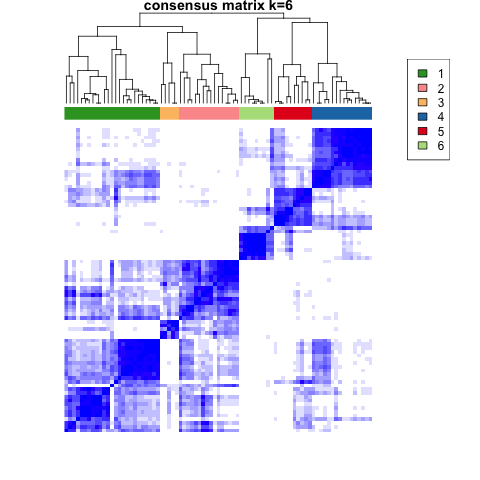

Supplement: Supplementary file 2 [file DataSheet8.ZIP › datasheet of Supplementary Figure 1/consensus006.png]

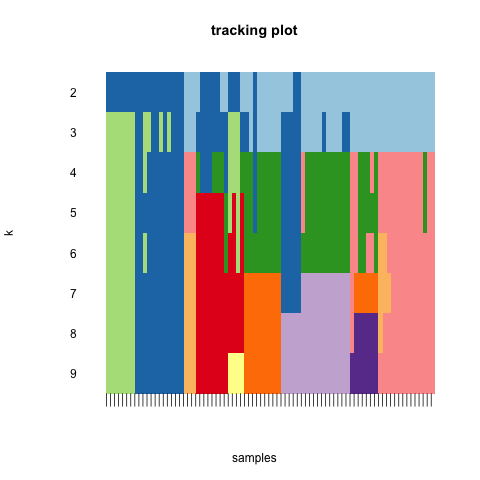

Supplement: Supplementary file 2 [file DataSheet8.ZIP › datasheet of Supplementary Figure 1/consensus012.png]

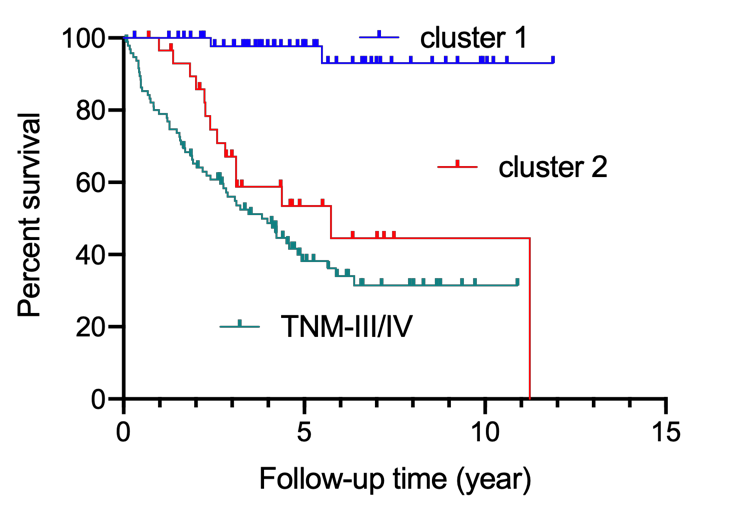

Supplement: Supplementary file 2 [file DataSheet8.ZIP › datasheet of Supplementary Figure 1/17536OSall.tiff]

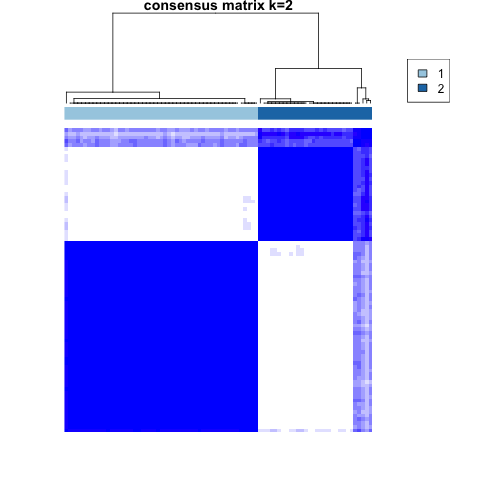

Supplement: Supplementary file 2 [file DataSheet8.ZIP › datasheet of Supplementary Figure 1/consensus002.png]

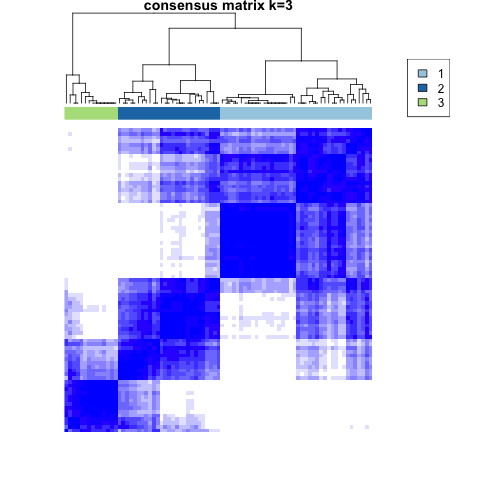

Supplement: Supplementary file 2 [file DataSheet8.ZIP › datasheet of Supplementary Figure 1/consensus003.png]

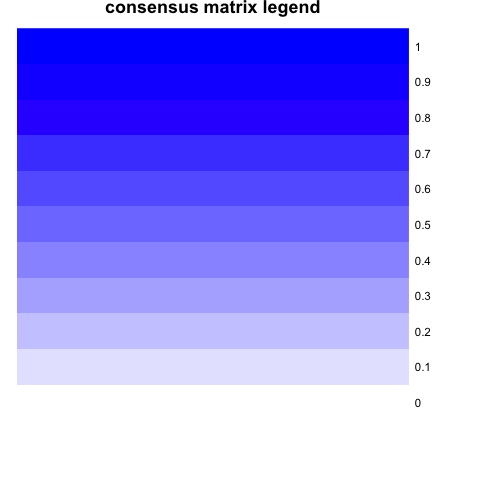

Supplement: Supplementary file 2 [file DataSheet8.ZIP › datasheet of Supplementary Figure 1/consensus001.png]

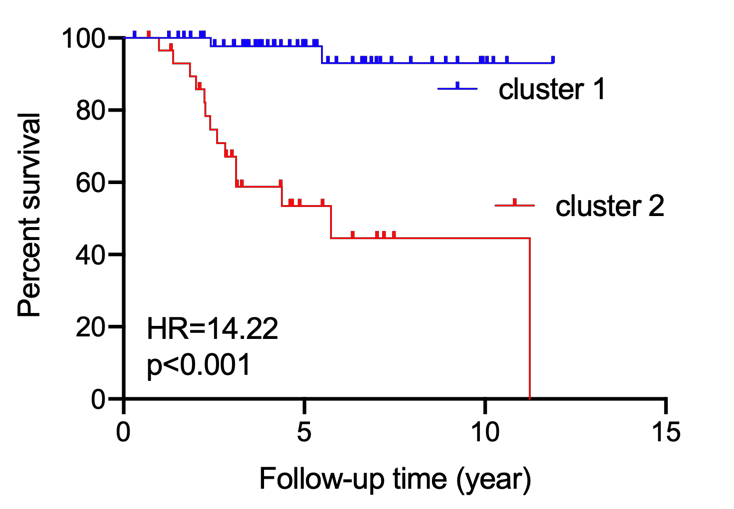

Supplement: Supplementary file 2 [file DataSheet8.ZIP › datasheet of Supplementary Figure 1/prognosis.tiff]

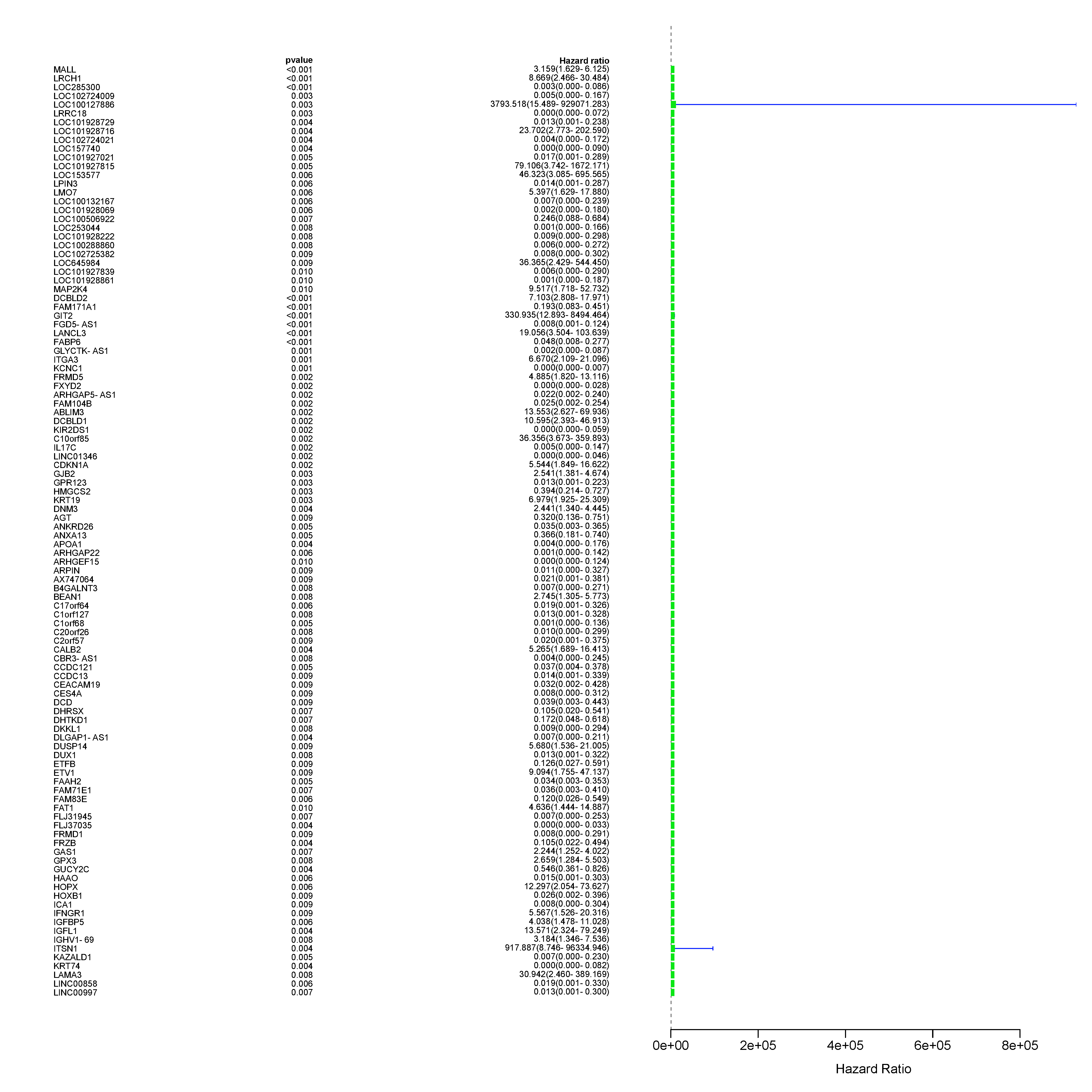

Supplement: Supplementary file 2 [file DataSheet8.ZIP › datasheet of Supplementary Figure 1/1654046759.94.png]

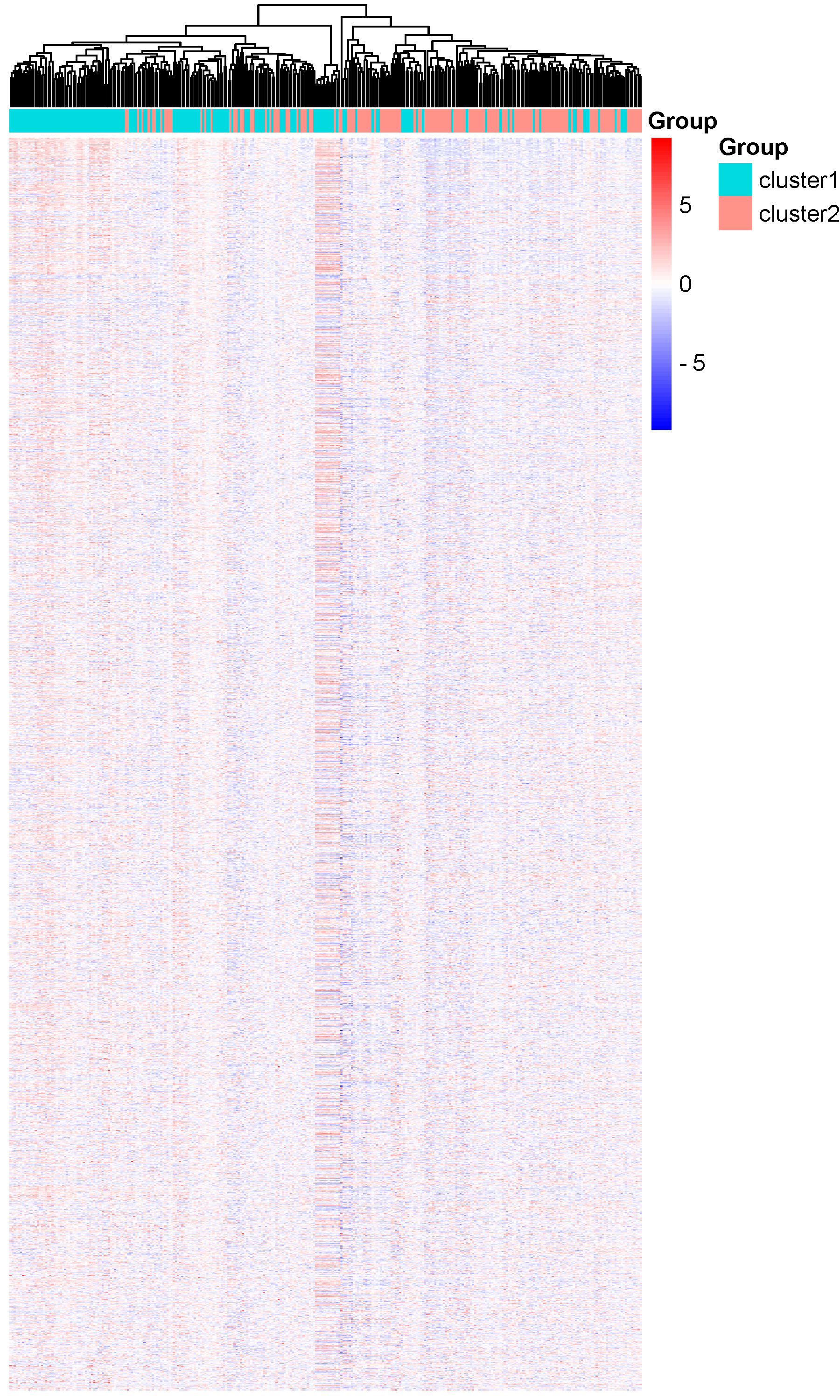

Supplement: Supplementary file 3 [file DataSheet9.ZIP › datasheet of Supplementary Figure 2/GSE39582gokegg/1654063598.02.png]

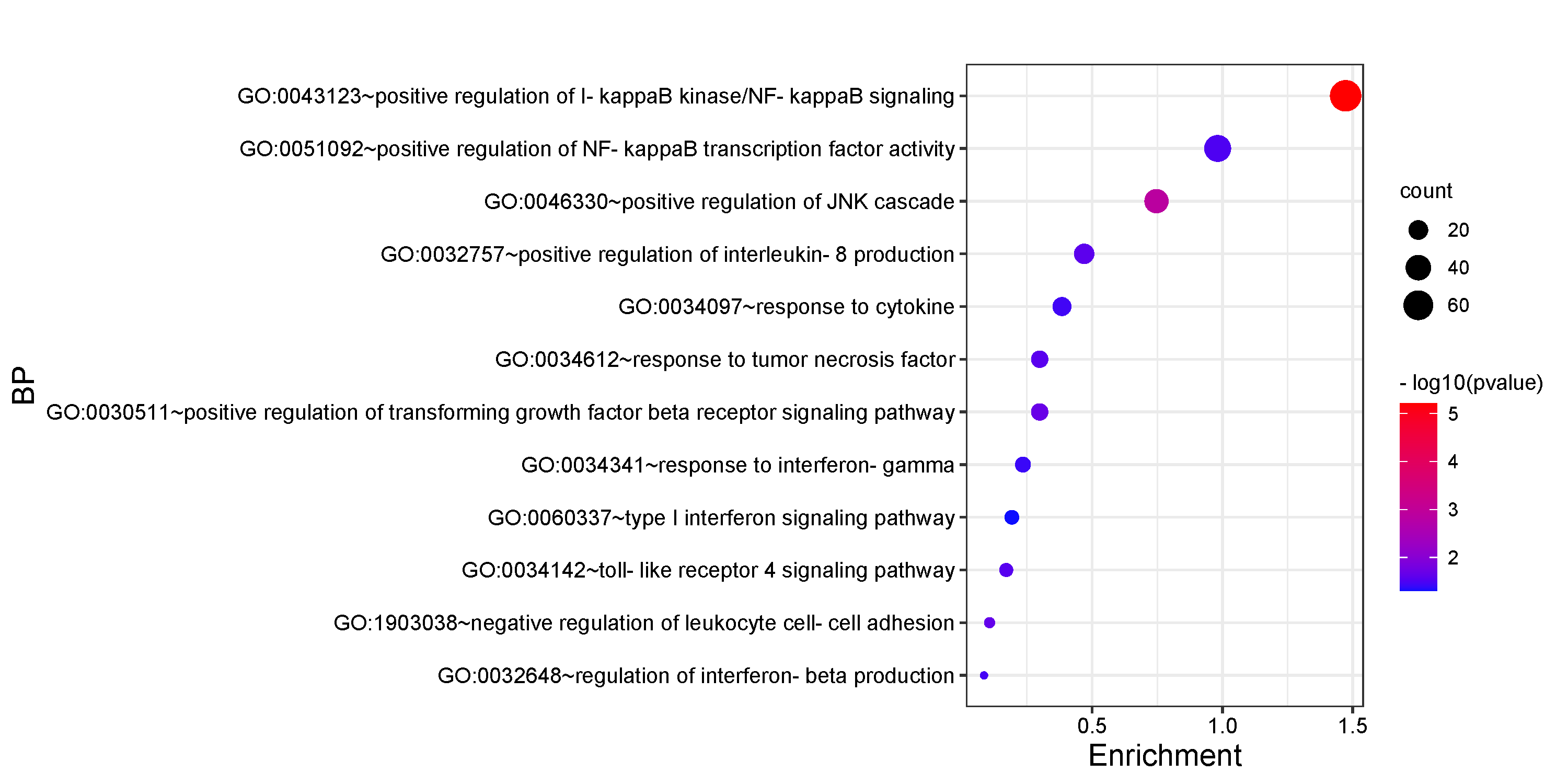

Supplement: Supplementary file 3 [file DataSheet9.ZIP › datasheet of Supplementary Figure 2/GSE39582gokegg/1654062512.6.png]

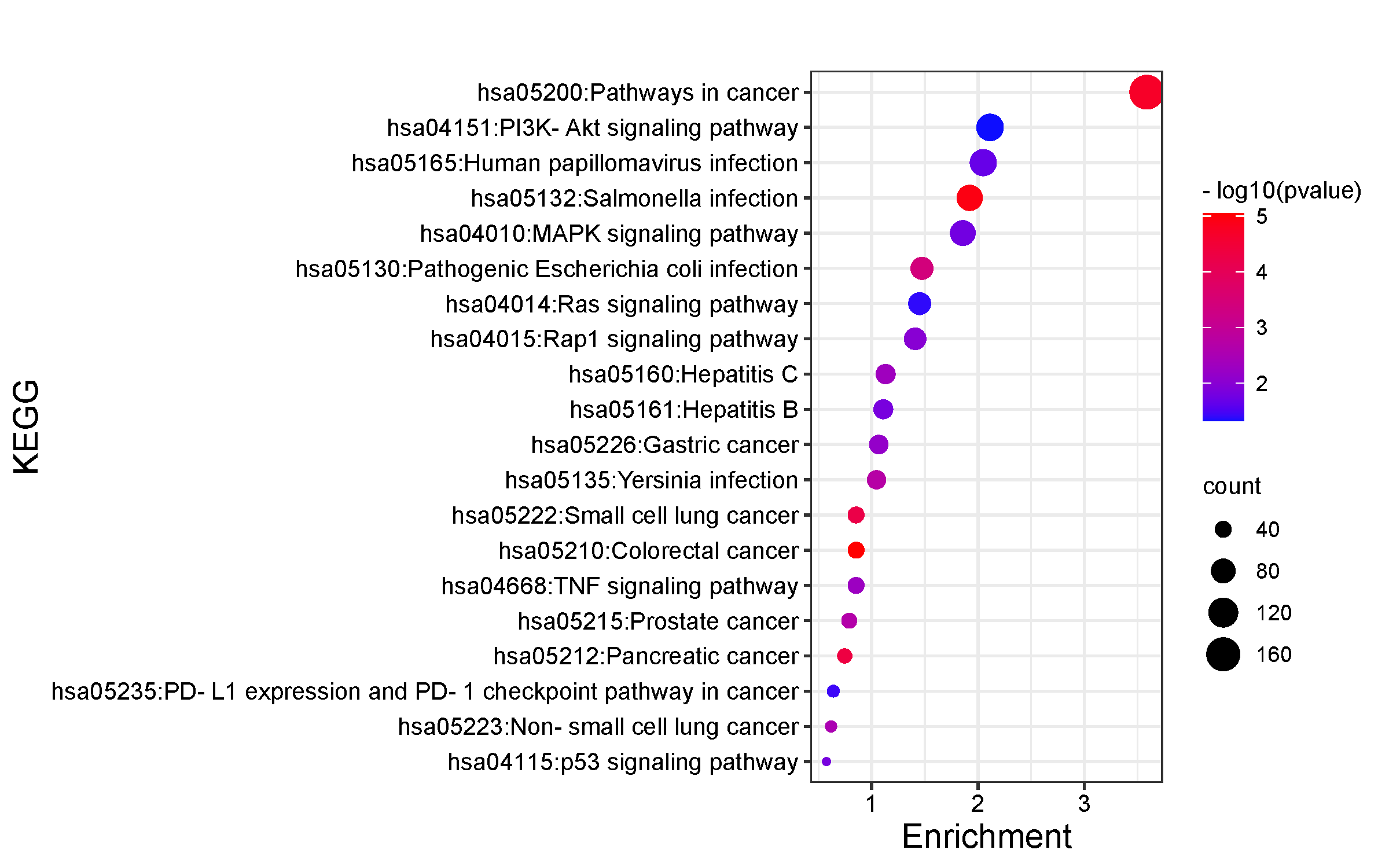

Supplement: Supplementary file 3 [file DataSheet9.ZIP › datasheet of Supplementary Figure 2/GSE39582gokegg/1654062702.1.png]

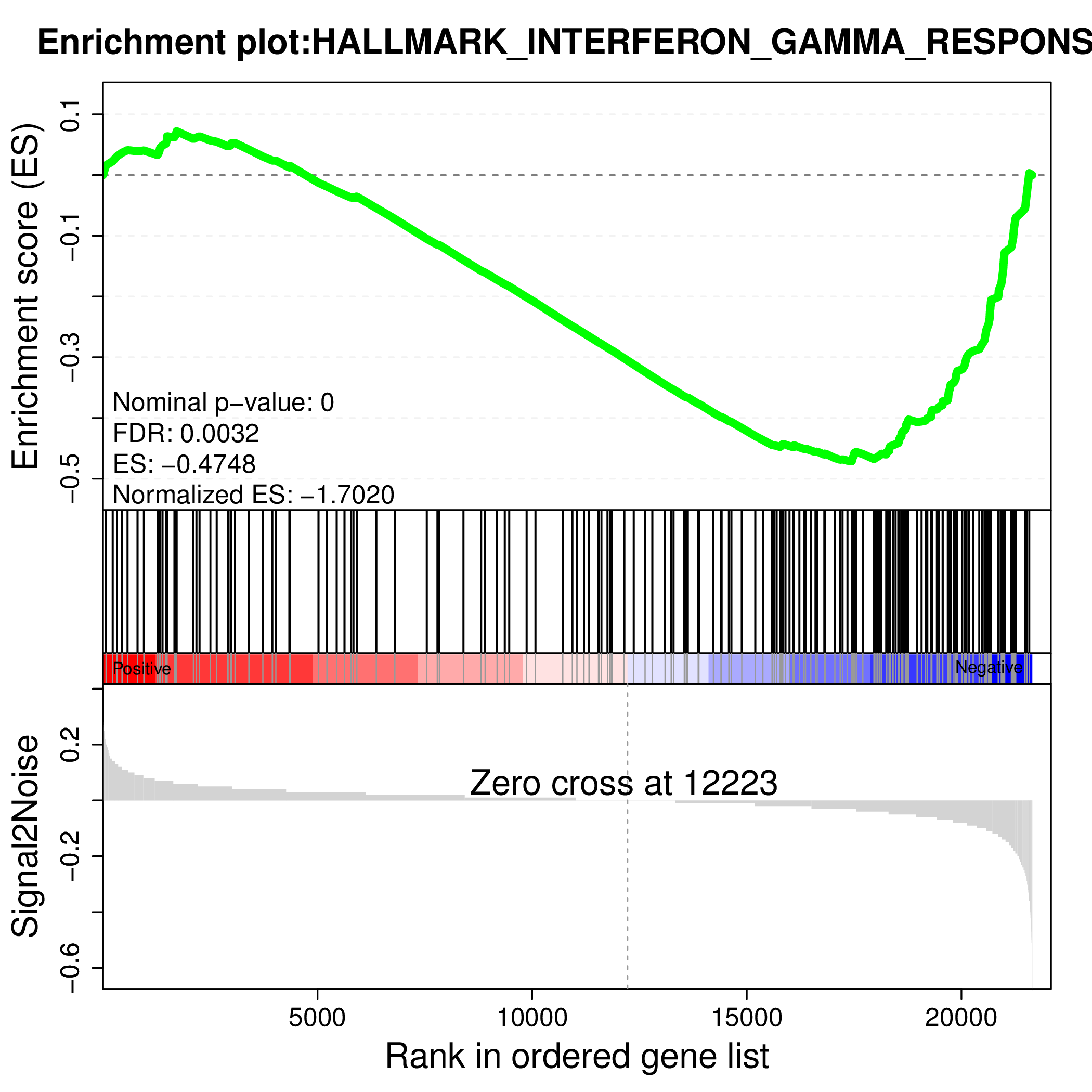

Supplement: Supplementary file 3 [file DataSheet9.ZIP › datasheet of Supplementary Figure 2/GSE39582gsea/HALLMARK_INTERFERON_GAMMA_RESPONSE.enplot.png]

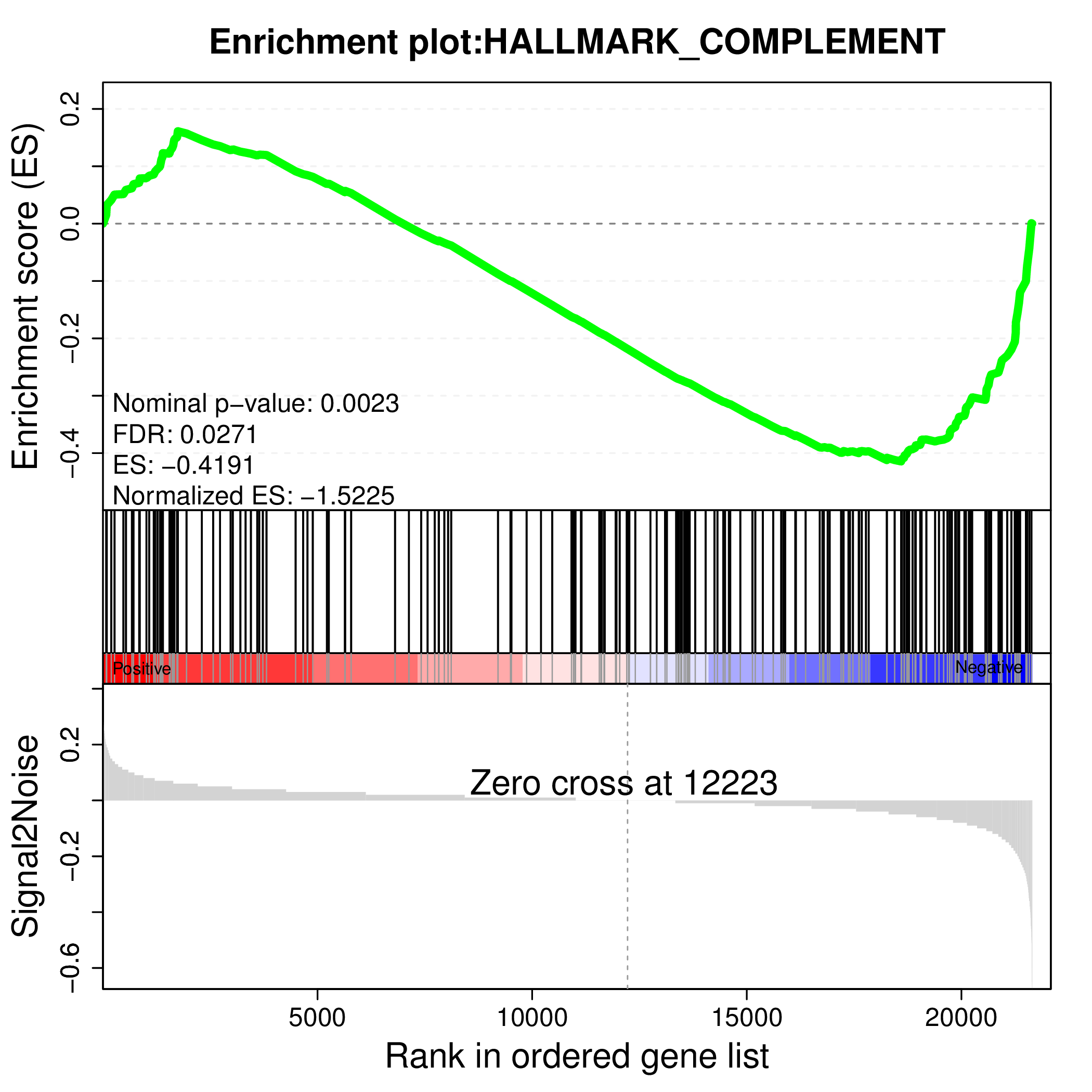

Supplement: Supplementary file 3 [file DataSheet9.ZIP › datasheet of Supplementary Figure 2/GSE39582gsea/HALLMARK_COMPLEMENT.enplot.png]

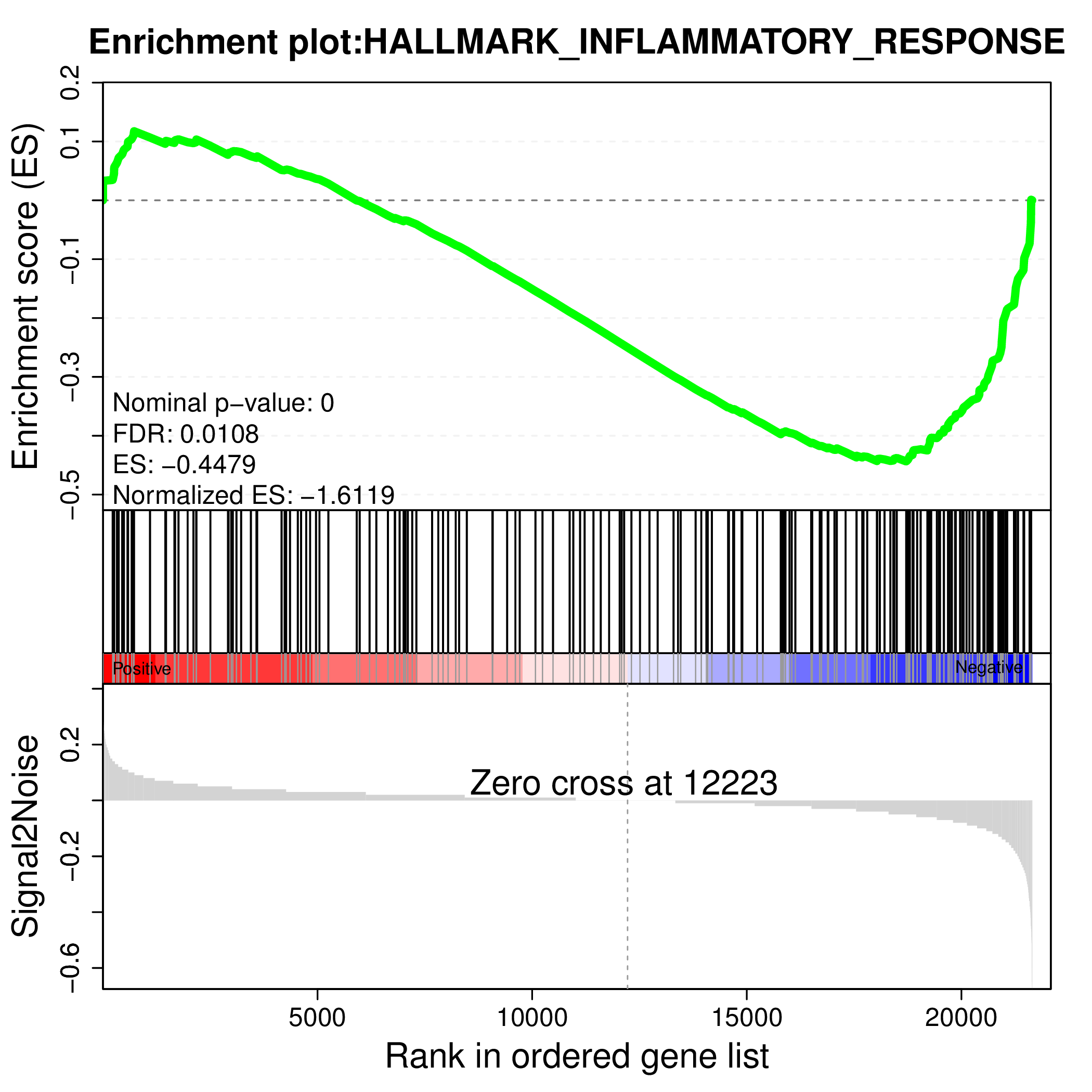

Supplement: Supplementary file 3 [file DataSheet9.ZIP › datasheet of Supplementary Figure 2/GSE39582gsea/HALLMARK_INFLAMMATORY_RESPONSE.enplot.png]

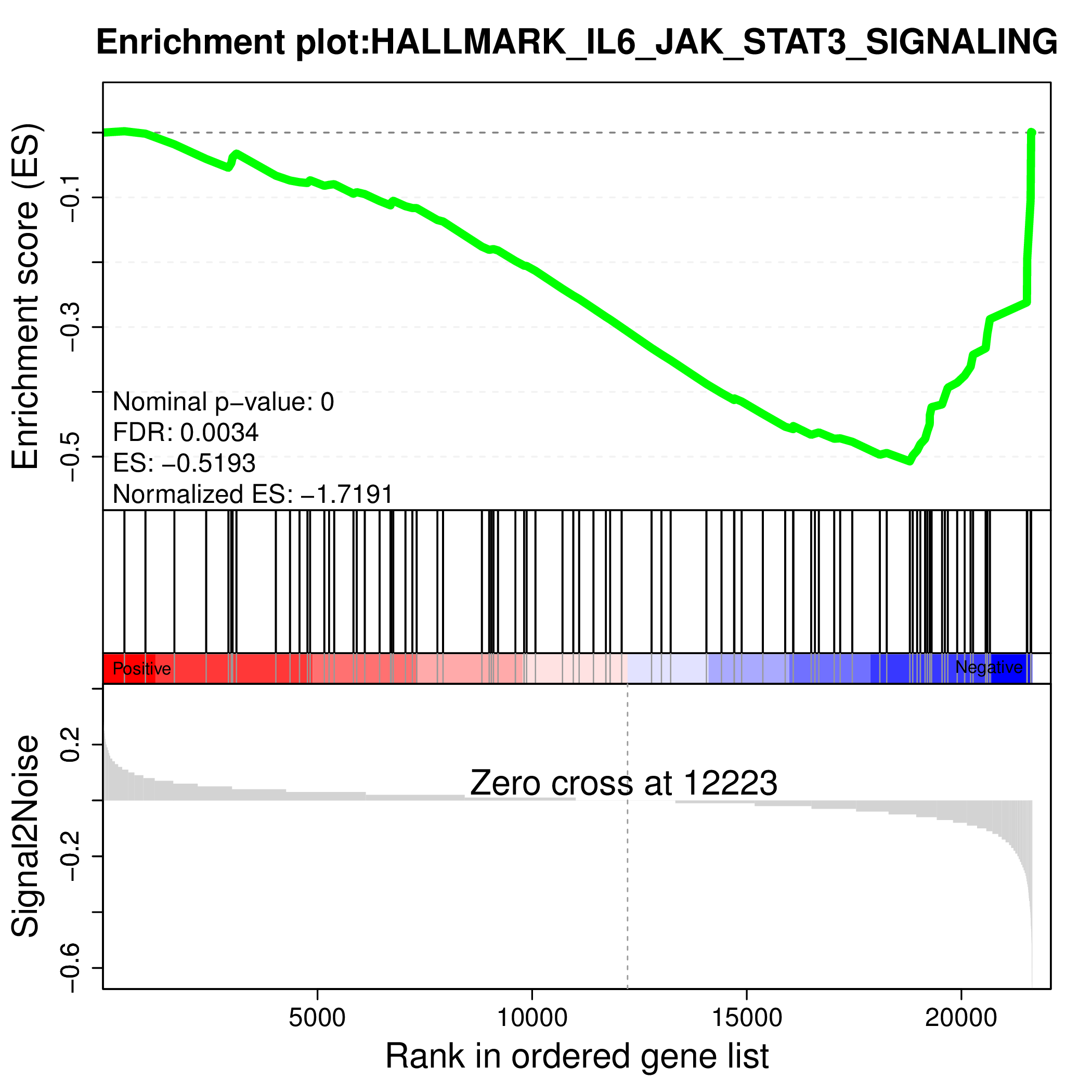

Supplement: Supplementary file 3 [file DataSheet9.ZIP › datasheet of Supplementary Figure 2/GSE39582gsea/HALLMARK_IL6_JAK_STAT3_SIGNALING.enplot.png]

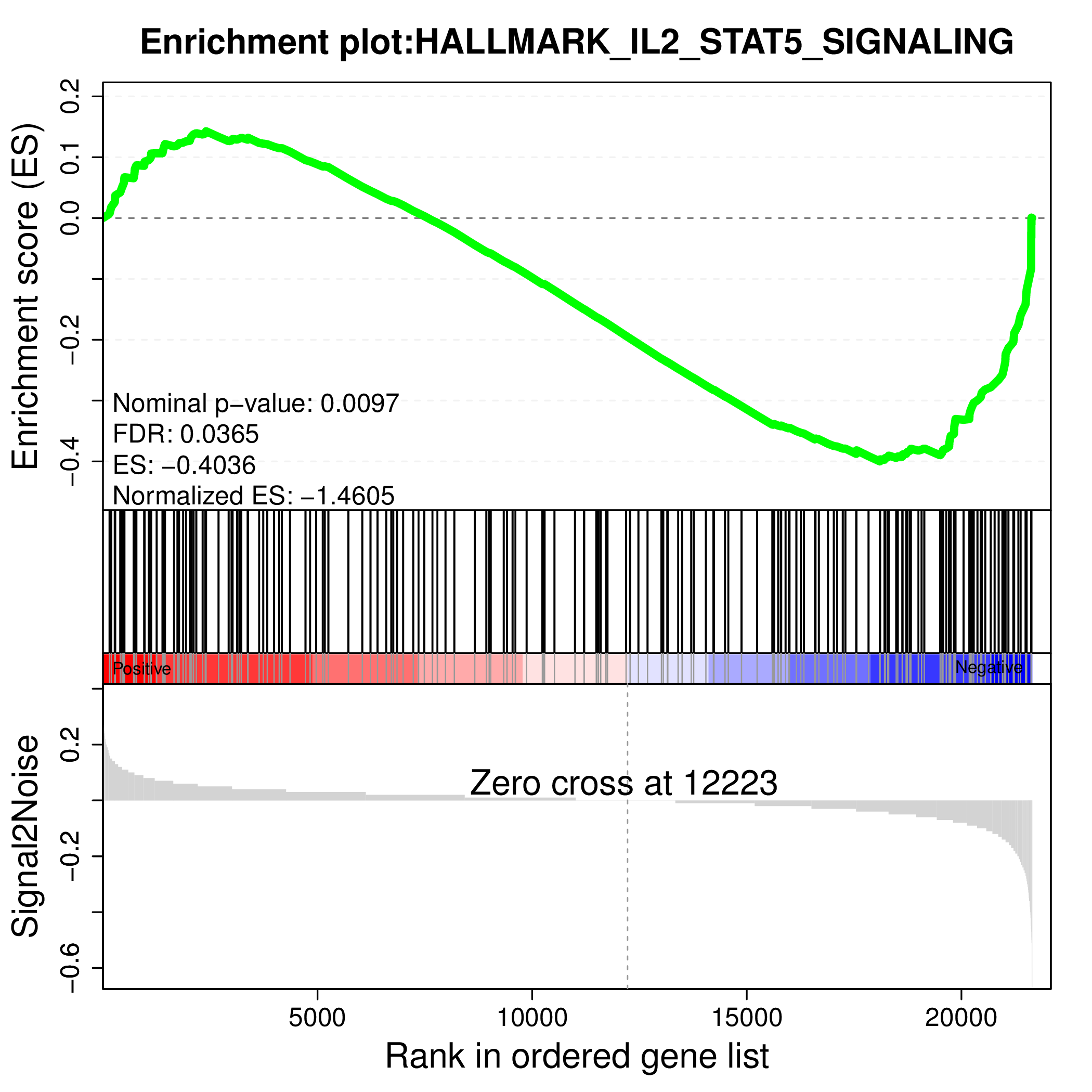

Supplement: Supplementary file 3 [file DataSheet9.ZIP › datasheet of Supplementary Figure 2/GSE39582gsea/HALLMARK_IL2_STAT5_SIGNALING.enplot.png]

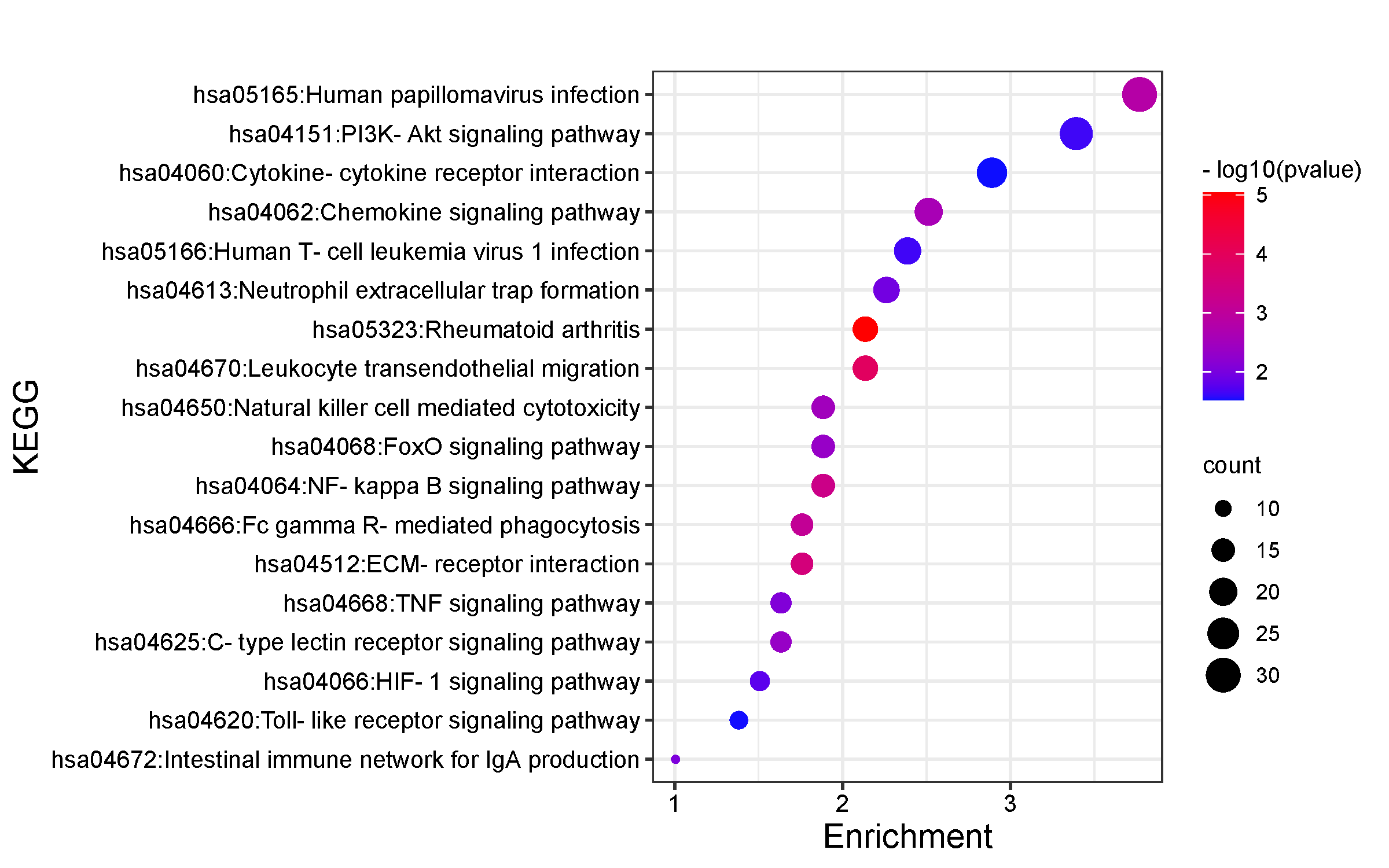

Supplement: Supplementary file 5 [file DataSheet4.ZIP › datasheet of Figure 4/GSE17536gokegg/1654060154.12.png]

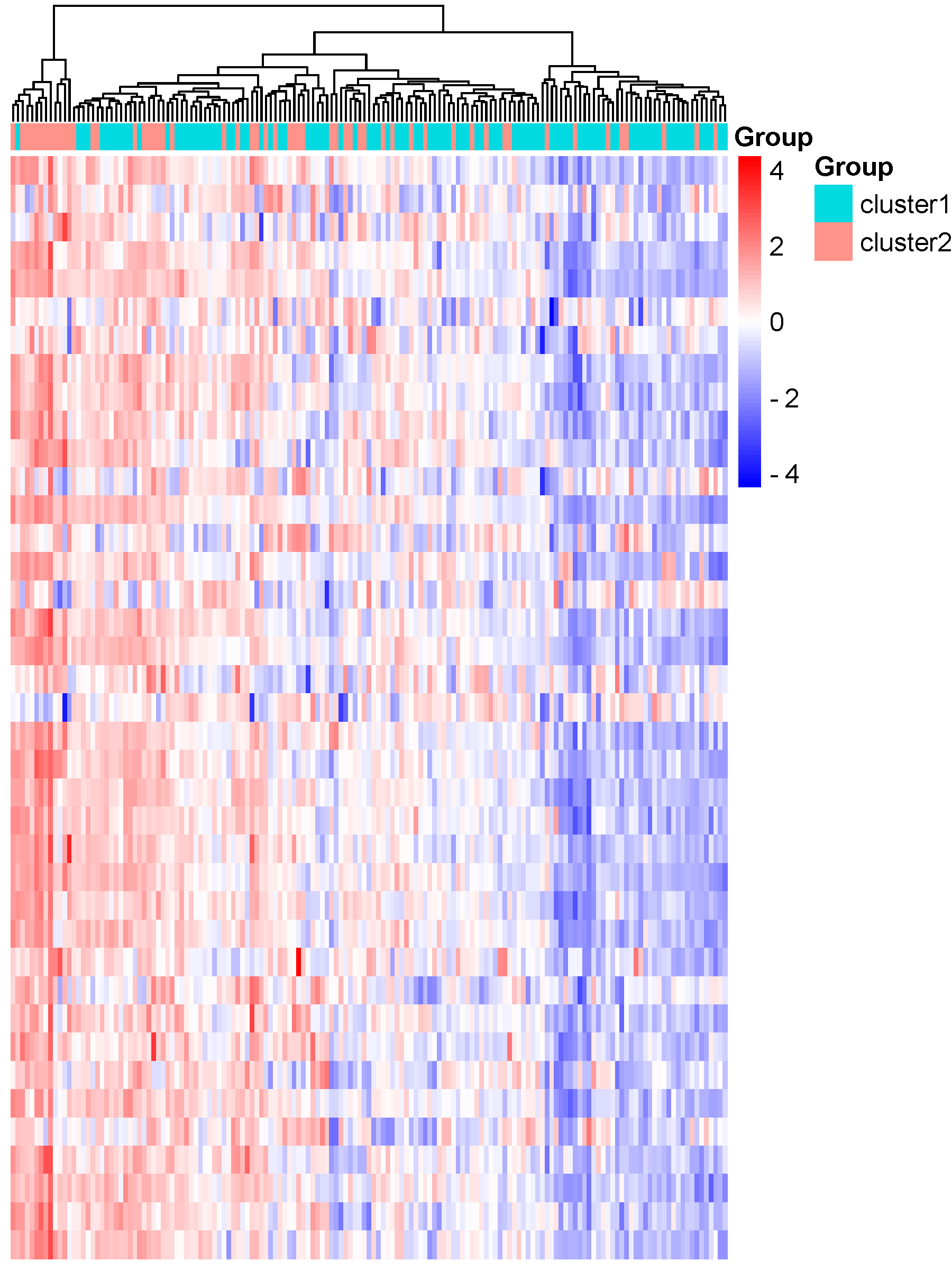

Supplement: Supplementary file 5 [file DataSheet4.ZIP › datasheet of Figure 4/GSE17536gokegg/17536.png]

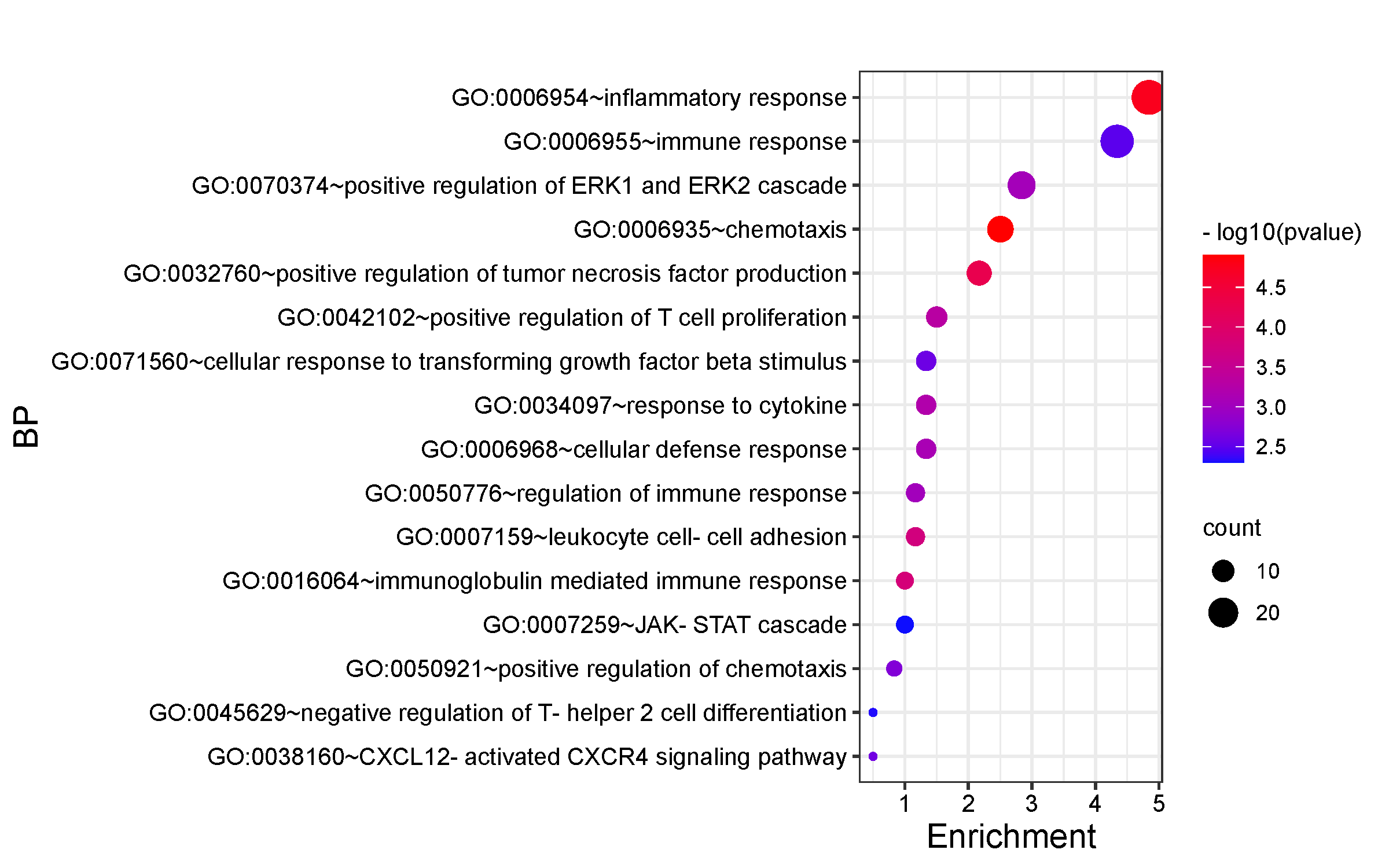

Supplement: Supplementary file 5 [file DataSheet4.ZIP › datasheet of Figure 4/GSE17536gokegg/1654060045.85.png]

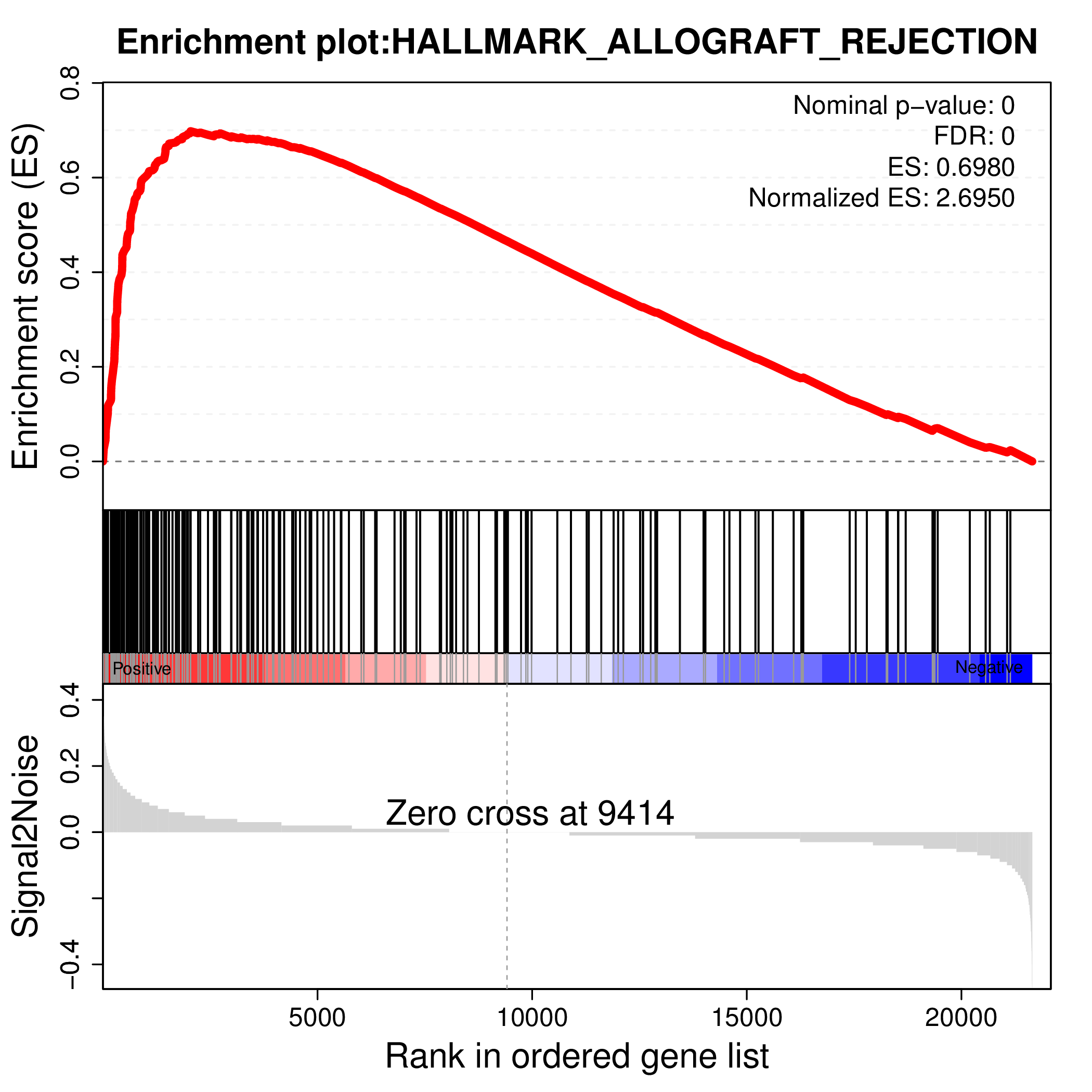

Supplement: Supplementary file 5 [file DataSheet4.ZIP › datasheet of Figure 4/GSE17536gsea/HALLMARK_ALLOGRAFT_REJECTION.enplot.png]

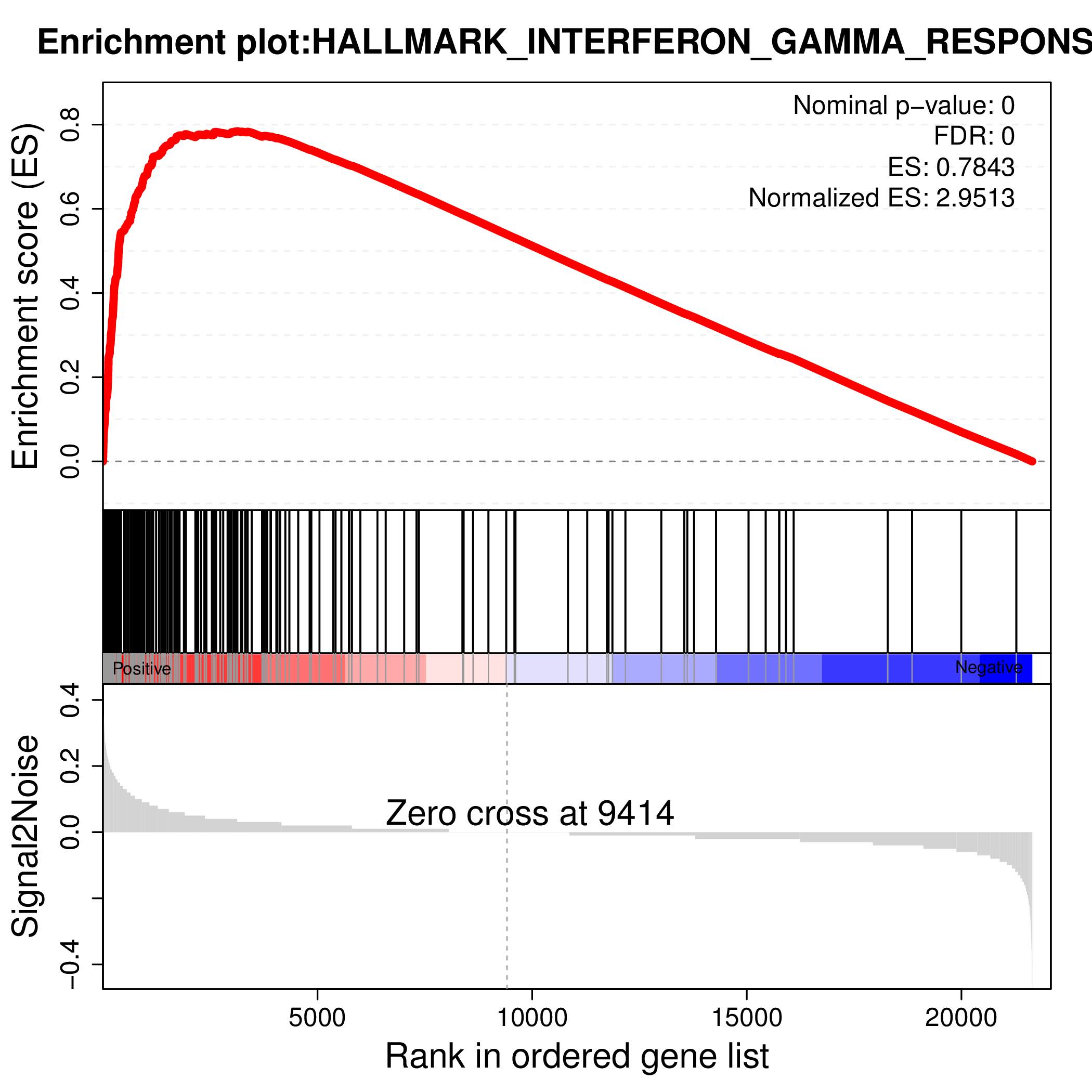

Supplement: Supplementary file 5 [file DataSheet4.ZIP › datasheet of Figure 4/GSE17536gsea/HALLMARK_INTERFERON_GAMMA_RESPONSE.enplot.png]

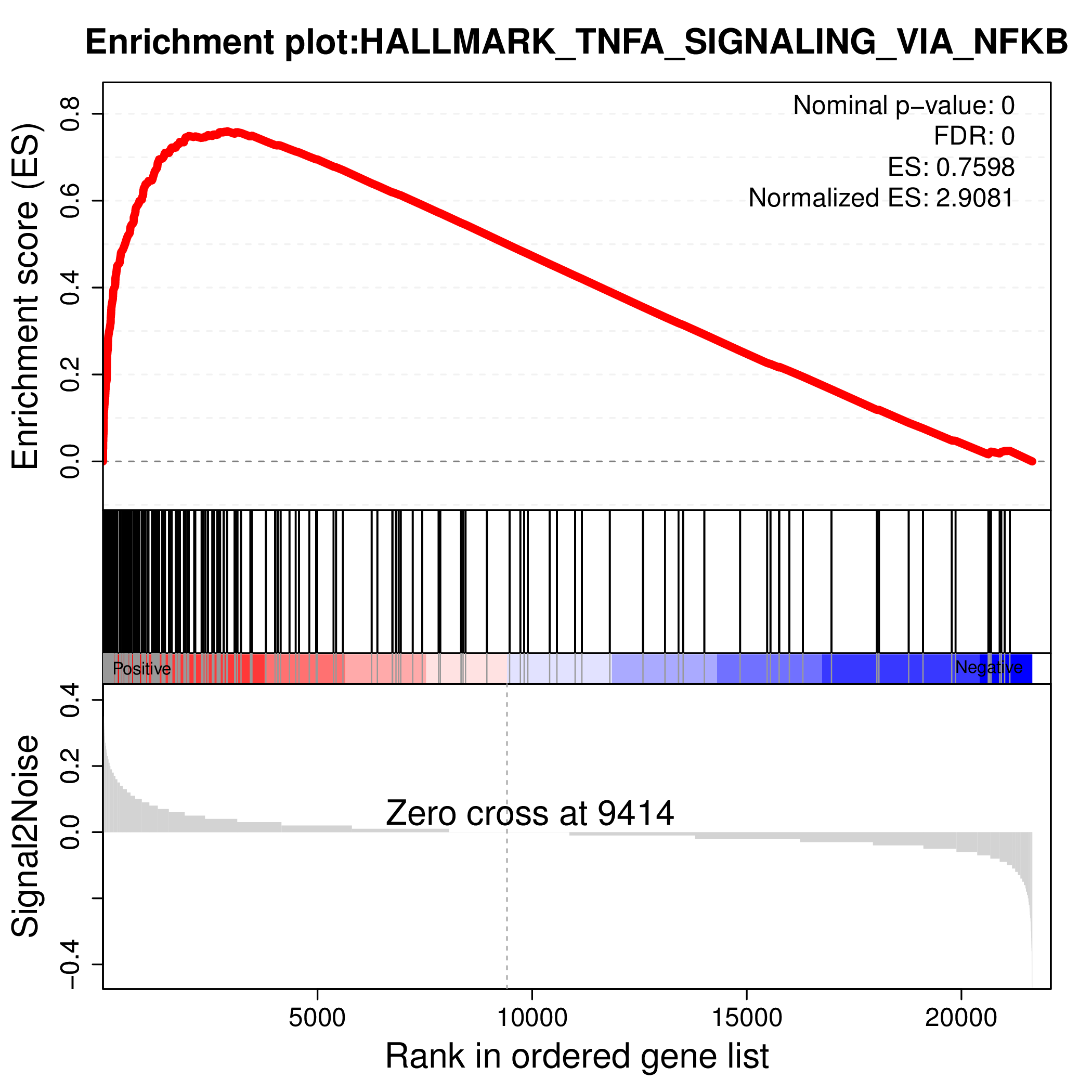

Supplement: Supplementary file 5 [file DataSheet4.ZIP › datasheet of Figure 4/GSE17536gsea/HALLMARK_TNFA_SIGNALING_VIA_NFKB.enplot.png]

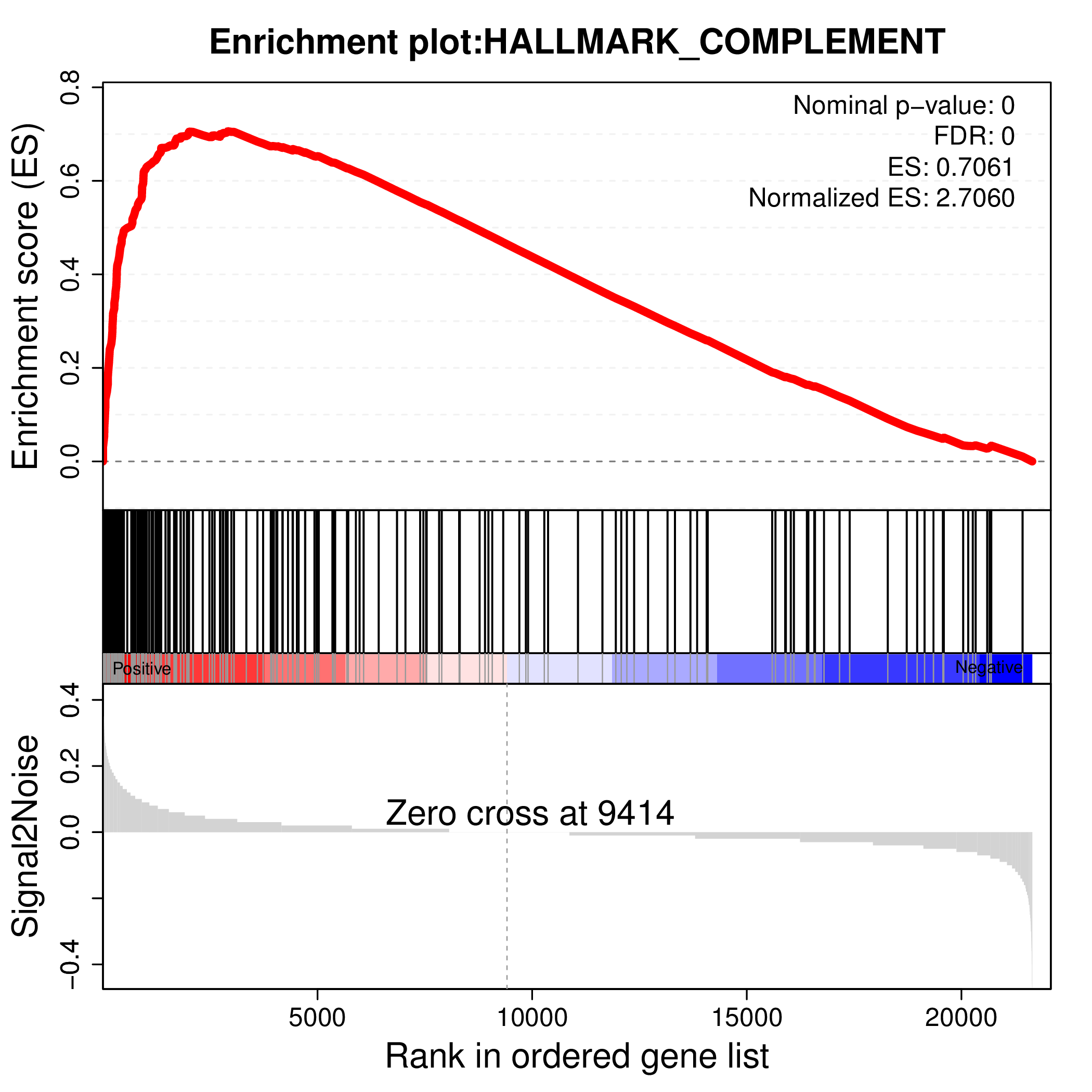

Supplement: Supplementary file 5 [file DataSheet4.ZIP › datasheet of Figure 4/GSE17536gsea/HALLMARK_COMPLEMENT.enplot.png]

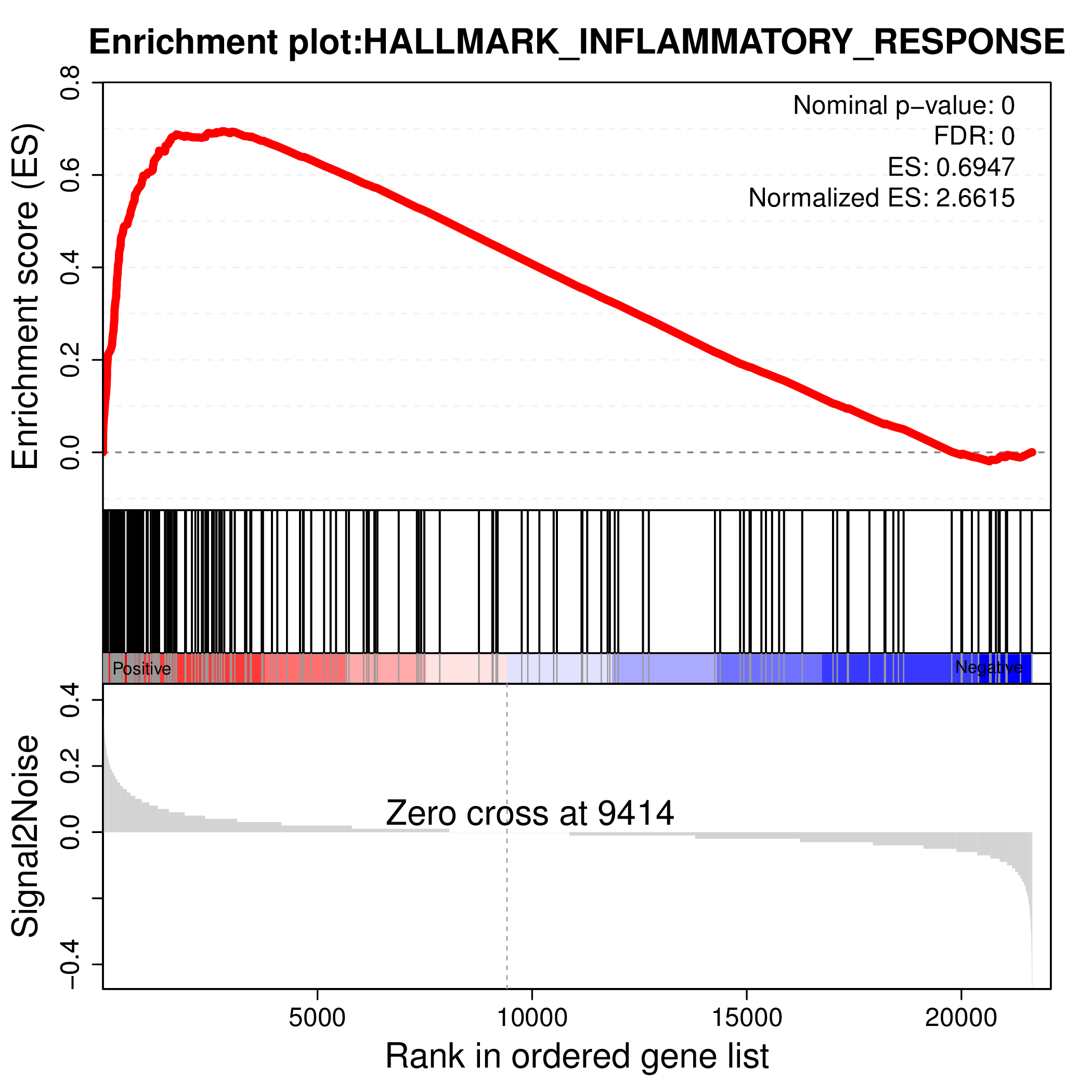

Supplement: Supplementary file 5 [file DataSheet4.ZIP › datasheet of Figure 4/GSE17536gsea/HALLMARK_INFLAMMATORY_RESPONSE.enplot.png]

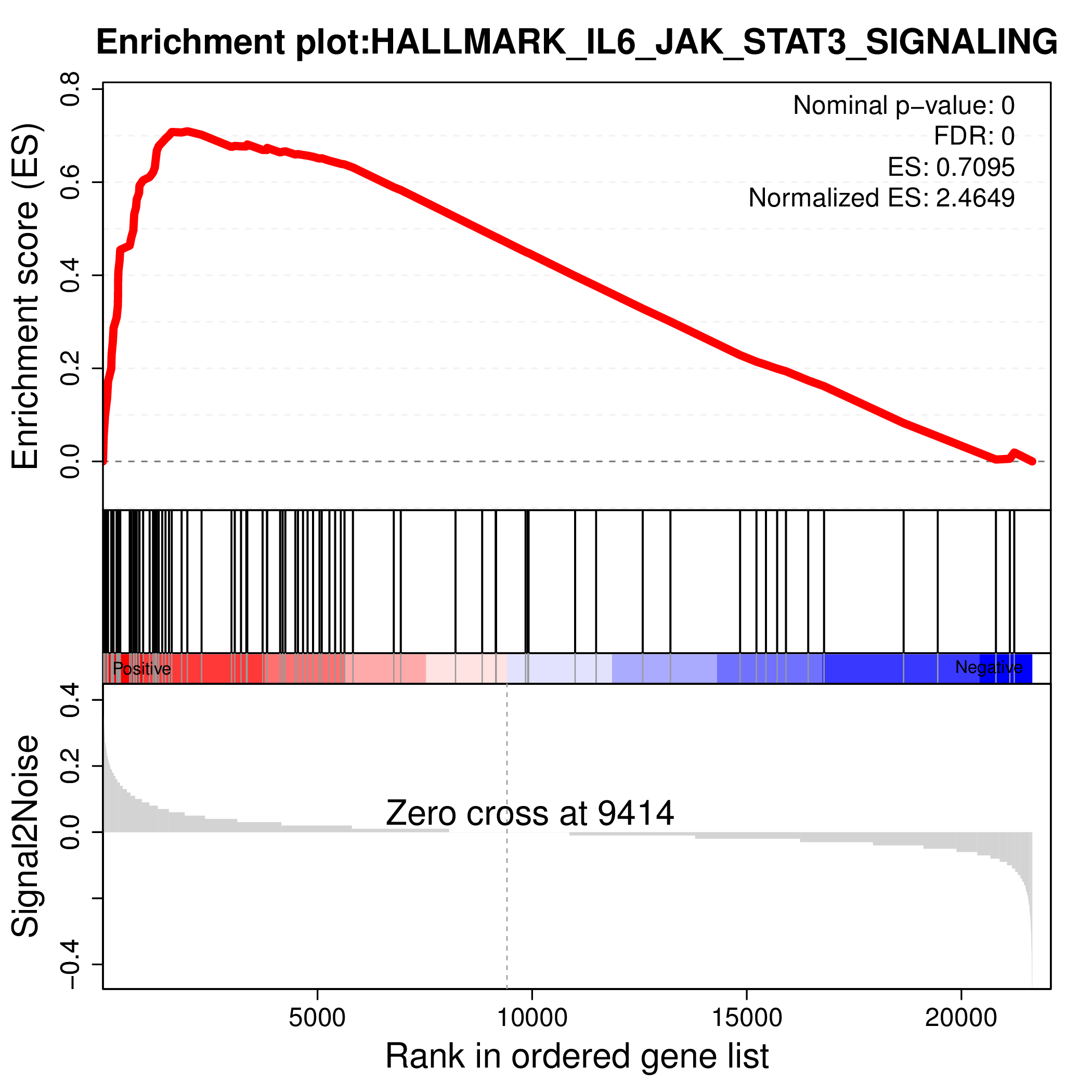

Supplement: Supplementary file 5 [file DataSheet4.ZIP › datasheet of Figure 4/GSE17536gsea/HALLMARK_IL6_JAK_STAT3_SIGNALING.enplot.png]

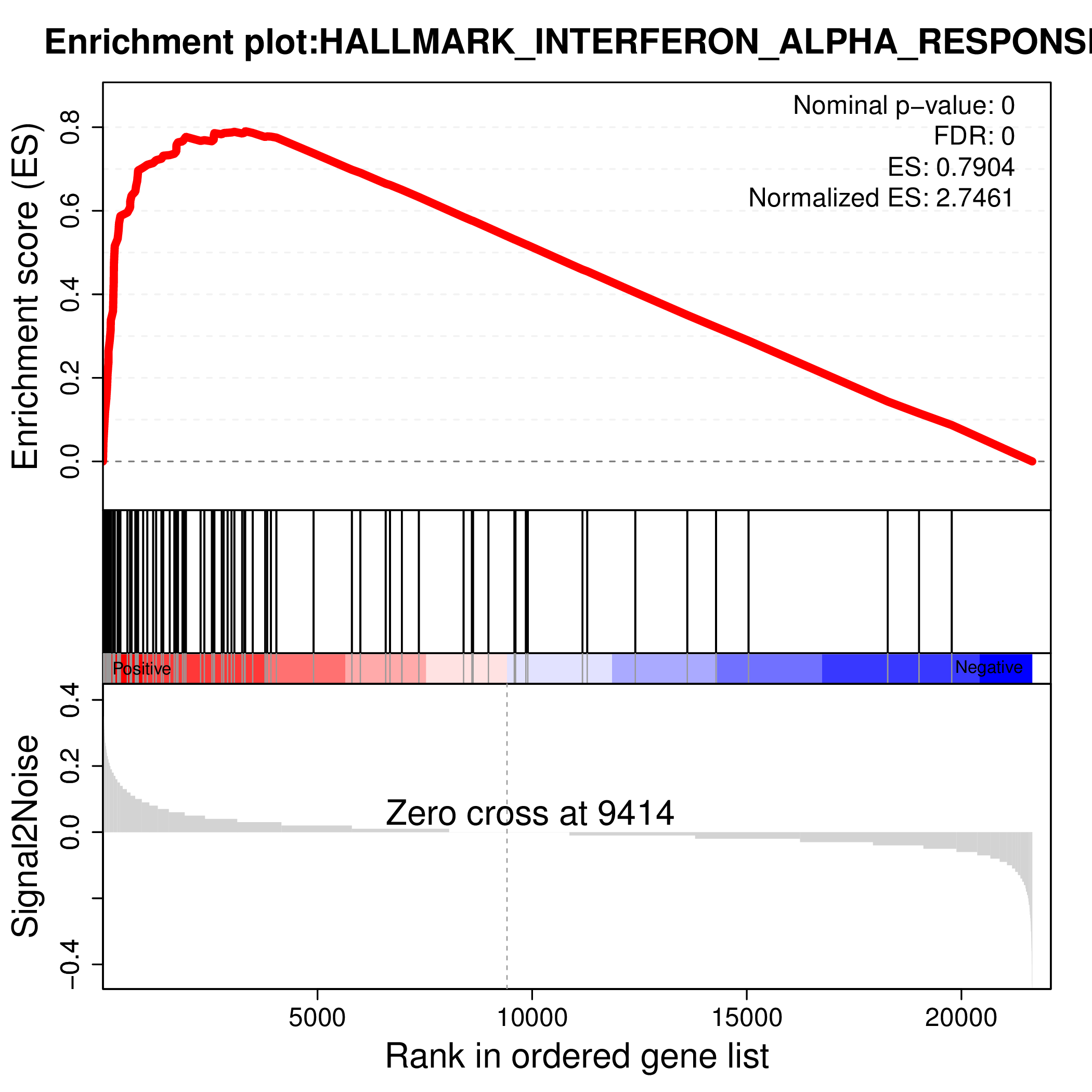

Supplement: Supplementary file 5 [file DataSheet4.ZIP › datasheet of Figure 4/GSE17536gsea/HALLMARK_INTERFERON_ALPHA_RESPONSE.enplot.png]

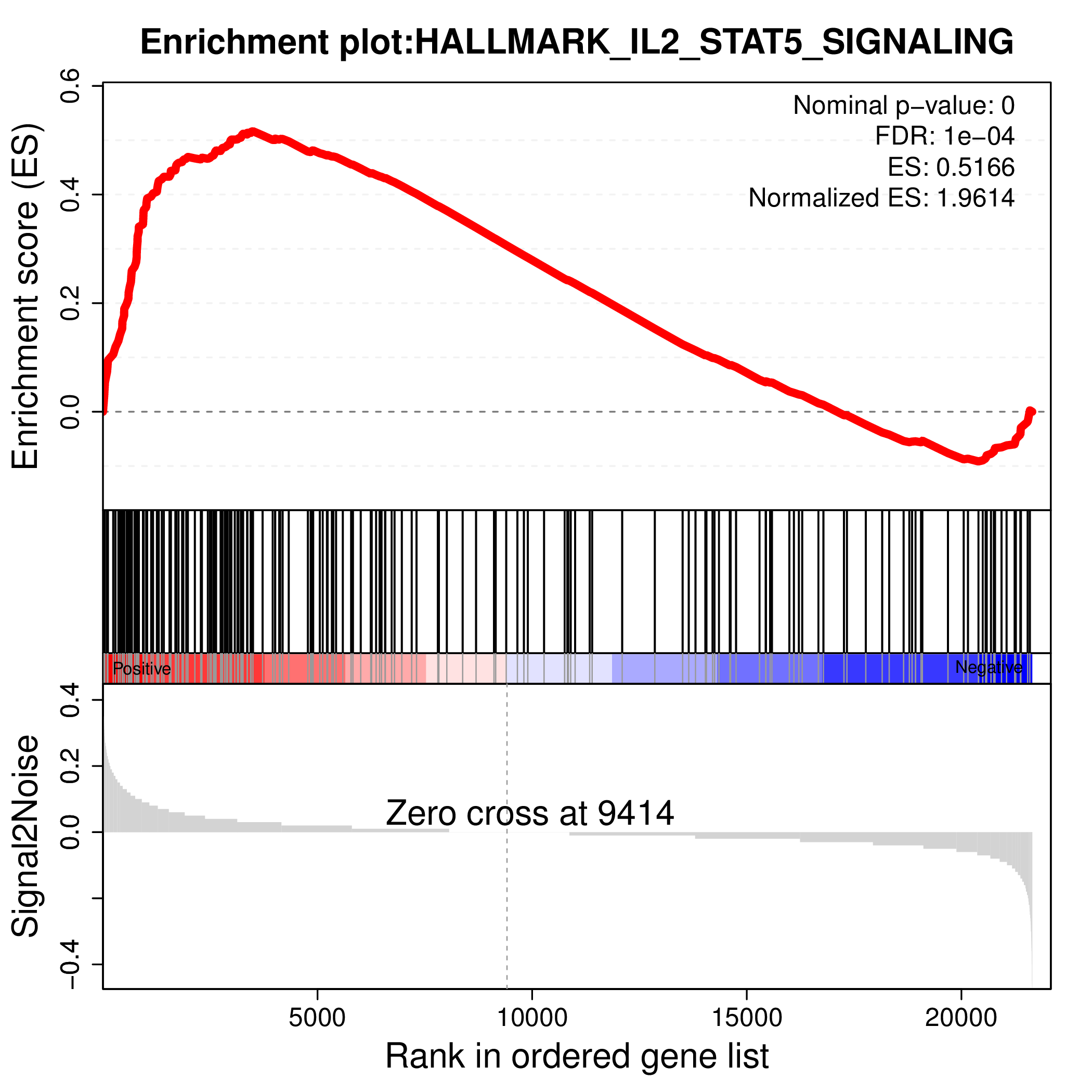

Supplement: Supplementary file 5 [file DataSheet4.ZIP › datasheet of Figure 4/GSE17536gsea/HALLMARK_IL2_STAT5_SIGNALING.enplot.png]

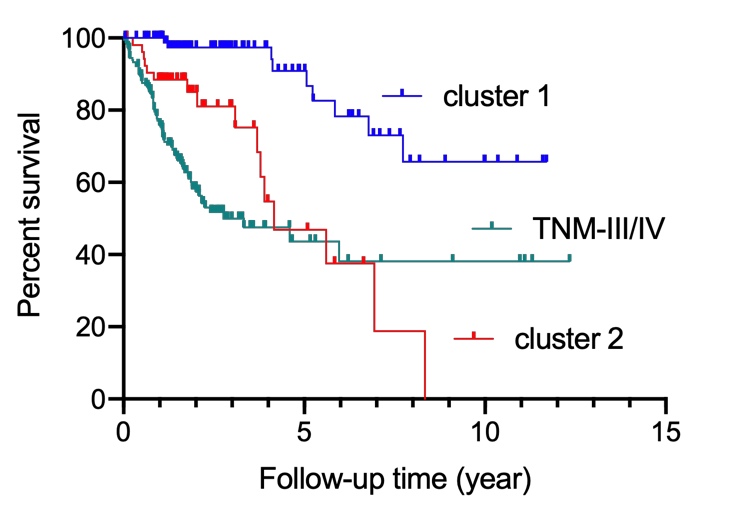

Supplement: Supplementary file 6 [file DataSheet1.ZIP › datasheet of Figure 1 /prognosisall.tiff]

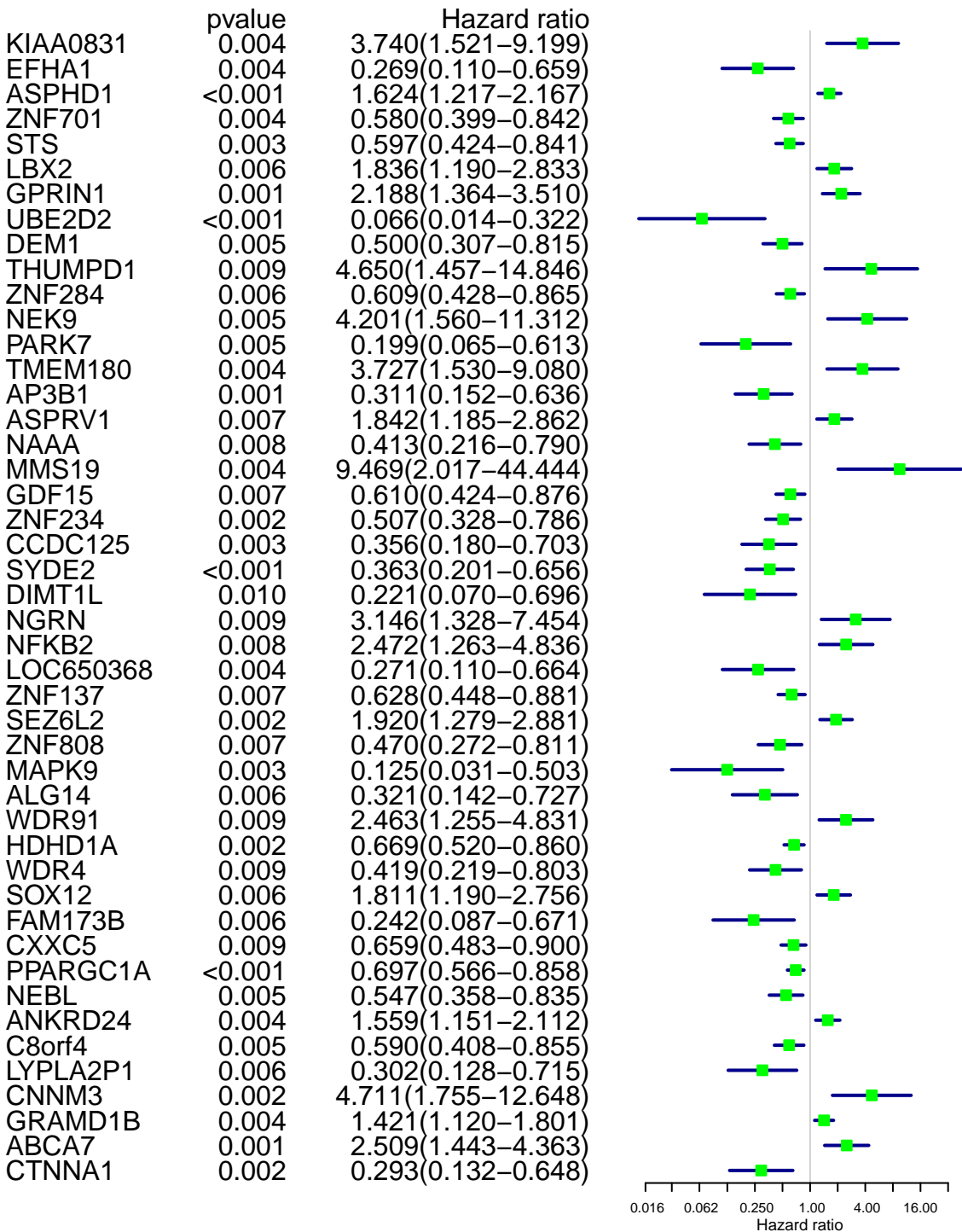

Supplement: Supplementary file 6 [file DataSheet1.ZIP › datasheet of Figure 1 /forest.pdf]

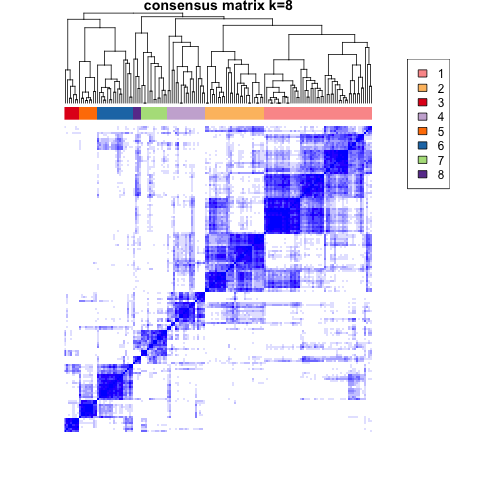

Supplement: Supplementary file 6 [file DataSheet1.ZIP › datasheet of Figure 1 /consensus008.png]

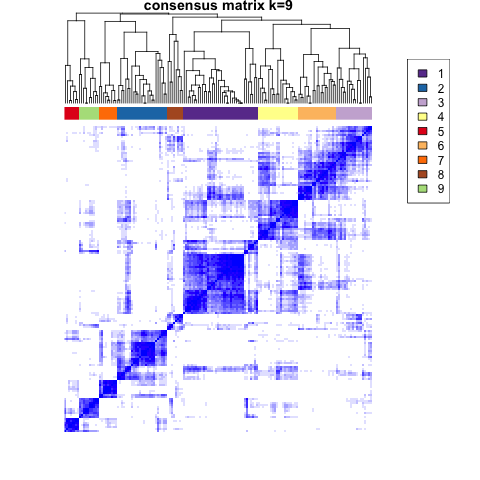

Supplement: Supplementary file 6 [file DataSheet1.ZIP › datasheet of Figure 1 /consensus009.png]

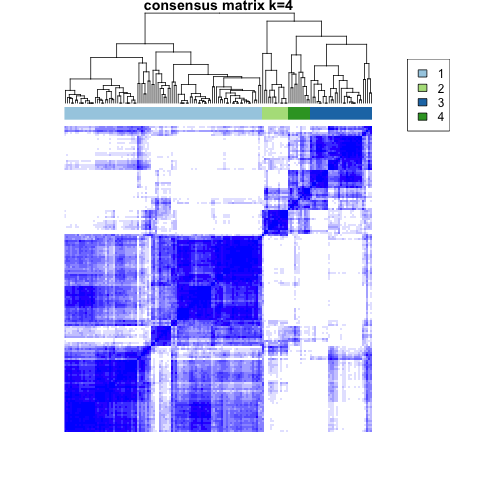

Supplement: Supplementary file 6 [file DataSheet1.ZIP › datasheet of Figure 1 /consensus004.png]

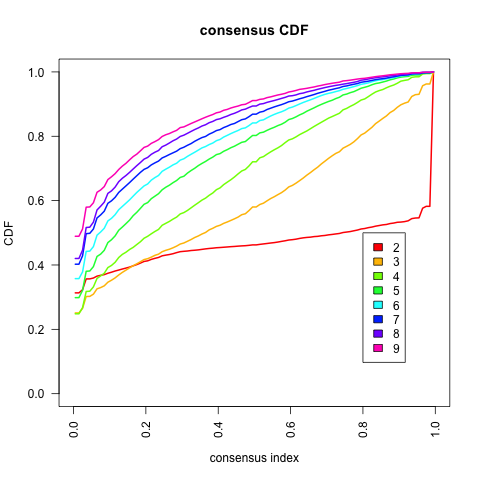

Supplement: Supplementary file 6 [file DataSheet1.ZIP › datasheet of Figure 1 /consensus010.png]

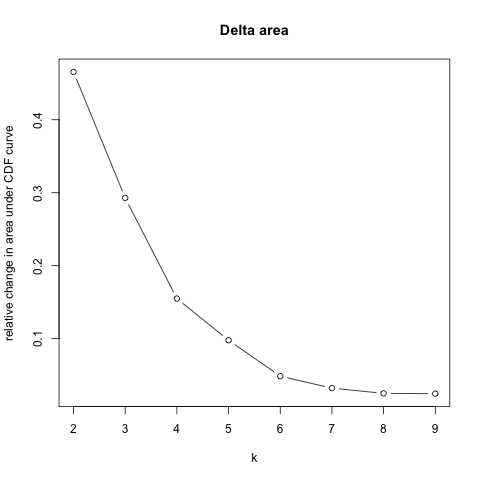

Supplement: Supplementary file 6 [file DataSheet1.ZIP › datasheet of Figure 1 /consensus011.png]

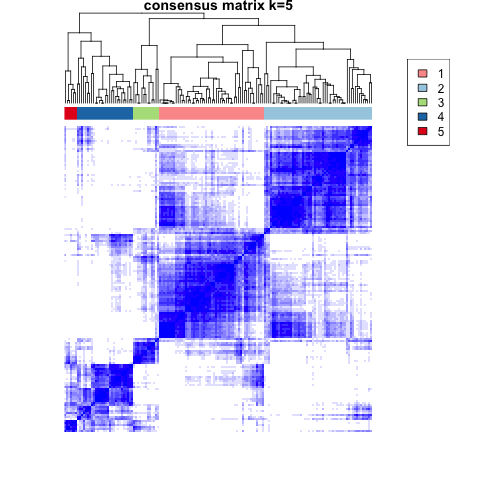

Supplement: Supplementary file 6 [file DataSheet1.ZIP › datasheet of Figure 1 /consensus005.png]

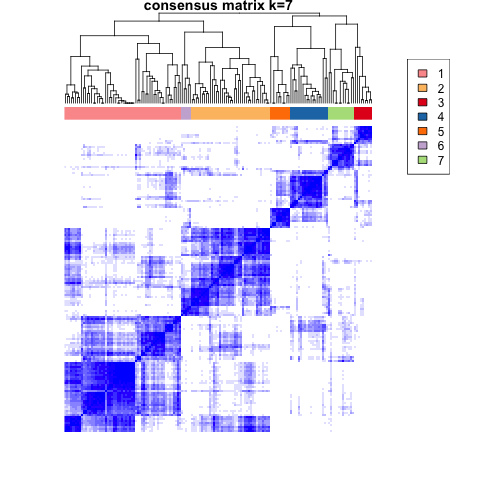

Supplement: Supplementary file 6 [file DataSheet1.ZIP › datasheet of Figure 1 /consensus007.png]

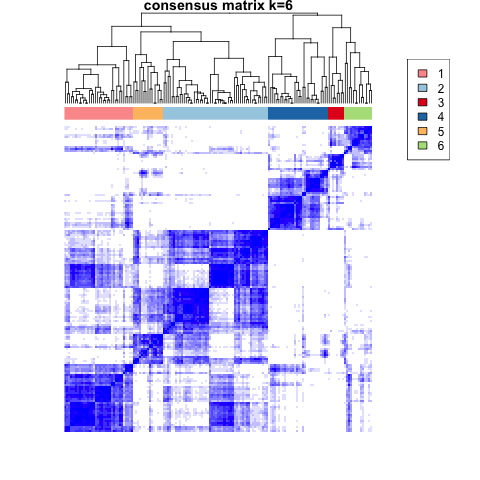

Supplement: Supplementary file 6 [file DataSheet1.ZIP › datasheet of Figure 1 /consensus006.png]

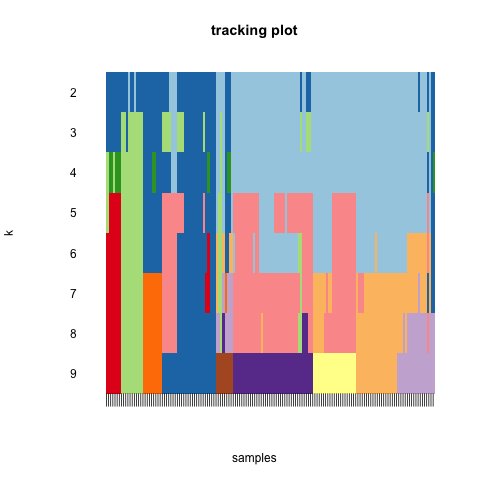

Supplement: Supplementary file 6 [file DataSheet1.ZIP › datasheet of Figure 1 /consensus012.png]

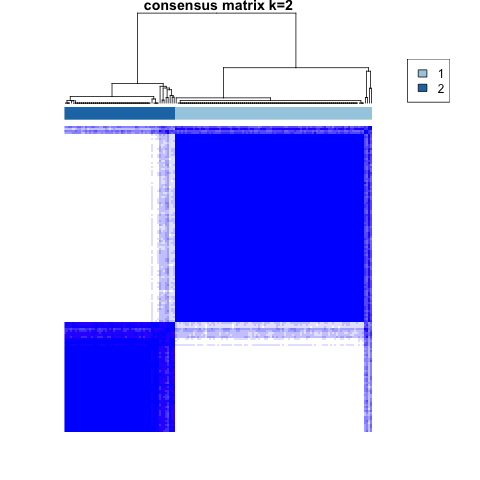

Supplement: Supplementary file 6 [file DataSheet1.ZIP › datasheet of Figure 1 /consensus002.png]

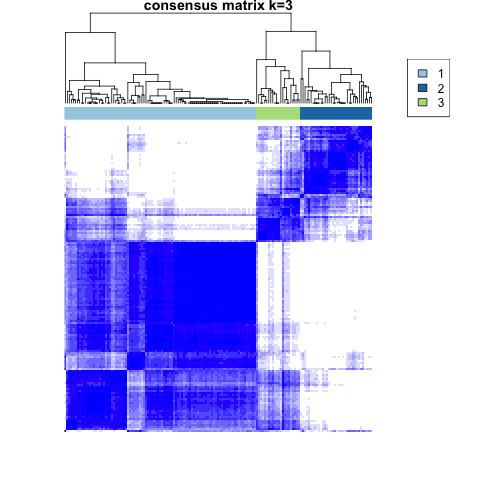

Supplement: Supplementary file 6 [file DataSheet1.ZIP › datasheet of Figure 1 /consensus003.png]

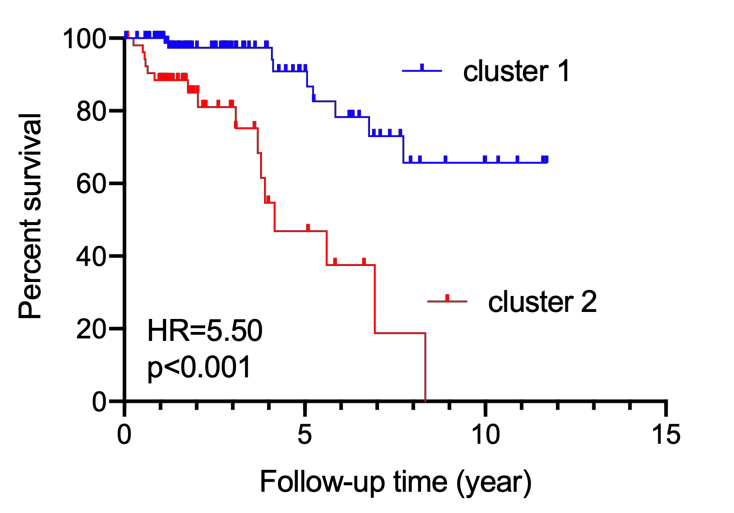

Supplement: Supplementary file 6 [file DataSheet1.ZIP › datasheet of Figure 1 /prognosis.tiff]

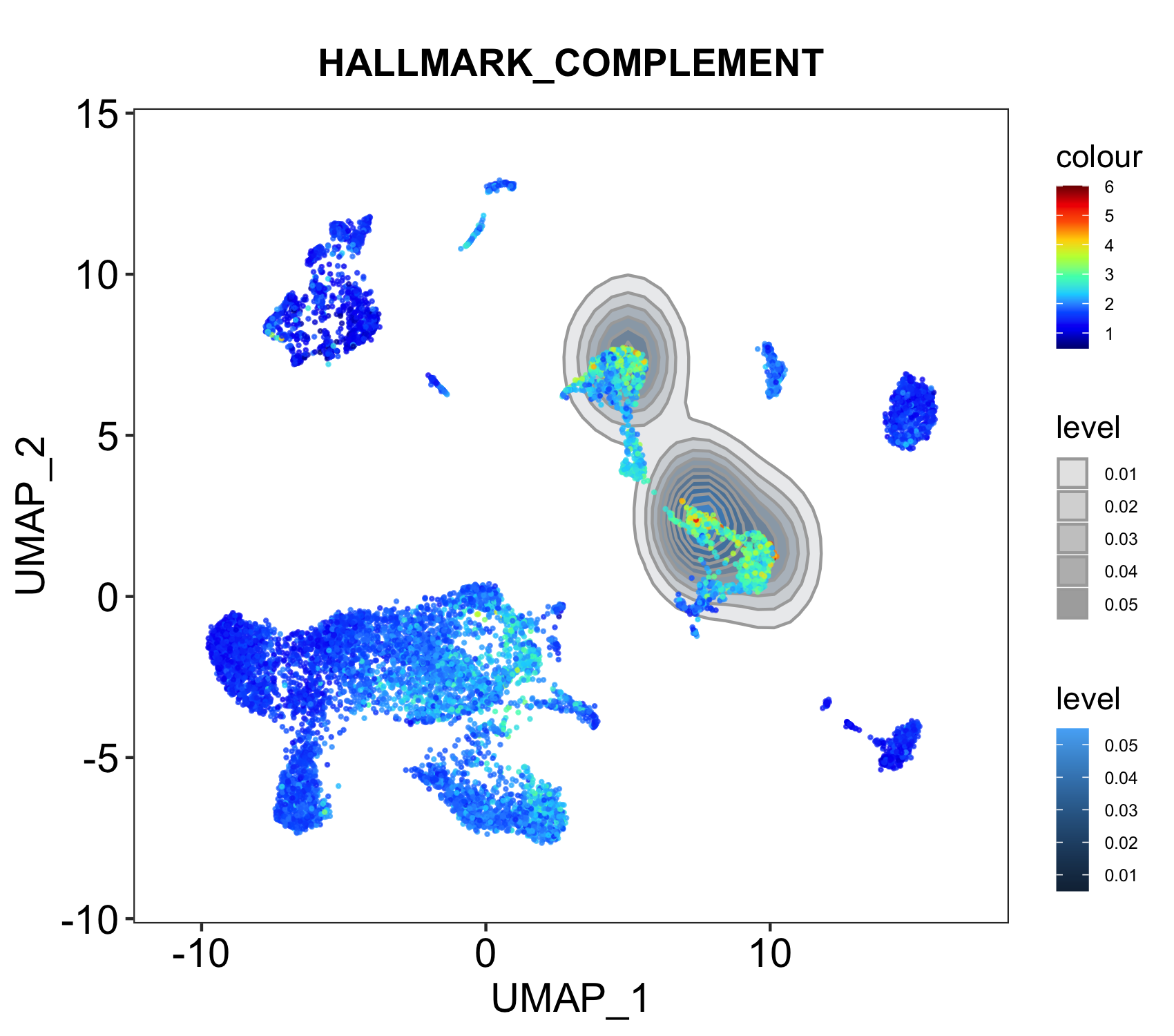

Supplement: Supplementary file 7 [file DataSheet6.ZIP › datasheet of Figure 6/CRC_GSE146771_Smartseq2_HALLMARK_COMPLEMENT_scSignatureExplorer_umap.png]

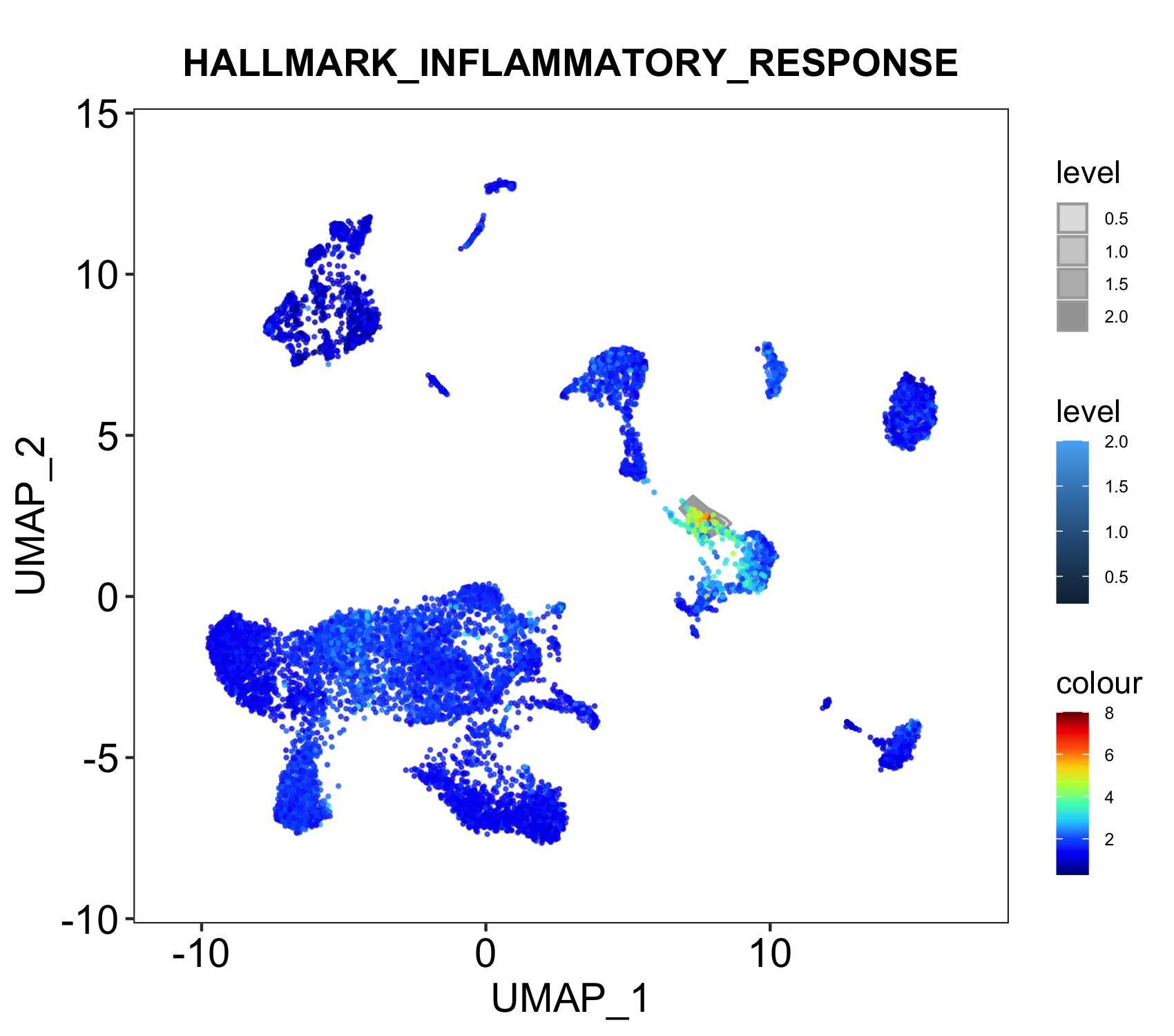

Supplement: Supplementary file 7 [file DataSheet6.ZIP › datasheet of Figure 6/CRC_GSE146771_Smartseq2_HALLMARK_INFLAMMATORY_RESPONSE_scSignatureExplorer_umap.png]

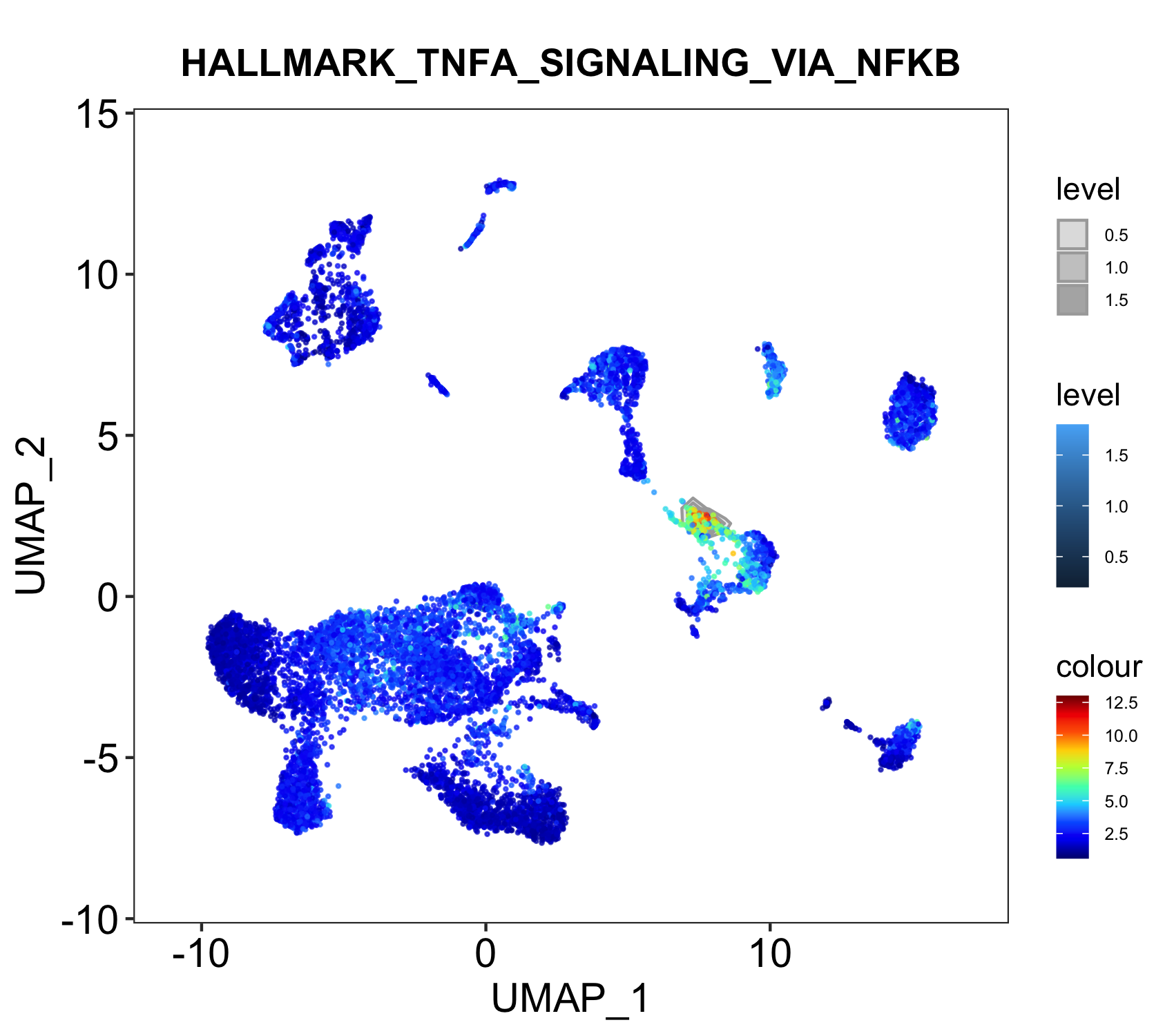

Supplement: Supplementary file 7 [file DataSheet6.ZIP › datasheet of Figure 6/CRC_GSE146771_Smartseq2_HALLMARK_TNFA_SIGNALING_VIA_NFKB_scSignatureExplorer_umap.png]

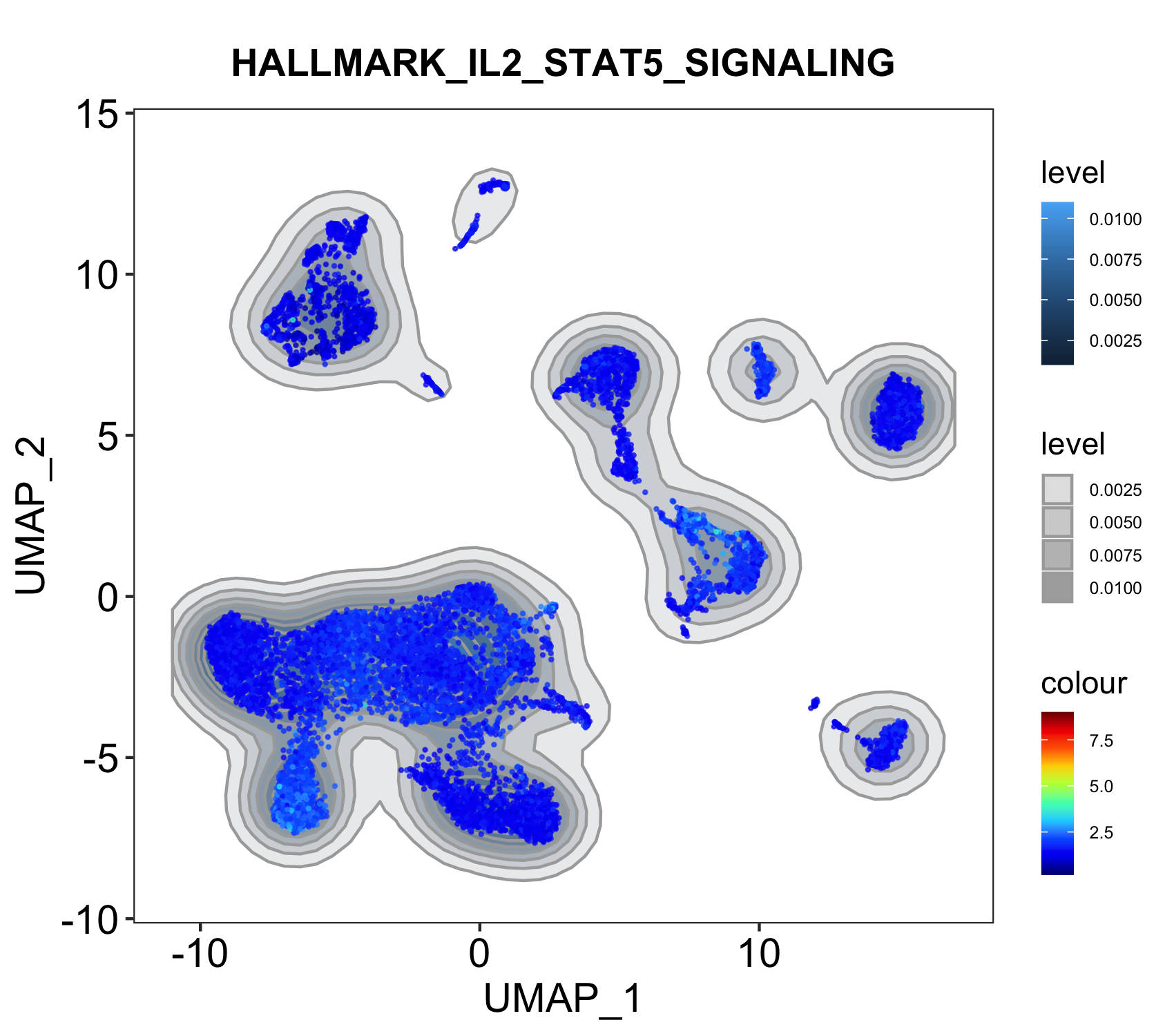

Supplement: Supplementary file 7 [file DataSheet6.ZIP › datasheet of Figure 6/CRC_GSE146771_Smartseq2_HALLMARK_IL2_STAT5_SIGNALING_scSignatureExplorer_umap.png]

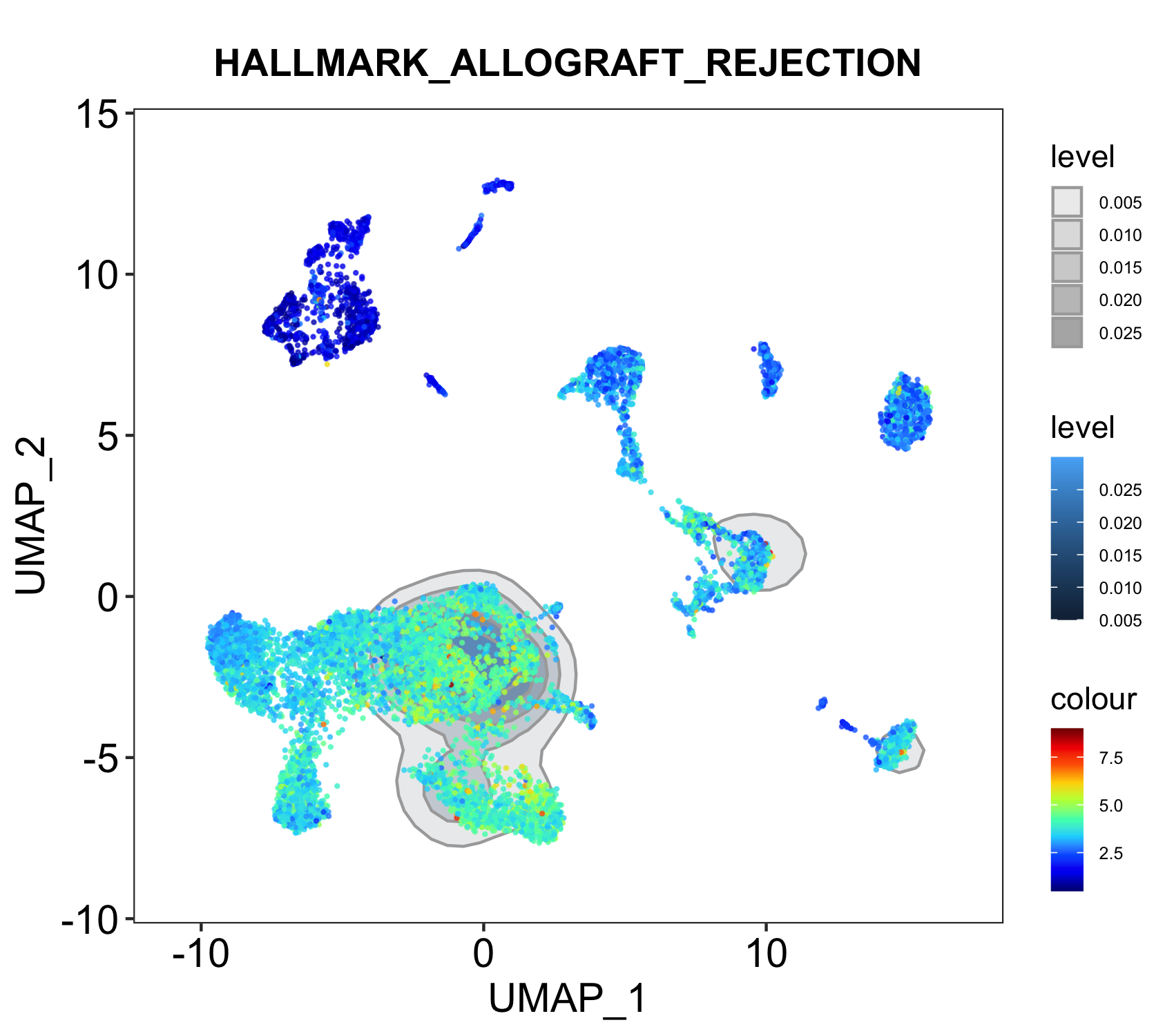

Supplement: Supplementary file 7 [file DataSheet6.ZIP › datasheet of Figure 6/CRC_GSE146771_Smartseq2_HALLMARK_ALLOGRAFT_REJECTION_scSignatureExplorer_umap.png]

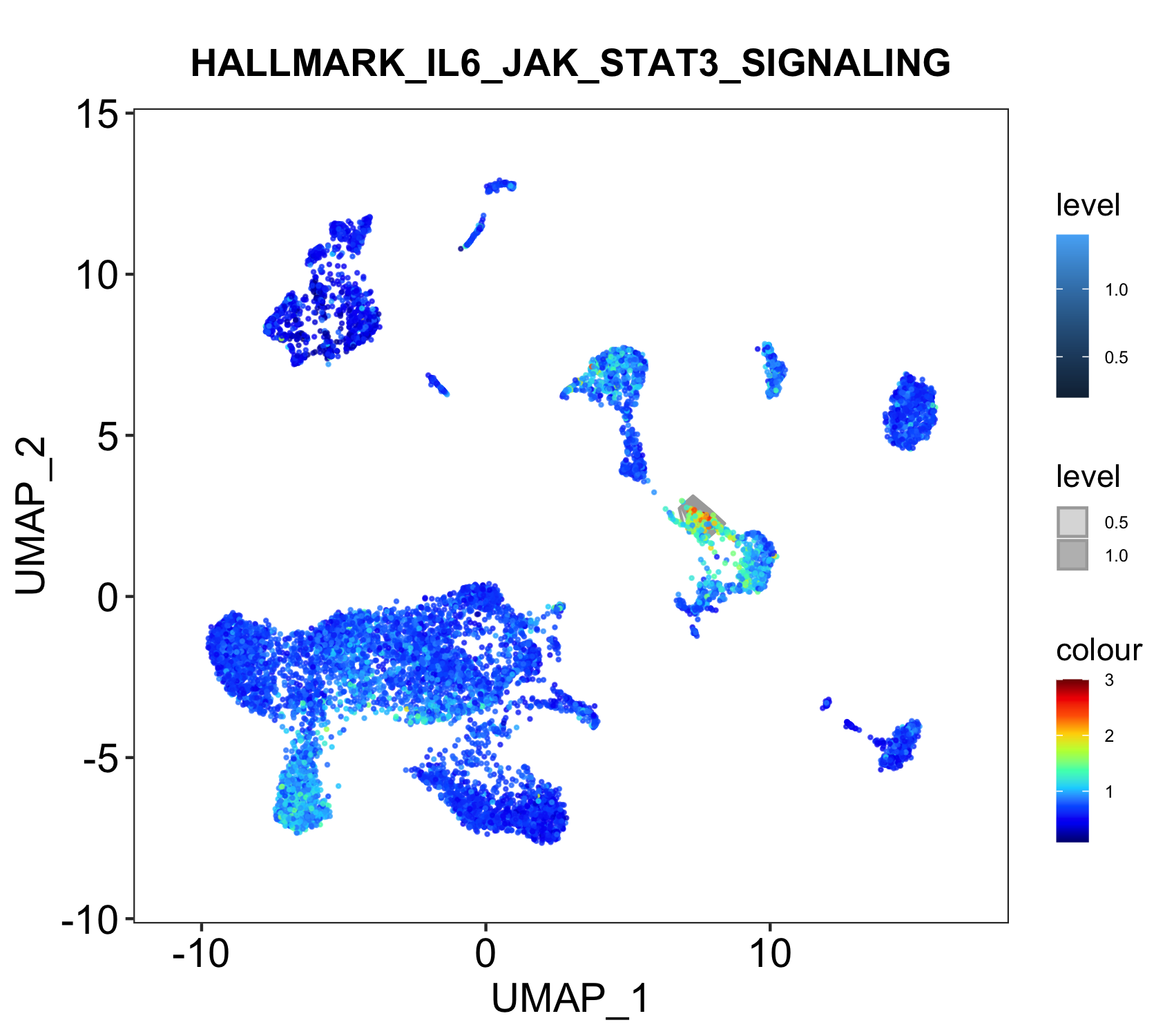

Supplement: Supplementary file 7 [file DataSheet6.ZIP › datasheet of Figure 6/CRC_GSE146771_Smartseq2_HALLMARK_IL6_JAK_STAT3_SIGNALING_scSignatureExplorer_umap.png]

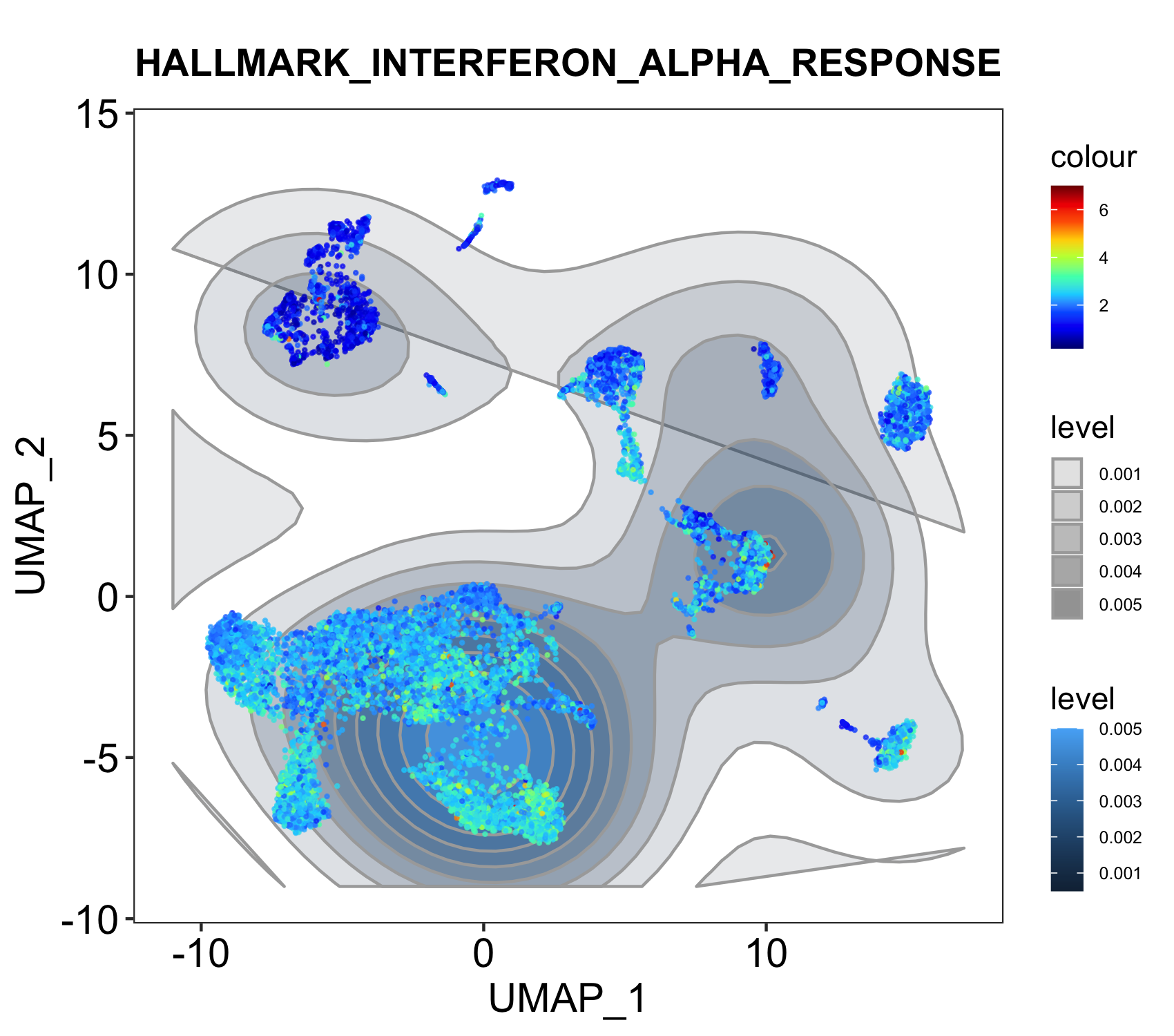

Supplement: Supplementary file 7 [file DataSheet6.ZIP › datasheet of Figure 6/CRC_GSE146771_Smartseq2_HALLMARK_INTERFERON_ALPHA_RESPONSE_scSignatureExplorer_umap.png]

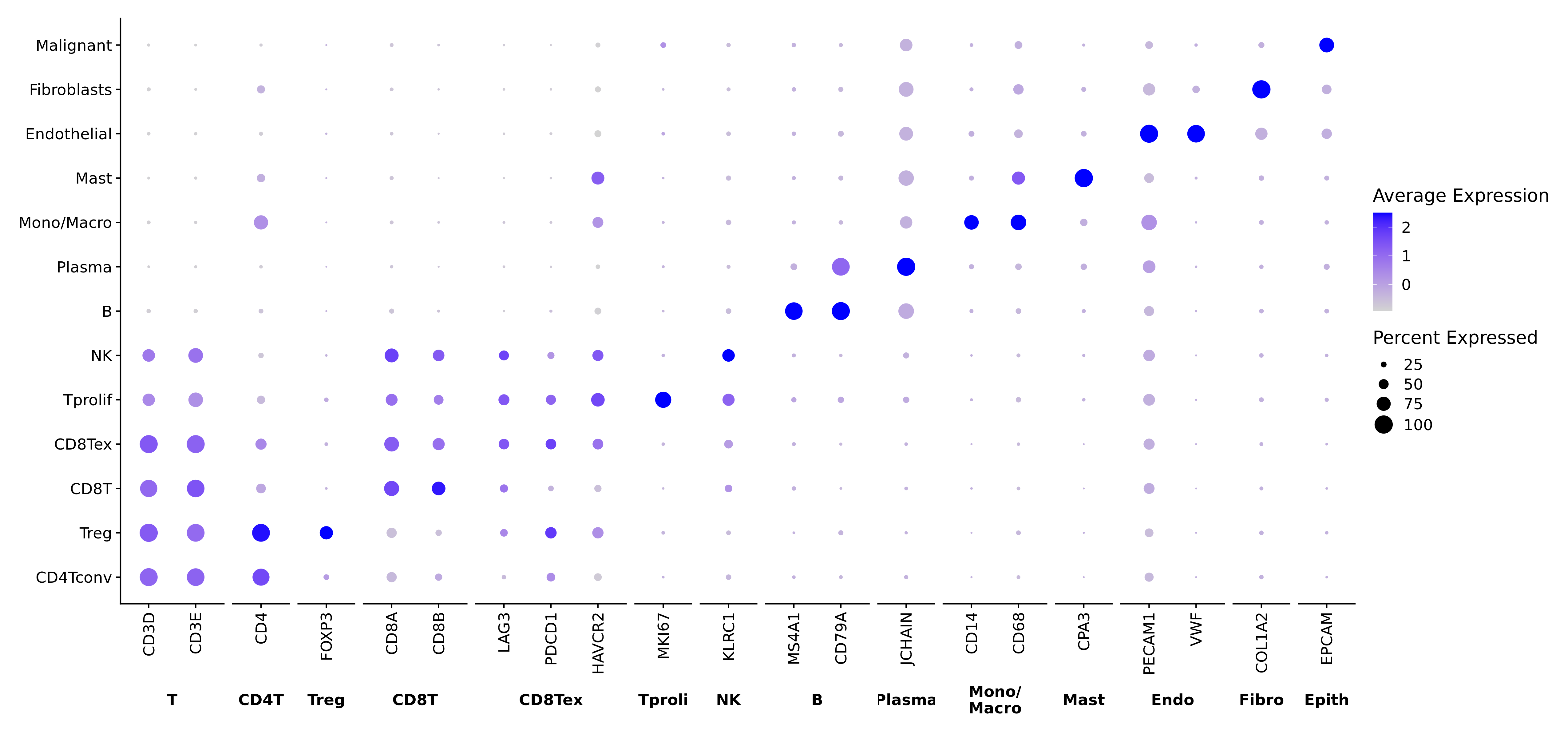

Supplement: Supplementary file 7 [file DataSheet6.ZIP › datasheet of Figure 6/CRC_GSE146771_Smartseq2_Dotplot.png]

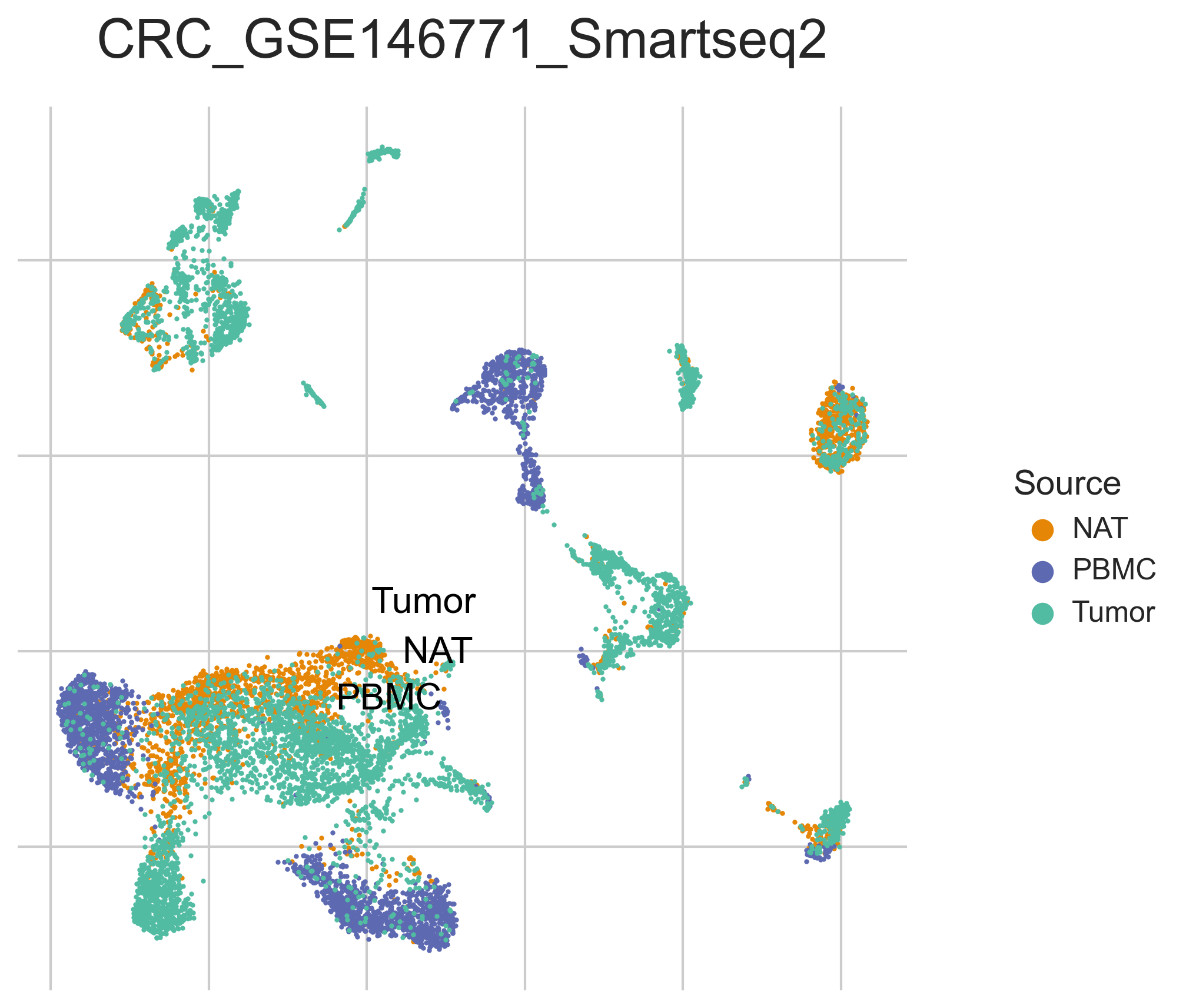

Supplement: Supplementary file 7 [file DataSheet6.ZIP › datasheet of Figure 6/CRC_GSE146771_Smartseq2_umap_Source.png]

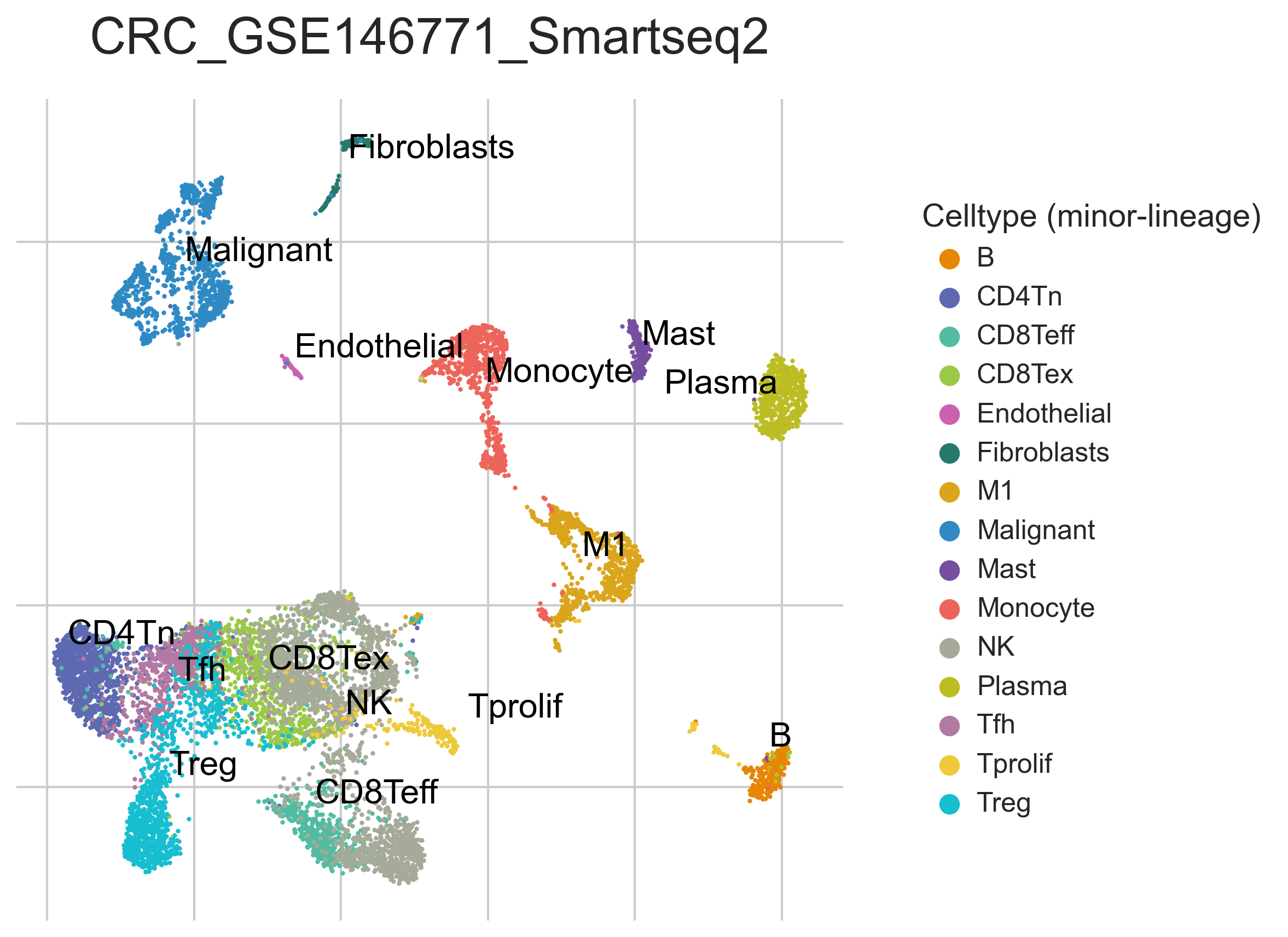

Supplement: Supplementary file 7 [file DataSheet6.ZIP › datasheet of Figure 6/CRC_GSE146771_Smartseq2_umap_Celltype_subtype.png]

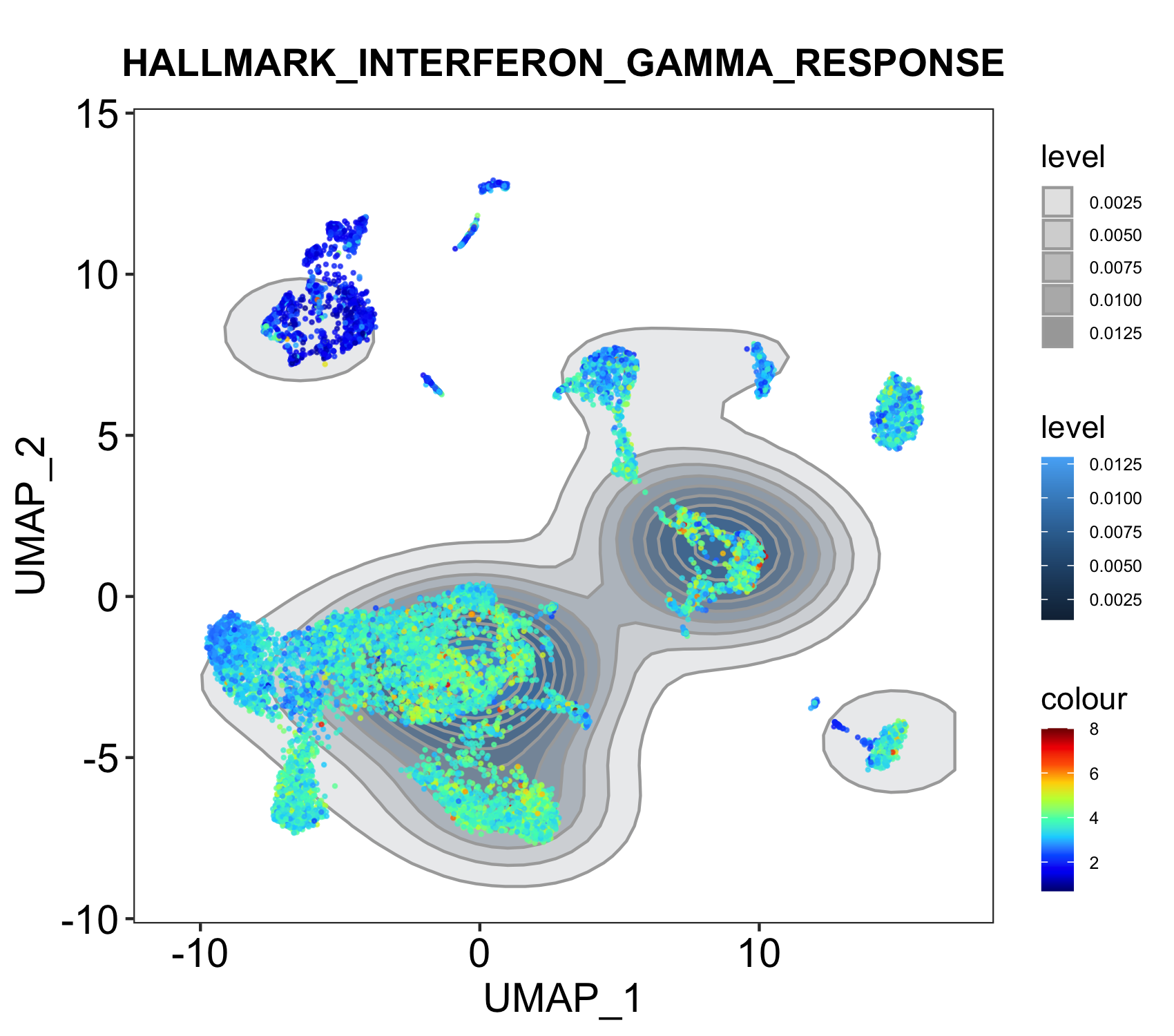

Supplement: Supplementary file 7 [file DataSheet6.ZIP › datasheet of Figure 6/CRC_GSE146771_Smartseq2_HALLMARK_INTERFERON_GAMMA_RESPONSE_scSignatureExplorer_umap.png]

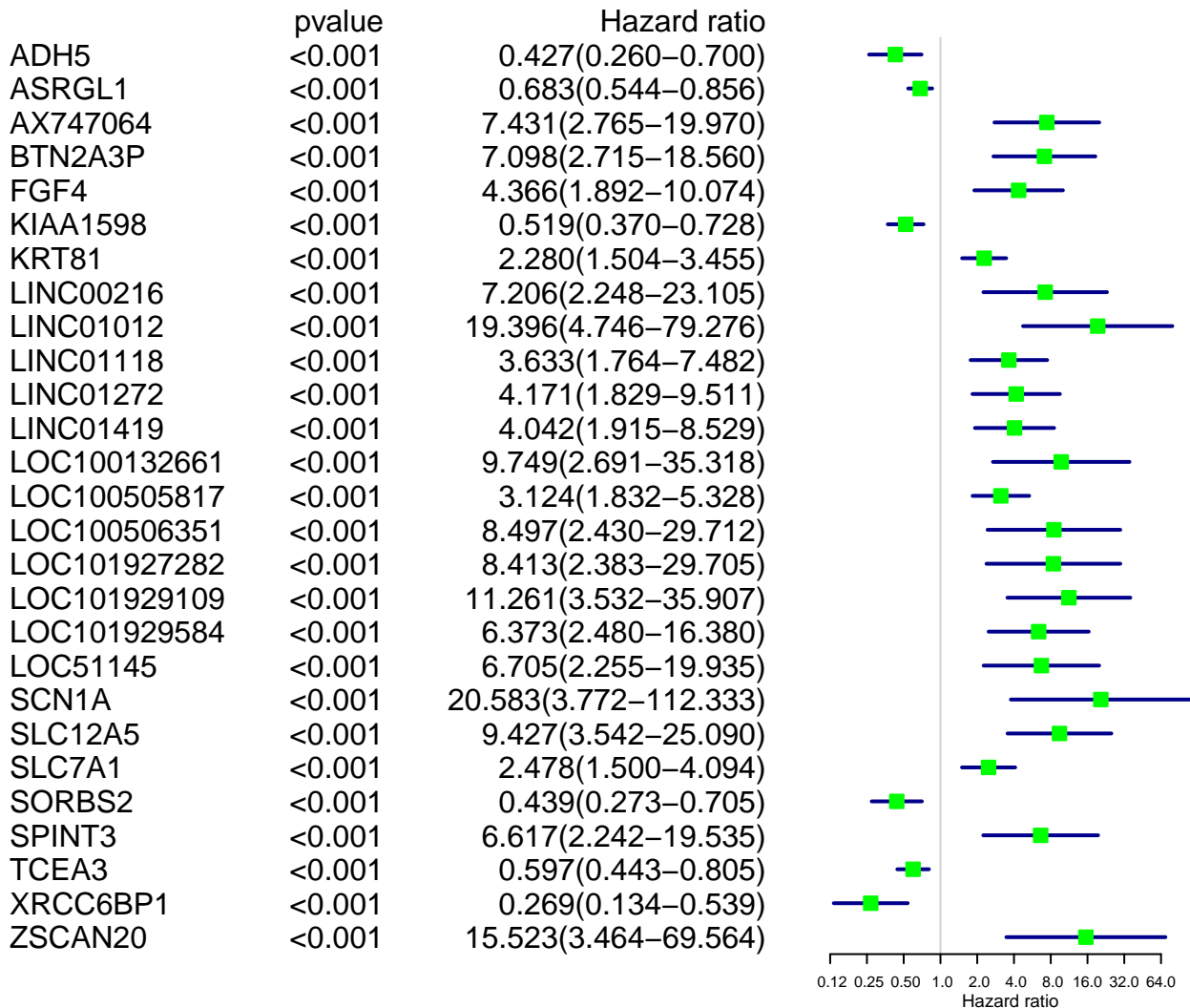

Supplement: Supplementary file 8 [file DataSheet2.ZIP › datasheet of Figure 2/forest.pdf]

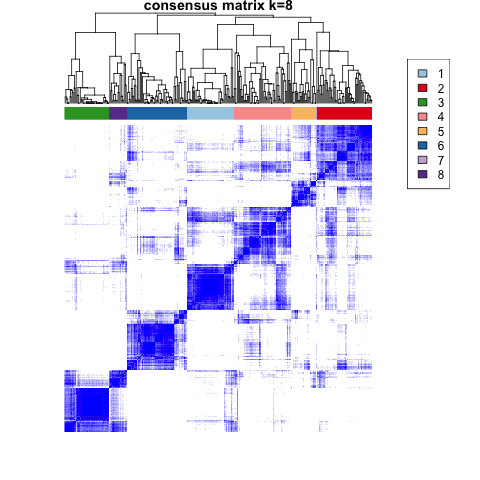

Supplement: Supplementary file 8 [file DataSheet2.ZIP › datasheet of Figure 2/consensus008.png]

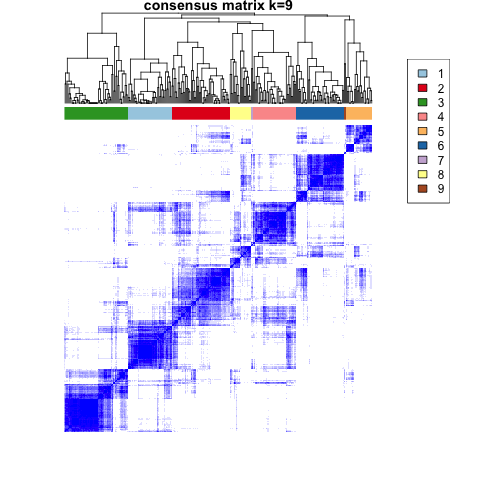

Supplement: Supplementary file 8 [file DataSheet2.ZIP › datasheet of Figure 2/consensus009.png]

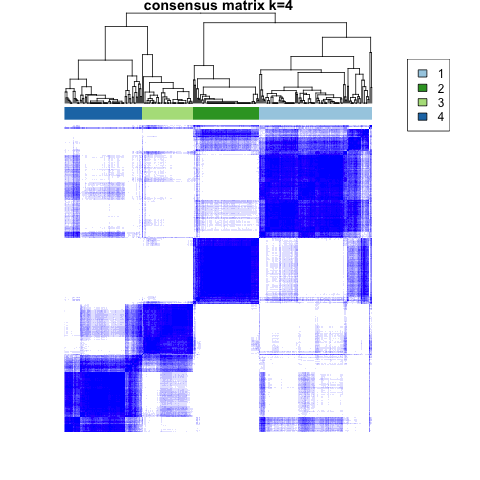

Supplement: Supplementary file 8 [file DataSheet2.ZIP › datasheet of Figure 2/consensus004.png]

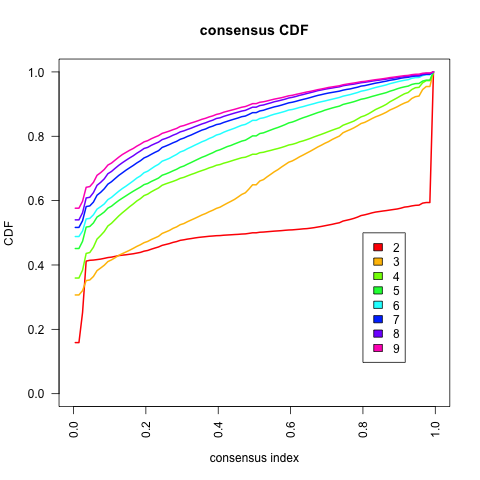

Supplement: Supplementary file 8 [file DataSheet2.ZIP › datasheet of Figure 2/consensus010.png]

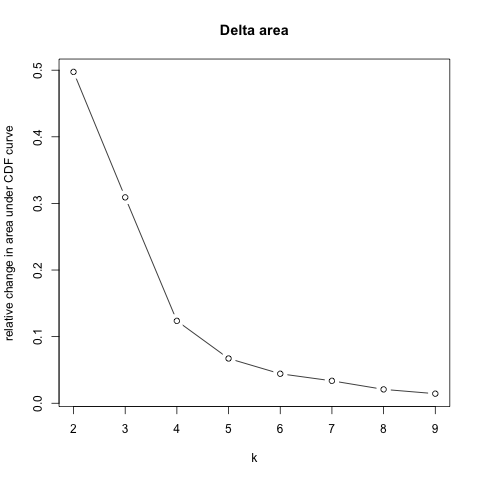

Supplement: Supplementary file 8 [file DataSheet2.ZIP › datasheet of Figure 2/consensus011.png]

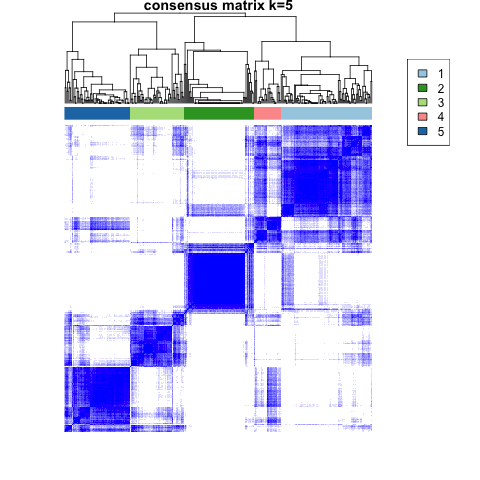

Supplement: Supplementary file 8 [file DataSheet2.ZIP › datasheet of Figure 2/consensus005.png]

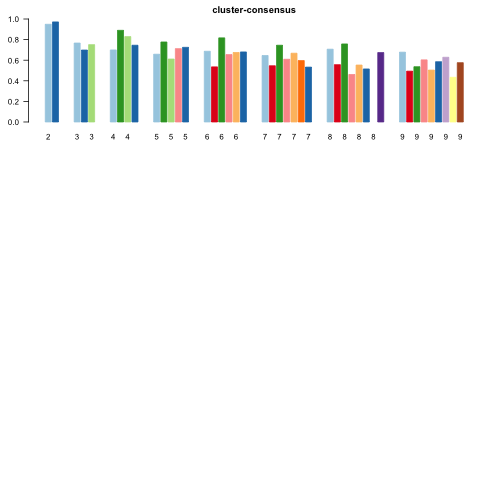

Supplement: Supplementary file 8 [file DataSheet2.ZIP › datasheet of Figure 2/consensusScore/icl004.png]

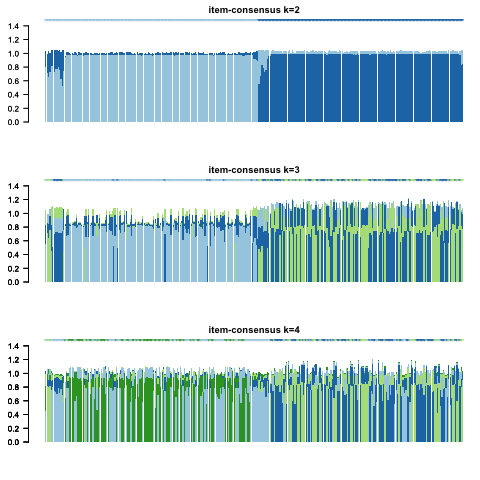

Supplement: Supplementary file 8 [file DataSheet2.ZIP › datasheet of Figure 2/consensusScore/icl001.png]

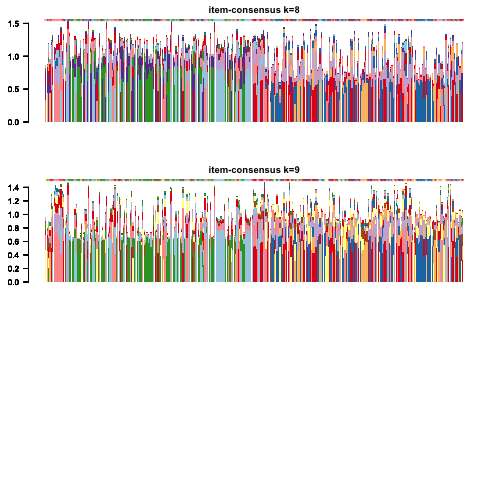

Supplement: Supplementary file 8 [file DataSheet2.ZIP › datasheet of Figure 2/consensusScore/icl003.png]

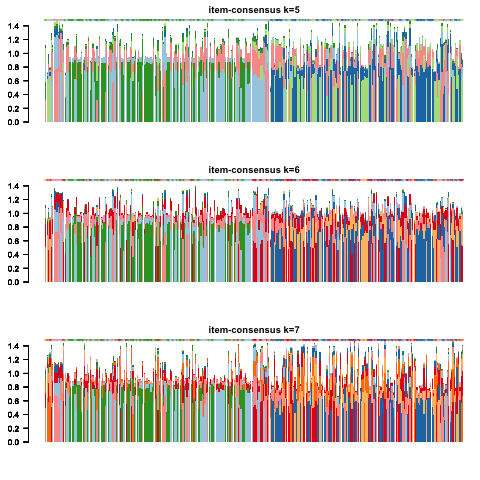

Supplement: Supplementary file 8 [file DataSheet2.ZIP › datasheet of Figure 2/consensusScore/icl002.png]

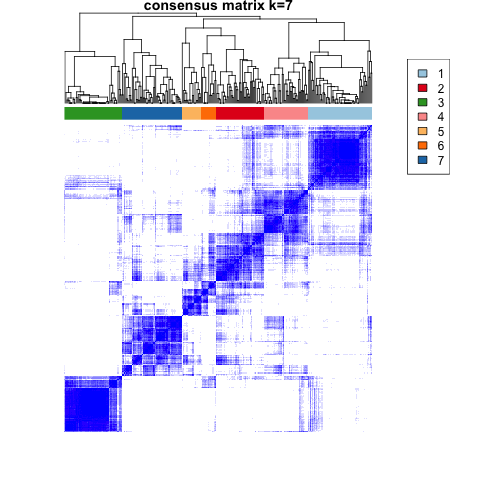

Supplement: Supplementary file 8 [file DataSheet2.ZIP › datasheet of Figure 2/consensus007.png]

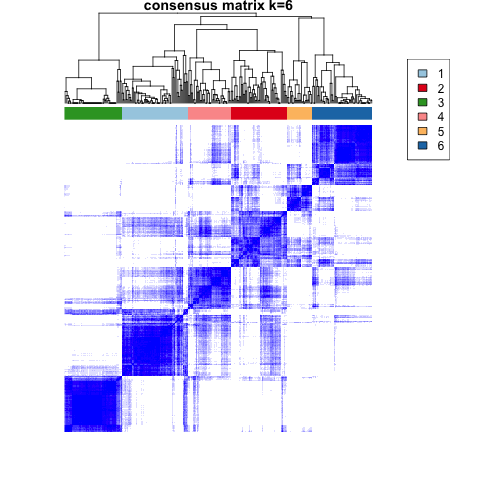

Supplement: Supplementary file 8 [file DataSheet2.ZIP › datasheet of Figure 2/consensus006.png]

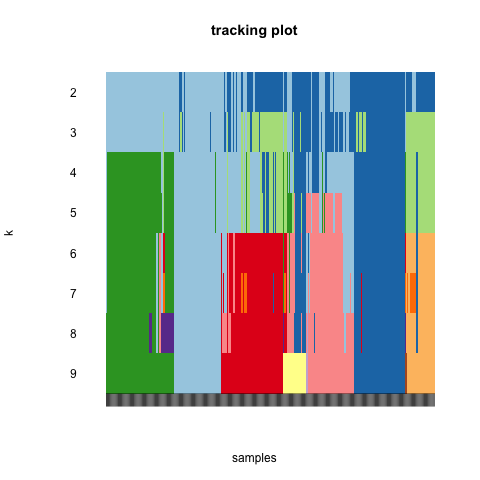

Supplement: Supplementary file 8 [file DataSheet2.ZIP › datasheet of Figure 2/consensus012.png]

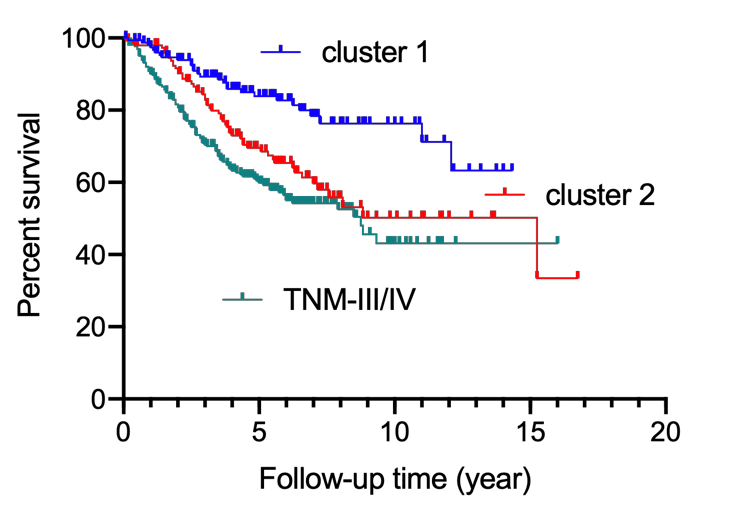

Supplement: Supplementary file 8 [file DataSheet2.ZIP › datasheet of Figure 2/39582OSall.tiff]

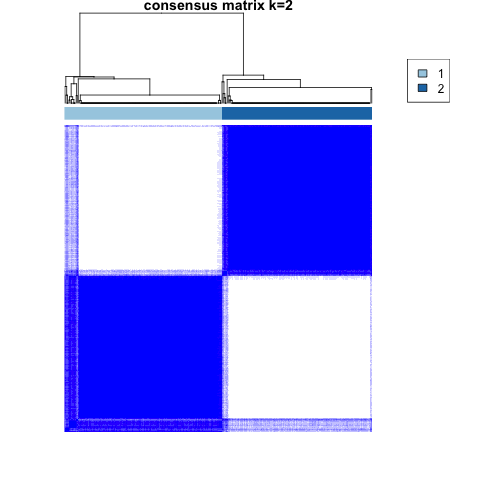

Supplement: Supplementary file 8 [file DataSheet2.ZIP › datasheet of Figure 2/consensus002.png]

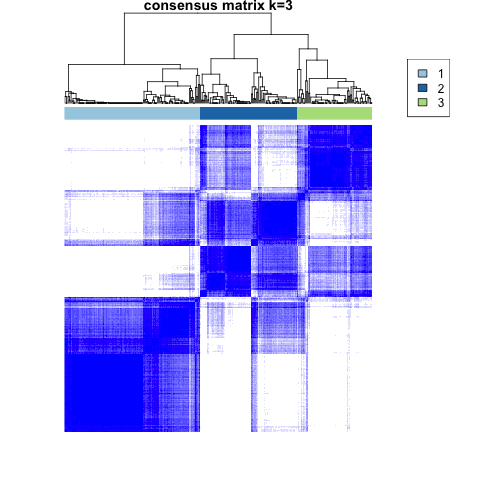

Supplement: Supplementary file 8 [file DataSheet2.ZIP › datasheet of Figure 2/consensus003.png]

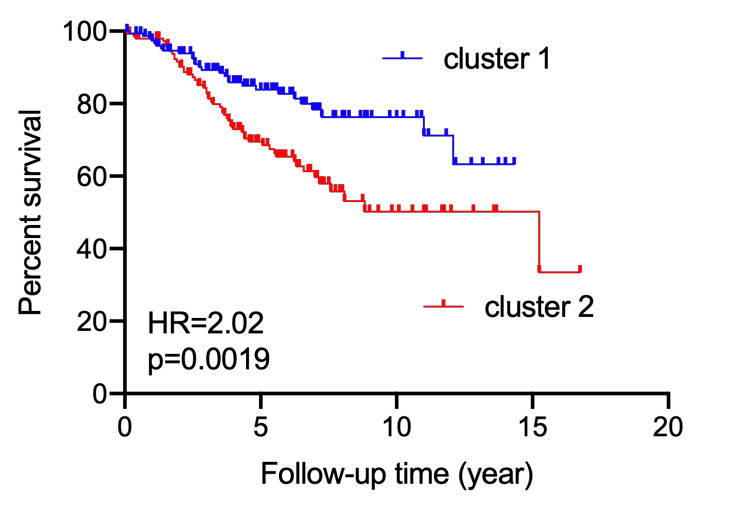

Supplement: Supplementary file 8 [file DataSheet2.ZIP › datasheet of Figure 2/prognosis.tiff]

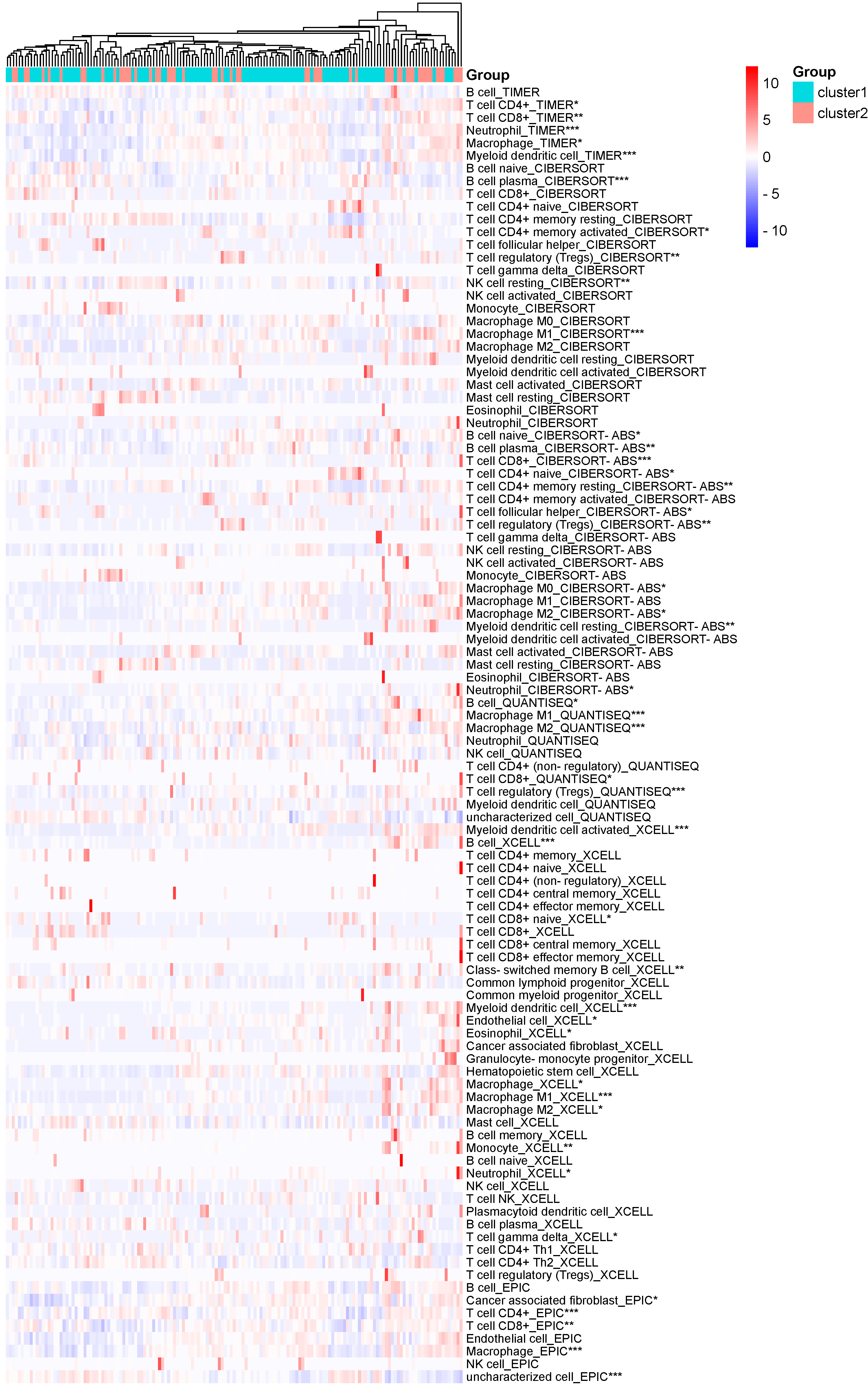

Supplement: Supplementary file 9 [file DataSheet5.ZIP › datasheet of Figure 5/TCGA/1653902977.37.png]

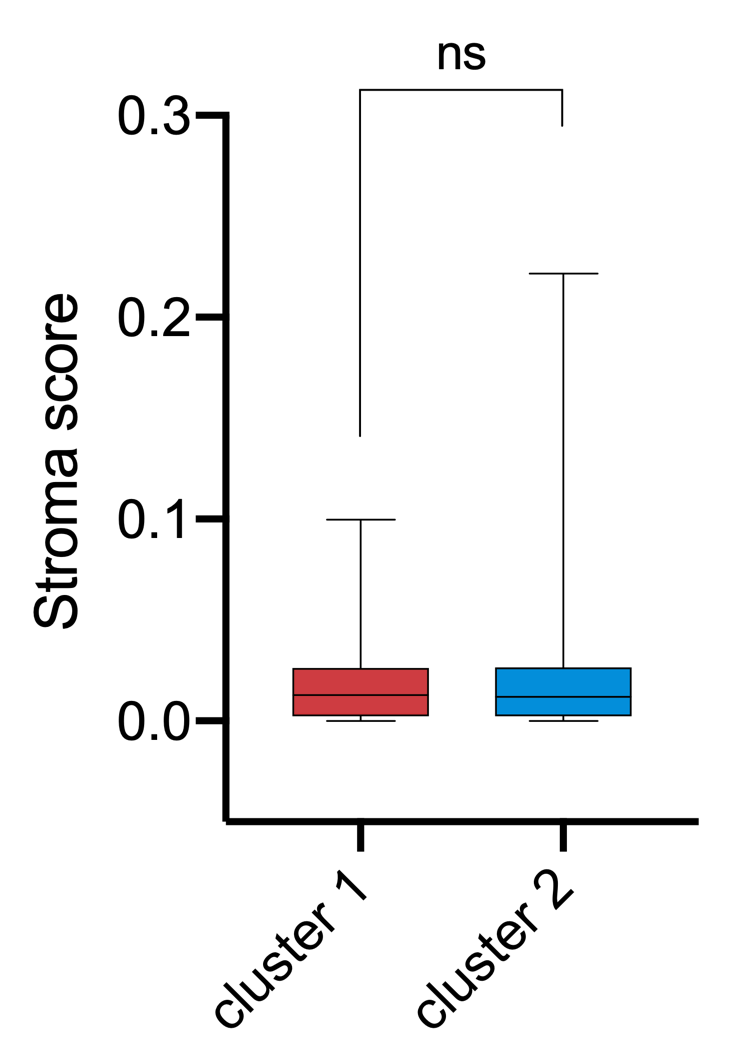

Supplement: Supplementary file 9 [file DataSheet5.ZIP › datasheet of Figure 5/TCGA/stroma.tiff]

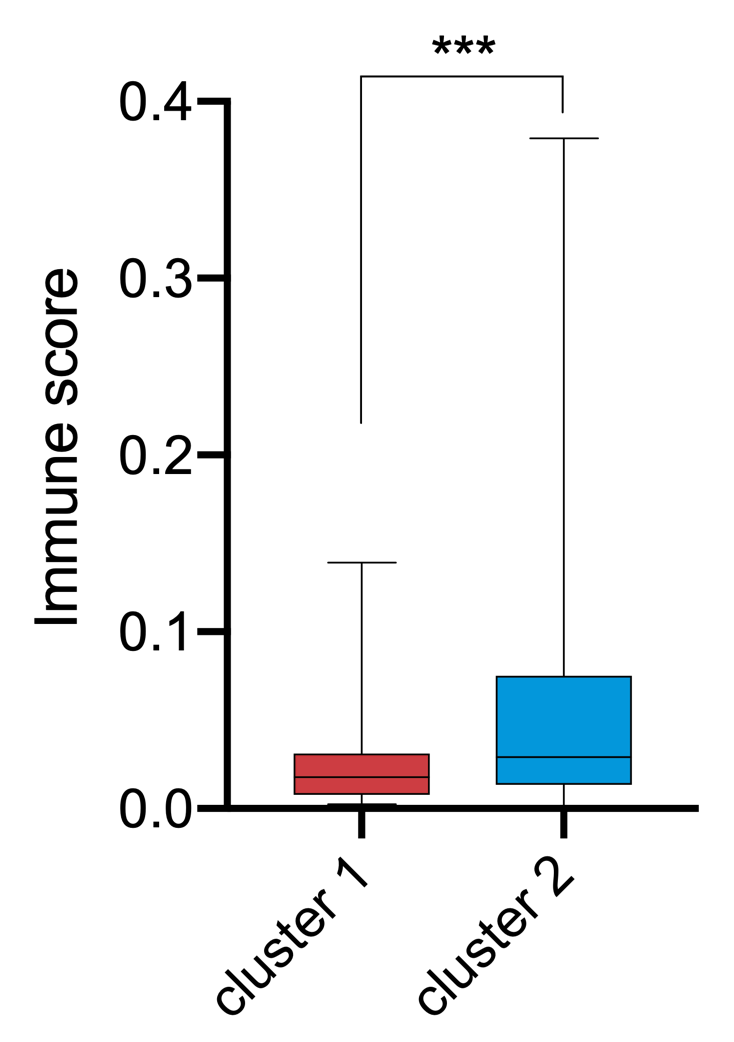

Supplement: Supplementary file 9 [file DataSheet5.ZIP › datasheet of Figure 5/TCGA/immune.tiff]

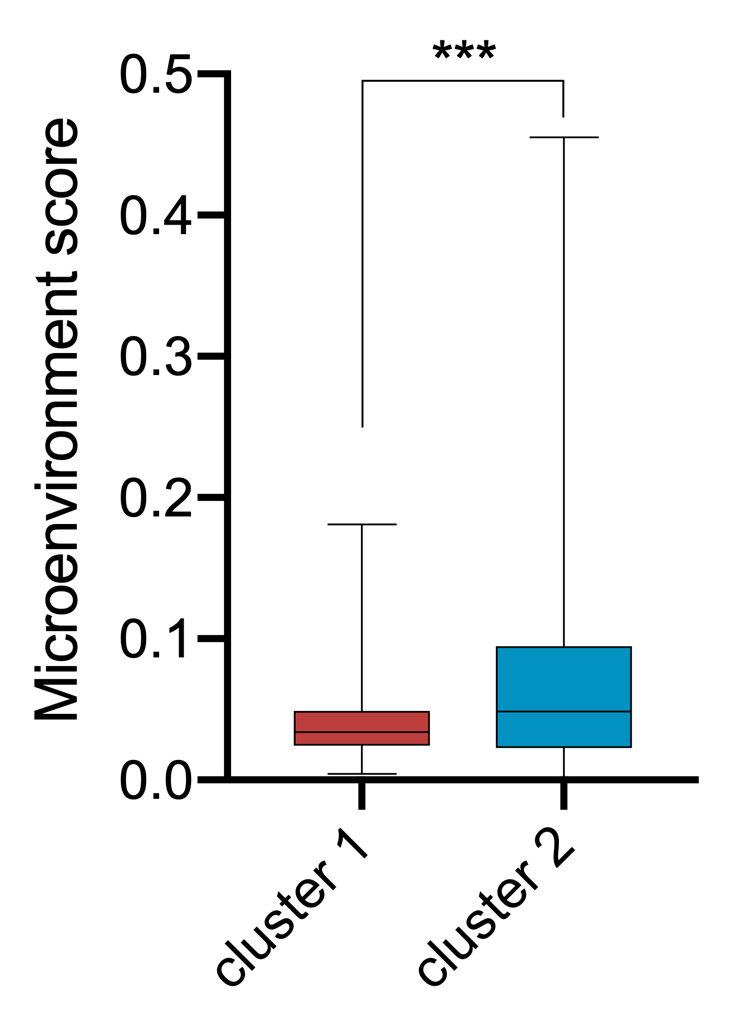

Supplement: Supplementary file 9 [file DataSheet5.ZIP › datasheet of Figure 5/TCGA/TME.tiff]

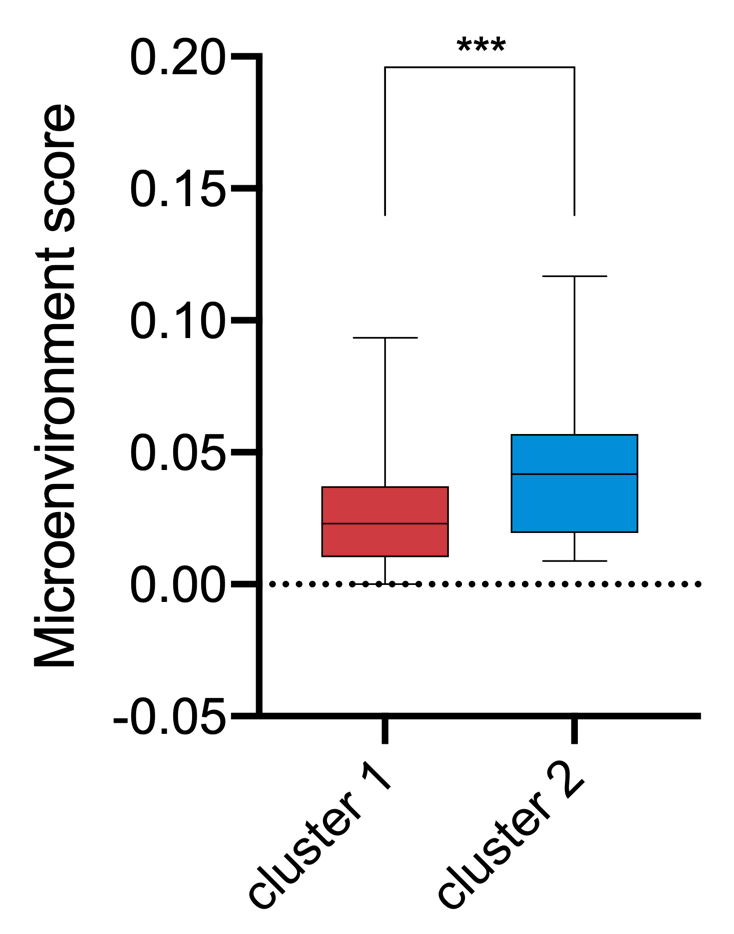

Supplement: Supplementary file 9 [file DataSheet5.ZIP › datasheet of Figure 5/GSE17536/geoTME.tiff]

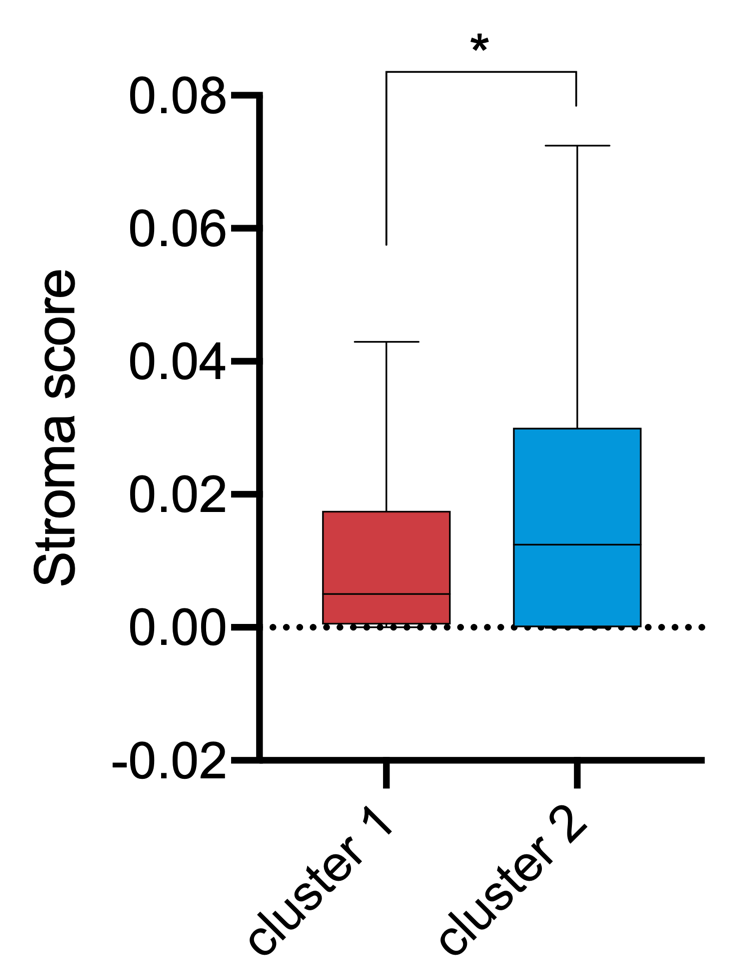

Supplement: Supplementary file 9 [file DataSheet5.ZIP › datasheet of Figure 5/GSE17536/GEOstroma.tiff]

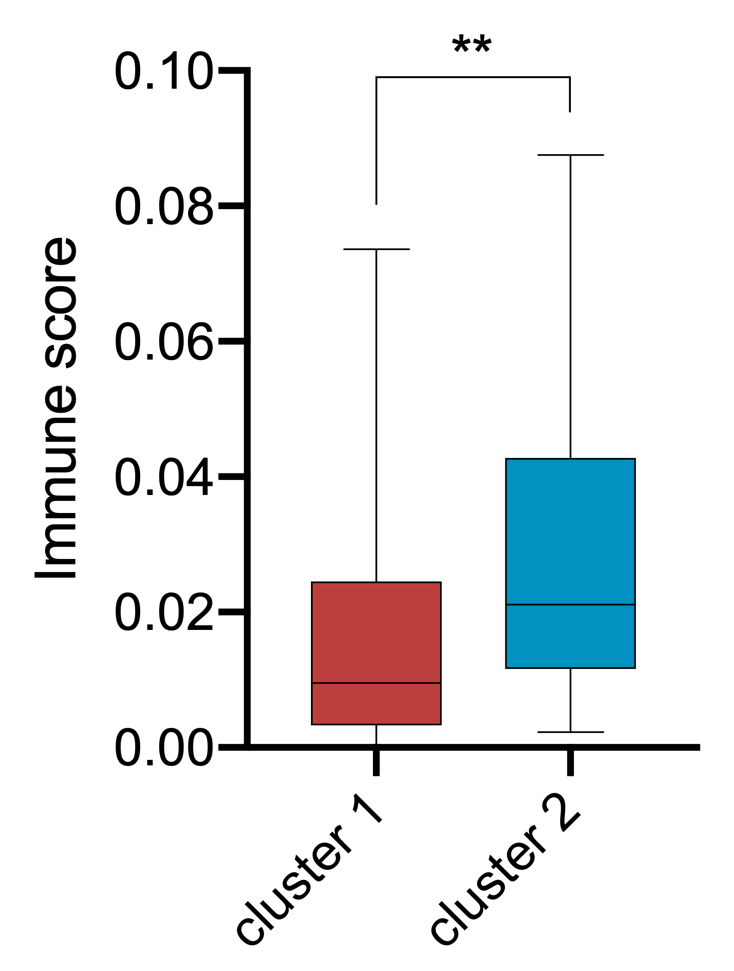

Supplement: Supplementary file 9 [file DataSheet5.ZIP › datasheet of Figure 5/GSE17536/GEOimmune.tiff]
