# Supplementary material for: Reclassifying TNM stage I/II colorectal cancer into two subgroups with different overall survival, tumor microenvironment, and response to immune checkpoint blockade treatment
Source: Front Genet. 2022 Sep 21;13:948920. doi: 10.3389/fgene.2022.948920 (PMC9532767; doi:10.3389/fgene.2022.948920)
Supplement: Supplementary file 4 [file Table1.DOCX]

Supplementary Figure 1


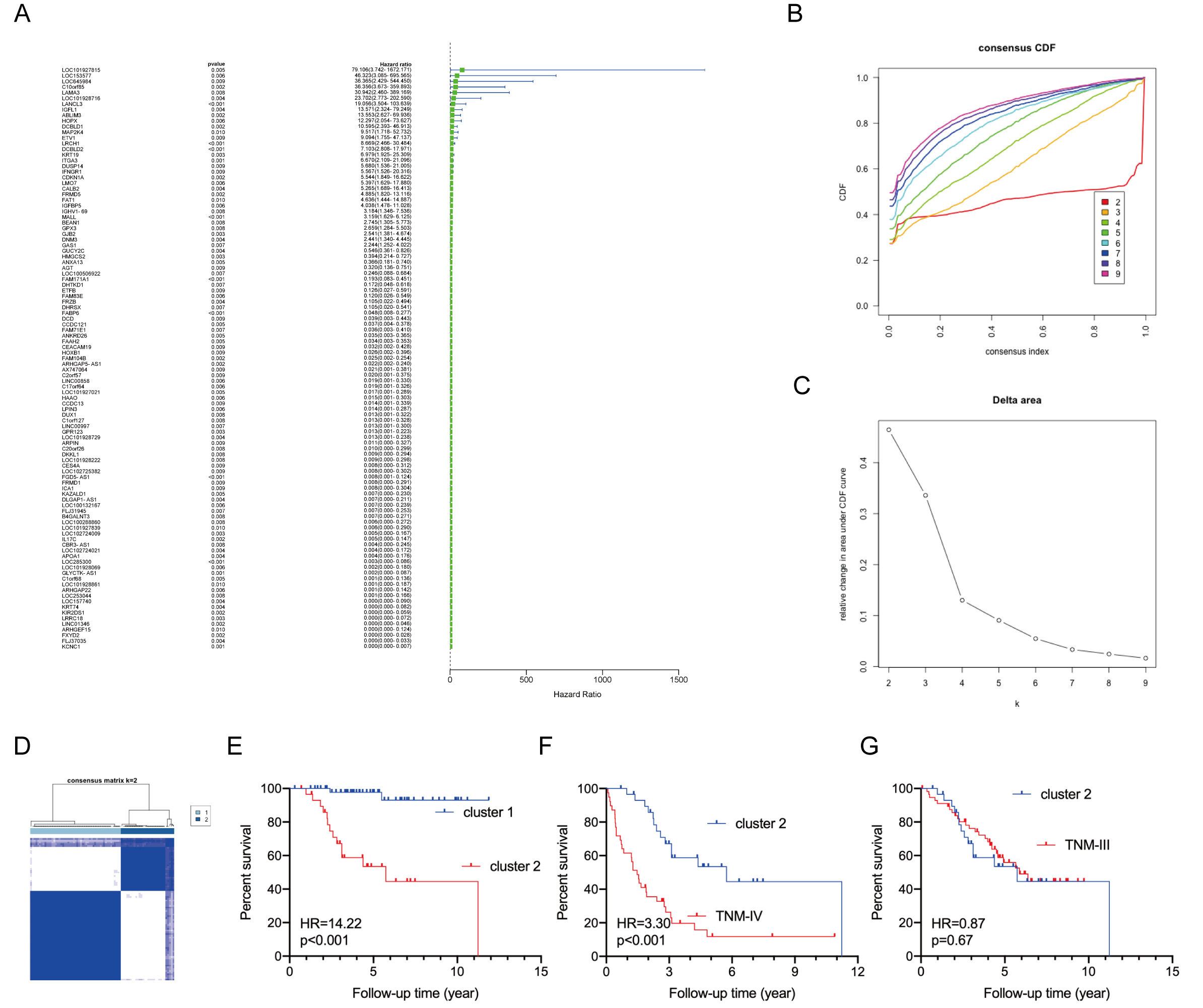


Validate the discrepant prognosis of TNM stage I/II CRC patients based on the GSE17536 dataset. (A) The forest plots of prognostic genes with p<0.001 identified by univariate Cox regression analysis. (B) The optimal number of clusters according to the consensus index. (C) The optimal clustering stability (k) determined by the proportion of ambiguous clustering measurements. (D) Consensus clustering analysis divided TNM stage I/II CRC patients into two subgroups. (E) The KM plot curves of CRC patients in cluster-1and cluster-2. (F) The KM plot curves of CRC patients in cluster-2 and patients at the TNM-IV stage. (G) The KM plot curves of CRC patients in cluster-2 and patients at the TNM-III stage.

Supplementary Figure 2


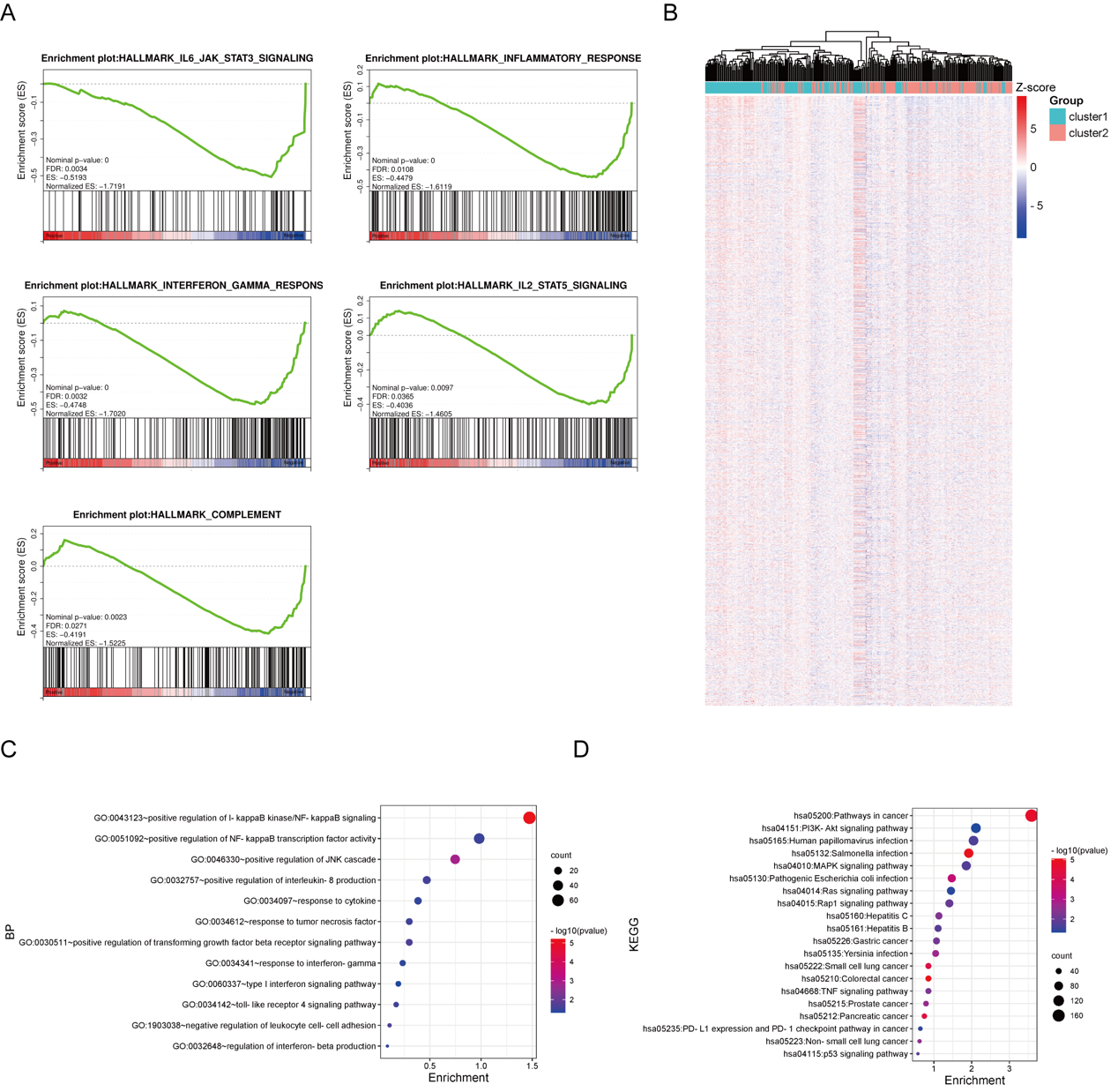


Functional enrichment analysis of DEGs between TNM stage I/II CRC patients in cluster-1 and cluster-2 based on the GSE39582 dataset. (A) The enriched immune-associated gene sets in CRC patients in cluster-1 revealed by GSEA analysis. (B) The heatmap of DEGs between CRC patients in cluster-1 and cluster-2. Red means high expression, and blue means low expression. (C) The biological process analysis of DEGs. (D) KEGG pathway analysis of DEGs.
