# Supplementary figures and images for: Reclassifying TNM stage I/II colorectal cancer into two subgroups with different overall survival, tumor microenvironment, and response to immune checkpoint blockade treatment (part 2 of 2)
Source: Front Genet. 2022 Sep 21;13:948920. doi: 10.3389/fgene.2022.948920 (PMC9532767; doi:10.3389/fgene.2022.948920)

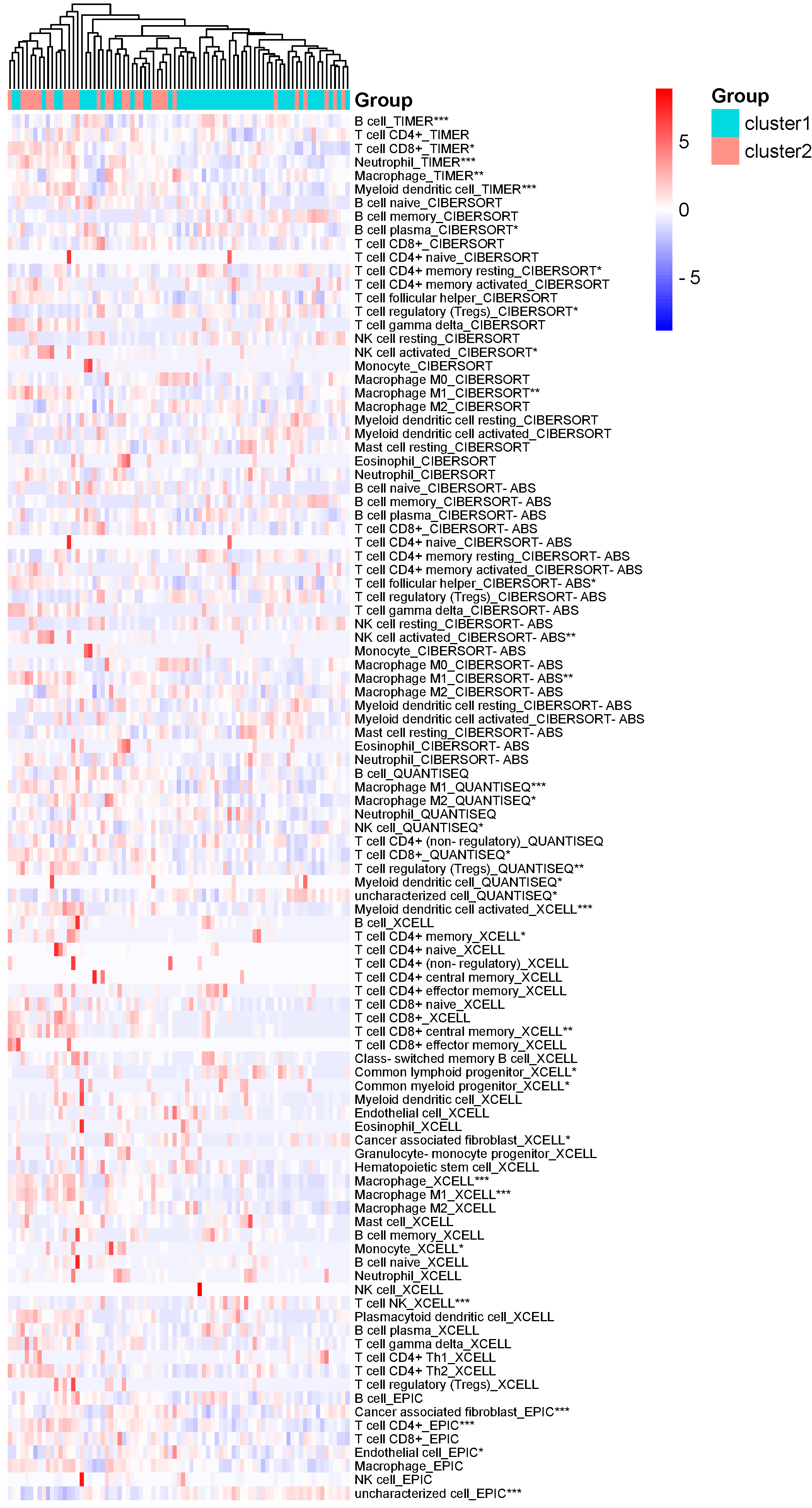

Supplement: Supplementary file 9 [file DataSheet5.ZIP › datasheet of Figure 5/GSE17536/1654048604.11.png]

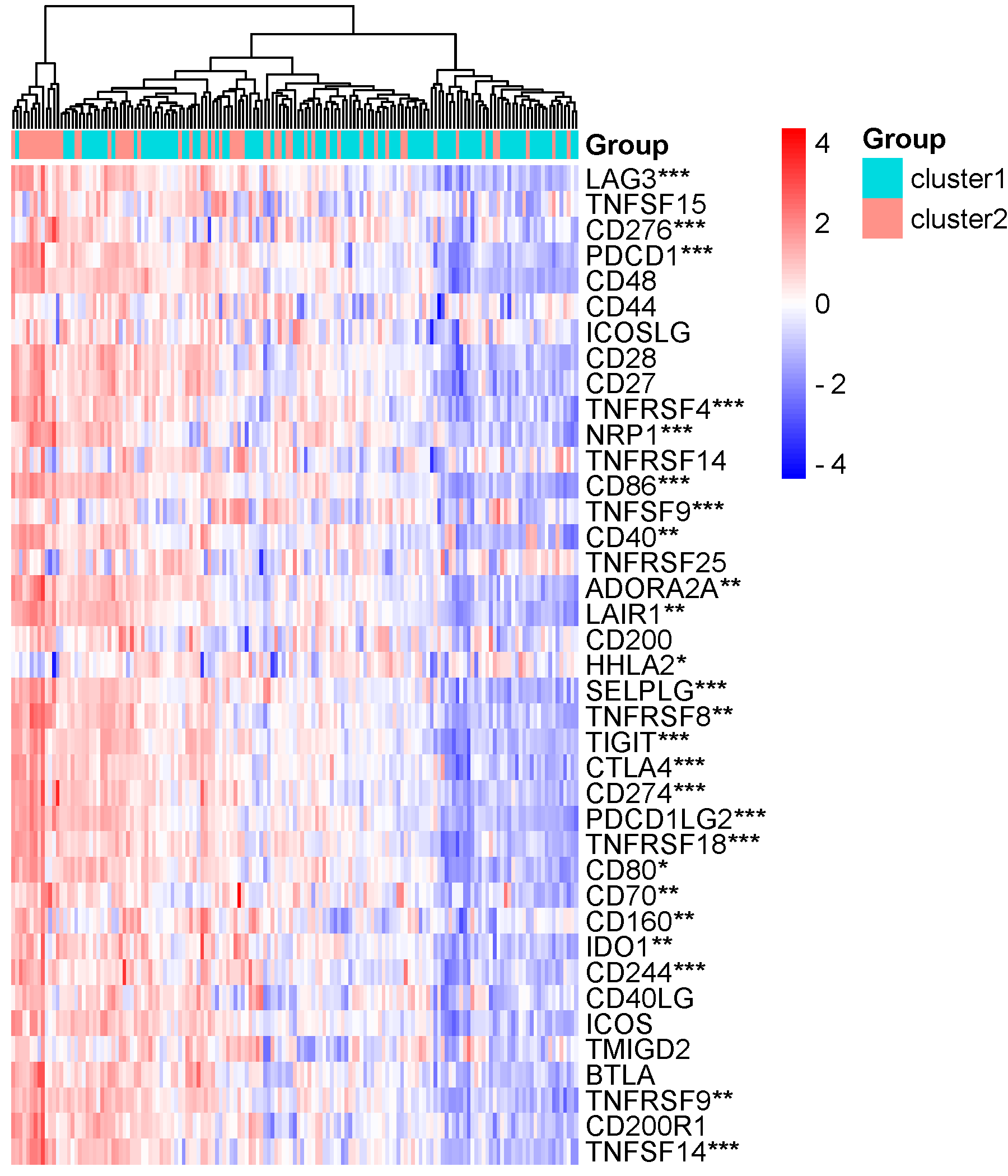

Supplement: Supplementary file 10 [file DataSheet7.ZIP › datasheet of Figure 7/TCGA.png]

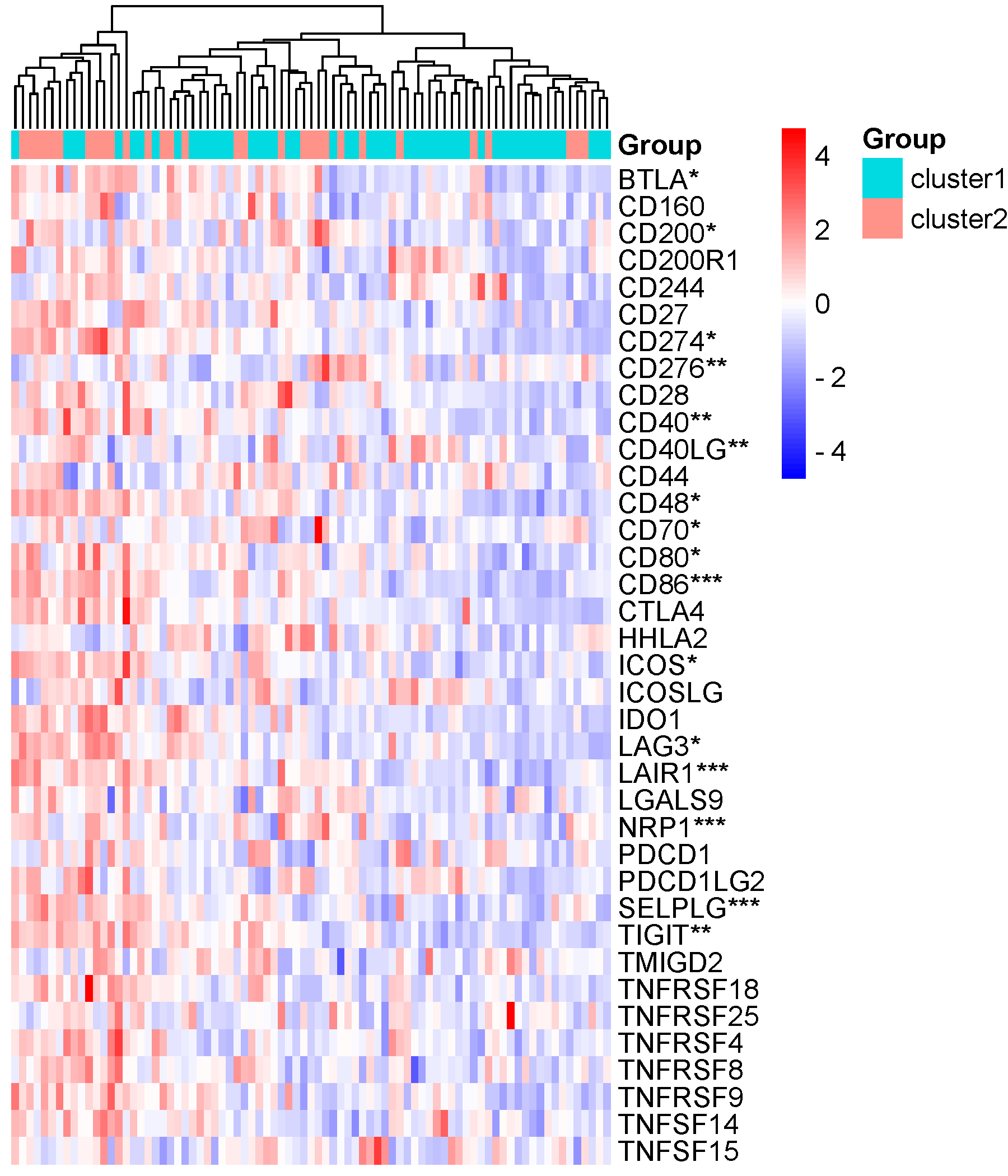

Supplement: Supplementary file 10 [file DataSheet7.ZIP › datasheet of Figure 7/GSE17536.png]

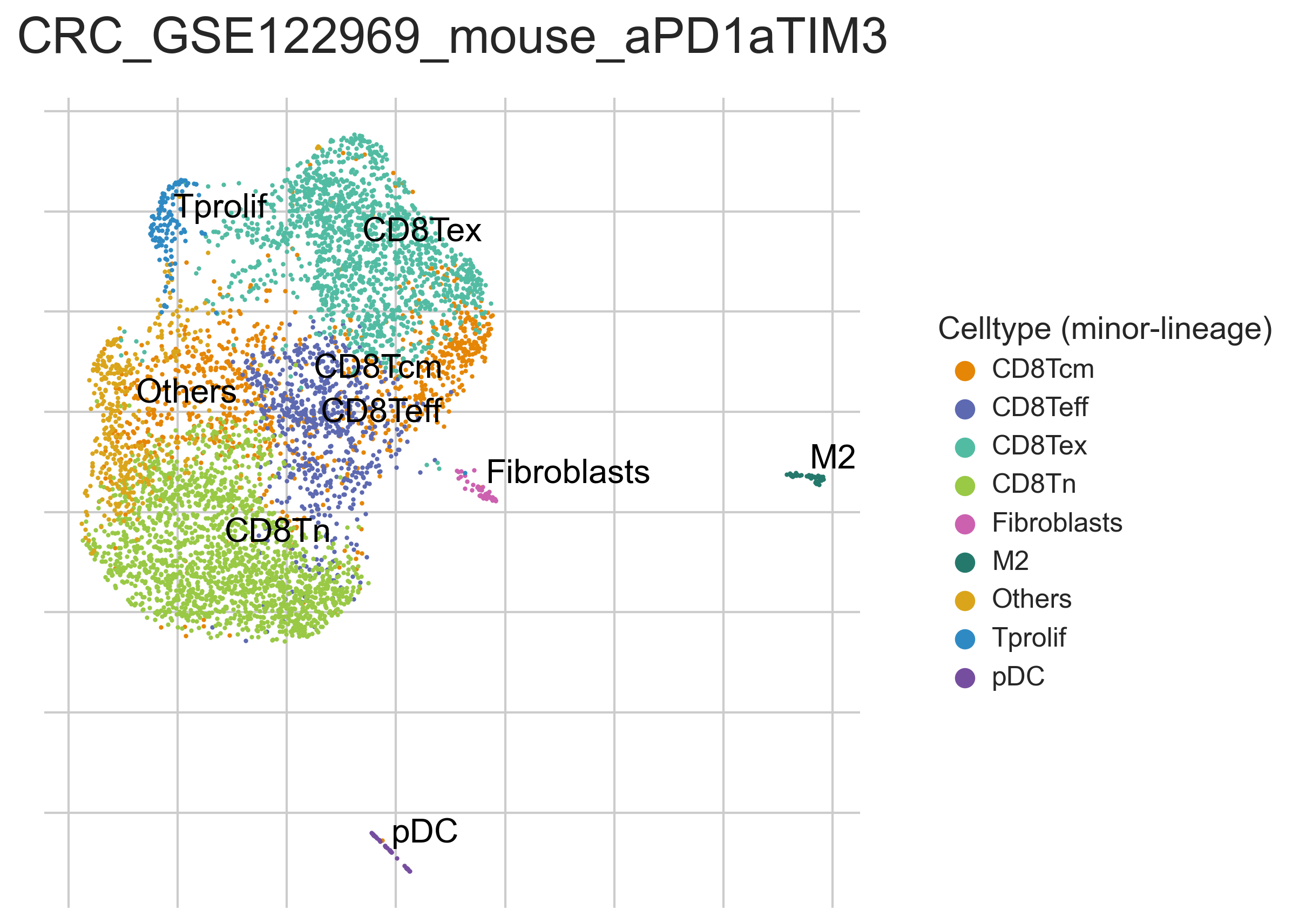

Supplement: Supplementary file 10 [file DataSheet7.ZIP › datasheet of Figure 7/CRC_GSE122969_mouse_aPD1aTIM3_umap_Celltype_subtype.png]

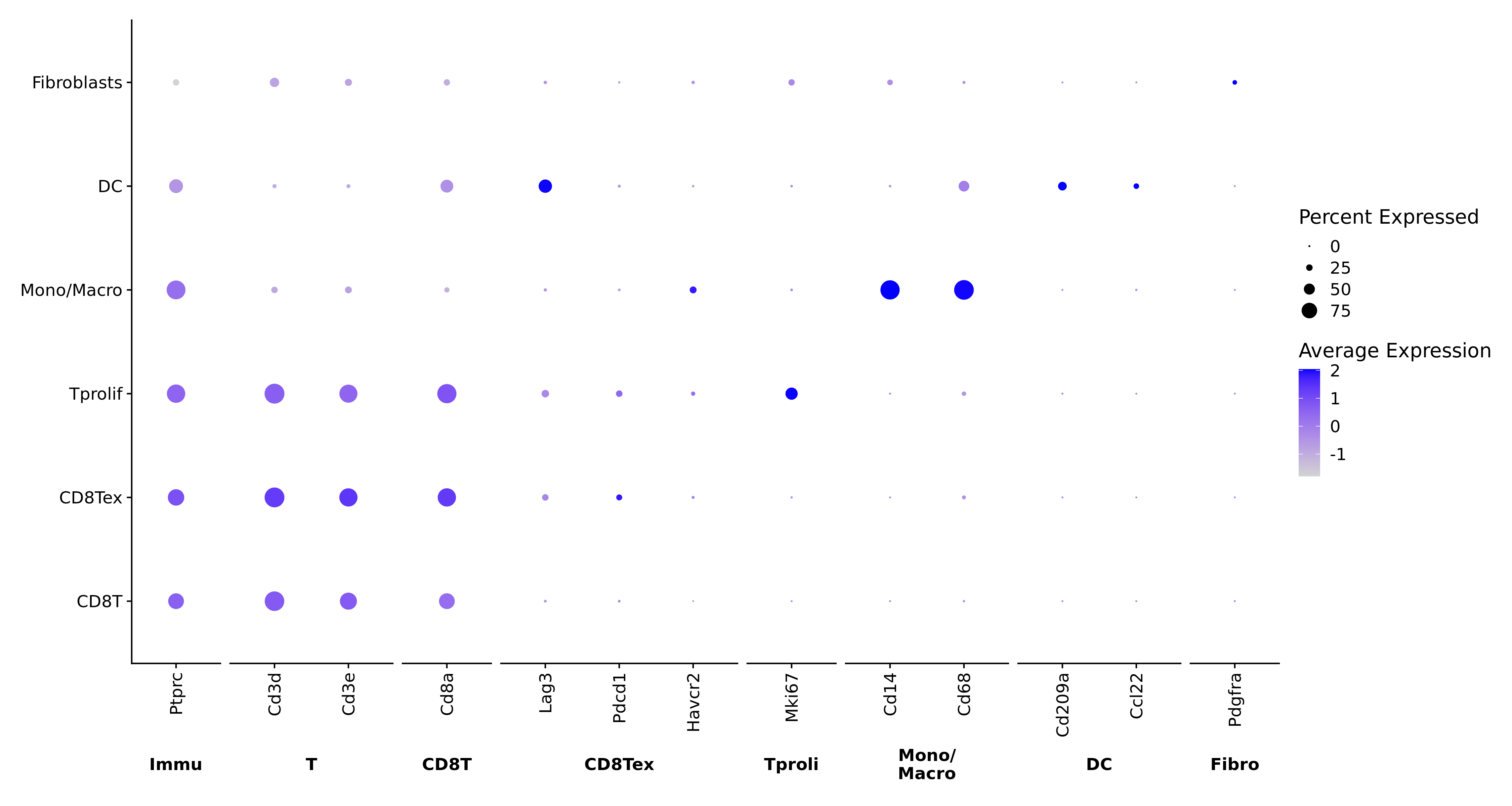

Supplement: Supplementary file 10 [file DataSheet7.ZIP › datasheet of Figure 7/CRC_GSE122969_mouse_aPD1aTIM3_Dotplot.png]

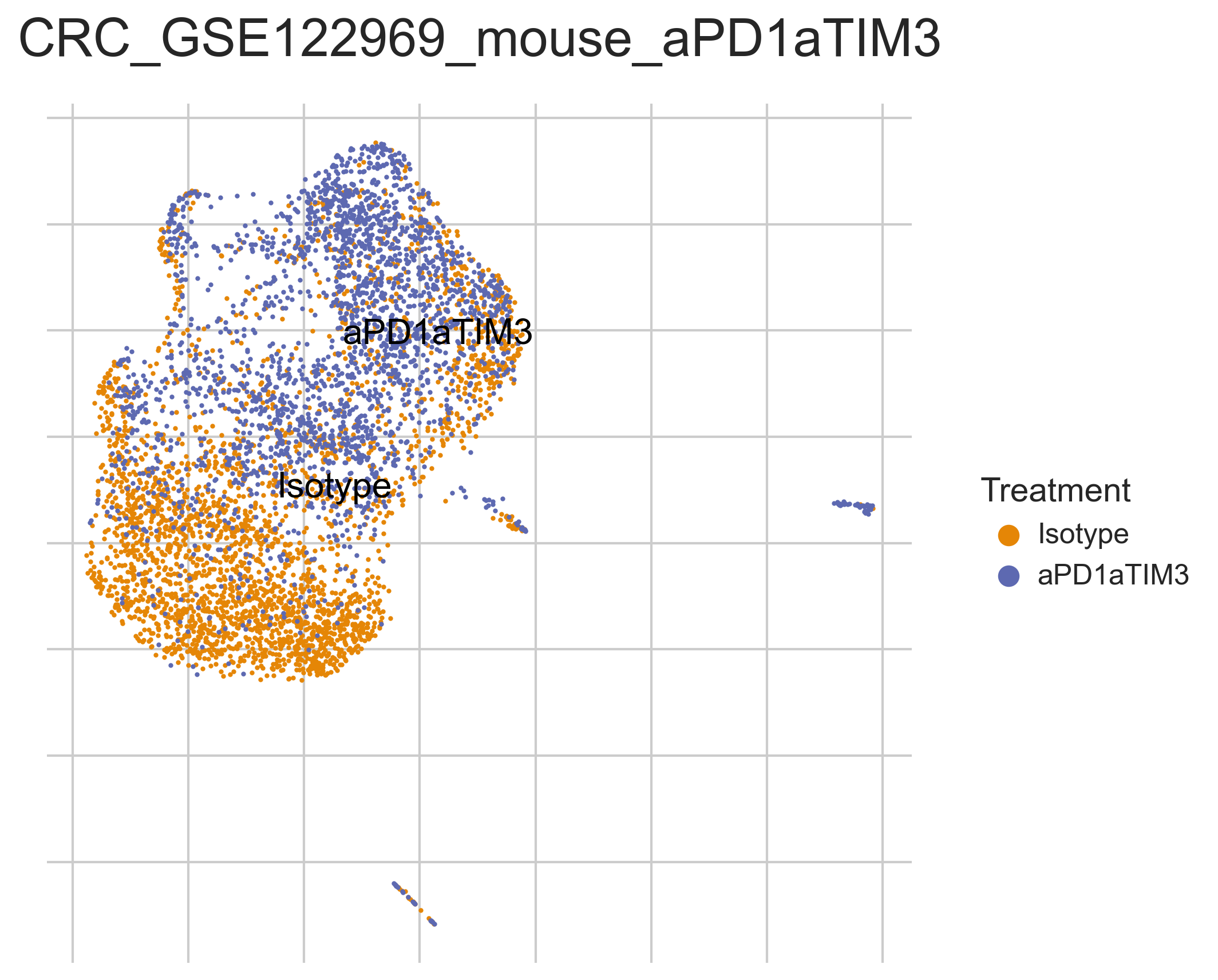

Supplement: Supplementary file 10 [file DataSheet7.ZIP › datasheet of Figure 7/CRC_GSE122969_mouse_aPD1aTIM3_umap_Treatment.png]
